# An antisense method for efficient exon skipping and its application to Duchenne muscular dystrophy

**DOI:** 10.1073/pnas.2606494123

**Published:** 2026-08-10

**Authors:** Pengchao Feng, Peng Gao, Shaodong Meng, Yunxia Yuan, Adrian R. Krainer, Yimin Hua

**Affiliations:** ^a^Nanjing Antisense Biopharm, Nanjing, Jiangsu 210023, China; ^b^ Jiangsu Key Laboratory for Molecular and Medical Biotechnology, College of Life Sciences, Nanjing Normal University, Nanjing, Jiangsu 210023, China; ^c^ Cold Spring Harbor Laboratory, Cold Spring Harbor, NY 11724; ^d^Guangzhou Asocura Pharmaceuticals, Guangzhou, Guangdong 510535, China

**Keywords:** bipartite antisense oligonucleotides, Duchenne muscular dystrophy, exon skipping

## Abstract

Antisense-mediated exon skipping has been used to treat Duchenne muscular dystrophy (DMD) by restoring the *DMD* reading frame to express partially functional dystrophin. However, difficulty in designing effective antisense oligonucleotides (ASOs) has hindered the development of clinically effective therapies. Here we describe a robust bipartite ASO design, termed 5′ splice site decoy (5D)-ASO, in which a short tail sequence is employed to enhance the splicing repression exerted by an antisense moiety. The tail carried by the antisense moiety to an exon of interest in a target pre-mRNA interferes with the recognition of the exon’s 5′ splice site by U1 snRNA and markedly enhances exon skipping, compared to tail-less ASOs. 5D-ASO has broad applicability, based on multiple genes tested. Particularly, an 8-nt tail, when appended to sequences targeting *DMD* exon 51, elicited a pronounced increase in exon skipping in mouse models, restored dystrophin expression in muscle tissues and improved the phenotype, without obvious signs of toxicity. The lead ASO further demonstrated a marked exon-skipping effect and an overall safe profile in monkeys. Our data establish a valuable platform technology for RNA-targeted therapeutics.

## Significance

Exon-skipping antisense oligonucleotides (ASOs) have been approved for the treatment of Duchenne muscular dystrophy (DMD). However, conventional exon-skipping ASO designs often lack the potency required for robust therapeutic outcomes. In this study, we developed a bipartite-ASO technology using a short 5′-splice site decoy sequence that strongly enhances exon-skipping efficiency. In the case of *DMD* exon 51 skipping, our lead bipartite ASO achieved potent exon-skipping activity in vivo, while maintaining a favorable safety profile. These results indicate that the bipartite-ASO technology is an effective platform for exon-skipping therapeutics.

Exon skipping has long been pursued as an approach to treat disease. It works in several scenarios. First, it can disrupt gene function by targeting an essential symmetric exon (1)—whose length is a multiple of three nucleotides—to generate an internally deleted protein isoform, or an asymmetric exon to cause a frameshift and generate a downstream-truncated isoform (and/or in some cases to trigger nonsense-mediated mRNA decay). It is commonly the case that shortened isoforms exert dominant-negative effects on their wild-type protein counterparts (2, 3). Second, exon skipping can be manipulated to achieve gain of function. Some exons encode peptides with protein localization signals and thus, skipping them generates isoforms with distinct localization, such that they retain their original enzymatic or ligand-binding activities, but with distinct consequences (4, 5). Third, exon skipping can rescue loss-of-function or eliminate gain-of-function of genes with frameshift, nonsense, missense, or repeat-expansion mutations, by correcting the reading frame or removing detrimental sequences (6, 7). Finally, exon skipping is a way to treat diseases associated with poison exons, either natural or activated by mutation or abnormal expression of splicing factors. For example, zorevunersen is an antisense oligonucleotide (ASO) used to rescue *SCN1A* haploinsufficiency in Dravet syndrome through skipping of a natural poison exon to upregulate the functional protein expressed from the wild-type allele (8).

ASOs are a powerful tool to promote exon skipping. ASOs are short, synthetic, single-stranded nucleic acid analogs, which—for the purpose of exon skipping—can be designed to mask positive splicing signals in pre-mRNA. ASOs targeting exonic sequences are often more effective than those targeting the 5′ or 3′ splice sites (5′ss and 3′ss) (9). Indeed, four phosphorodiamidate morpholino oligomers (PMOs) in clinical use, i.e., eteplirsen, golodirsen, viltolarsen, and casimersen, which treat DMD by exon skipping to restore the reading frame, are all exon-internal ASOs (7), as is zorevunersen (8). ASOs must be chemically modified to acquire drug-like properties, such as enhanced stability. Other frequently used chemistries include 2′-*O*-methyl (OMe) and 2′-*O*-methoxyethyl (MOE) modifying the ribose moiety, and phosphorothioate (PS) replacing the phosphodiester backbone.

Recent years have witnessed important advances in ASO-based therapeutics. However, due to the lack of effective tissue-specific delivery systems for most cell types, traditionally designed exon-skipping ASOs often fail to show sufficient efficacy in disease-affected tissues, particularly extrahepatic ones, which is one of the major hurdles that hamper their translation into the clinic. One approach to overcome this limitation is to enhance the potency of ASOs through optimized design. Previous studies reported bifunctional or bipartite ASOs comprising an antisense portion (AP) and a regulatory tail that recruits splicing repressors (10, 11), but to our knowledge, they have not been tested for exon skipping in vivo. For methods involving nucleic acid–protein binding, the chemical modifications necessary for stability may in some cases compromise the recognition of tail sequences by their cognate RNA-binding proteins.

Here, we describe a bipartite ASO method for efficient exon skipping that uses a short nucleotide sequence as a tail. The tail accommodates various backbone and sugar chemistries, because its mechanism relies on ASO-RNA base pairing, rather than on direct ASO–protein interaction. This method demonstrated broad applicability across a cohort of tested genes. Particularly, an 8-nt tail, attached to the 5′ end of antisense sequences targeting *DMD* exon 51, robustly enhanced its skipping in skeletal and cardiac muscles, with efficient dystrophin restoration in genetically modified mice after systemic administration. Furthermore, the lead ASO showed marked exon skipping and a favorable safety profile in cynomolgus monkeys, highlighting the approach as a powerful tool in antisense therapeutics.

## Results

### Identification of Short Sequences That Improve ASO-Mediated Exon Skipping in *SMN1/2*.

**Fig. 1.**
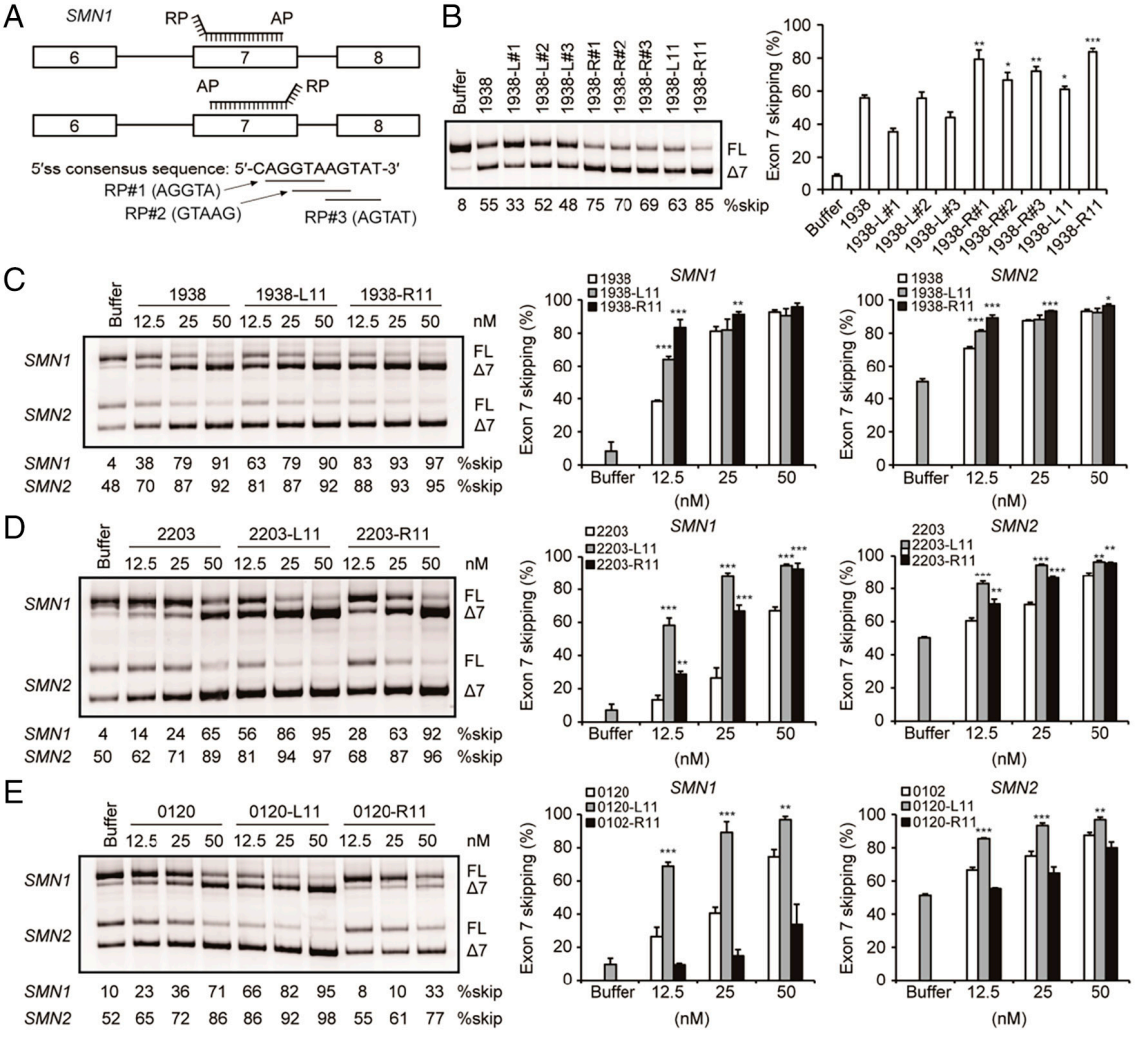
Identification of short inhibitory sequences in bipartite ASOs that enhance *SMN1/*2 exon 7 skipping. (*A*) Schematic design of bipartite ASOs comprising an AP and a regulatory portion (RP) to enhance exon 7 skipping of the *SMN1/2 genes*. 1938, which targets nucleotides 19 to 38 in *SMN1/2* exon 7, was used as an example of AP. Three inhibitory RP pentamers (#1, 2, and 3) resembling 5′ss sequences are shown below. (*B*) Effects of bipartite ASOs with one of the three pentamers or the 11-nt 5′ss consensus sequence as RP, and with 1938 as AP on exon 7 splicing of an *SMN1* minigene. Each ASO at 5 nM was cotransfected with 500 ng *SMN1* minigene plasmid into HEK293 cells. L#1, L#2, L#3, and L11: four RP sequences added to the 5′ end; R#1, R#2, R#3, and R11: sequences added to the 3′ end. (*C*–*E*) Effects of 5D ASOs compared to decoy-less ASOs on exon 7 splicing of the endogenous *SMN1/2* genes in HEK293 cells posttransfection at three indicated concentrations. 2203 and 0120 are APs complementary to sequences in introns 6 and 7, respectively. PCR products were digested with DdeI to distinguish their origin (*SMN1* or *SMN2*). For (*B–E*), exon 7 splicing was assessed by sqRT-PCR, and quantitation of exon 7 skipping from three independent experiments is shown as histograms on the right. Buffer, transfection without ASO as negative control; FL, full-length; Δ7, exon 7-skipped. *P < 0.05, **P < 0.01, ***P < 0.001, all compared with the corresponding AP-only ASOs.

We recently reported multiple pentameric sequences that markedly modulate *SMN2* exon 7 splicing when positioned downstream of the 5′ss (12). Among them, AG was identified as the only enriched inhibitory dinucleotide, which prompted us to test if AG-containing pentamers may also enhance the activity of ASOs designed for exon skipping. We synthesized a 20-mer MOE/PS-modified ASO, named ASO1938, which targets the central purine-rich element of exon 7, as the AP. We tested 34 AG-containing pentamers attached to the 5′ or 3′ end of ASO1938 cotransfected with an *SMN1* minigene to examine their effects on exon 7 skipping in HEK293 cells (13). “L” represents a sequence added to the 5′ end and “R” one added to the 3′ end of the ASO. Each ASO at 5 nM and the minigene plasmid were transiently cotransfected with Lipofectamine into cells, and splicing was analyzed by semiquantitative fluorescent RT-PCR (sqRT-PCR). ASO1938 promoted exon 7 skipping from 8% (buffer) to 56%. We observed that three 5-nt sequences (AGGTA, GTAAG, and AGTAT) further enhanced exon skipping to 79%, 67%, and 72%, respectively, when added to the 3′ end (Fig. 1 *A* and *B*). Interestingly, these pentamers are part of the 11-nt sequence CAGGTAAGTAT that comprises the 9-nt consensus 5′ss and is fully complementary to the free 5′ end of U1 small nuclear RNA (snRNA). Therefore, we asked if the 11-nt sequence serves as a better tail. Indeed, this sequence potently boosted exon skipping to 84% when attached to the 3′ end (R11) of ASO1938, while it had a moderate effect when attached to the 5′ end (L11), which was not seen for the pentamers.

To test the possibility that the tails exert their effects by base-pairing to U1 snRNA, we performed a suppressor U1 snRNA study, in which a vector overexpressing an U1 snRNA mutant (U1 mut) that perfectly base-pairs to the 5′ss of *SMN1/2* exon 7 was transfected into HEK293 cells and promoted exon 7 inclusion, as expected (*SI Appendix*, Fig. S1). We then tested an 11-nt tail complementary to U1 mut (L11mut), which markedly enhanced ASO1938-mediated exon 7 skipping, compared with L11 in U1 mut-expressing cells, whereas it exerted no or inhibitory effects in vector-free or wild-type U1 snRNA-overexpressing cells. These data suggest that a short sequence resembling an optimal 5′ss can serve as a decoy to repress splicing by misdirecting U1 snRNP. Here we dubbed the short tail “5′ss decoy” (or “5D” for short) based on its effect. Similarly, we named bipartite ASOs with a 5′ss tail “5D ASOs,” and the method “5D-ASO.”

We next assessed whether the exon-skipping effect of 5′ss decoys can be recapitulated for the endogenous *SMN1/2* genes, using ASO1938 and two additional sequences, ASO2203 and ASO0120, that target introns 6 and 7, respectively (14). The 11-nt decoy robustly enhanced exon skipping for all three ASOs (Fig. 1 *C*–*E*). However, in contrast to our results above with ASO1938, the decoy displayed a stronger boosting effect when attached to the 5′ end of both intron-targeting sequences than when attached to their 3′ end.

### 5′ss Decoys Potently Improve the Efficacy of *DMD* Exon-Skipping ASOs in Cultured Human Cells.

**Fig. 2.**
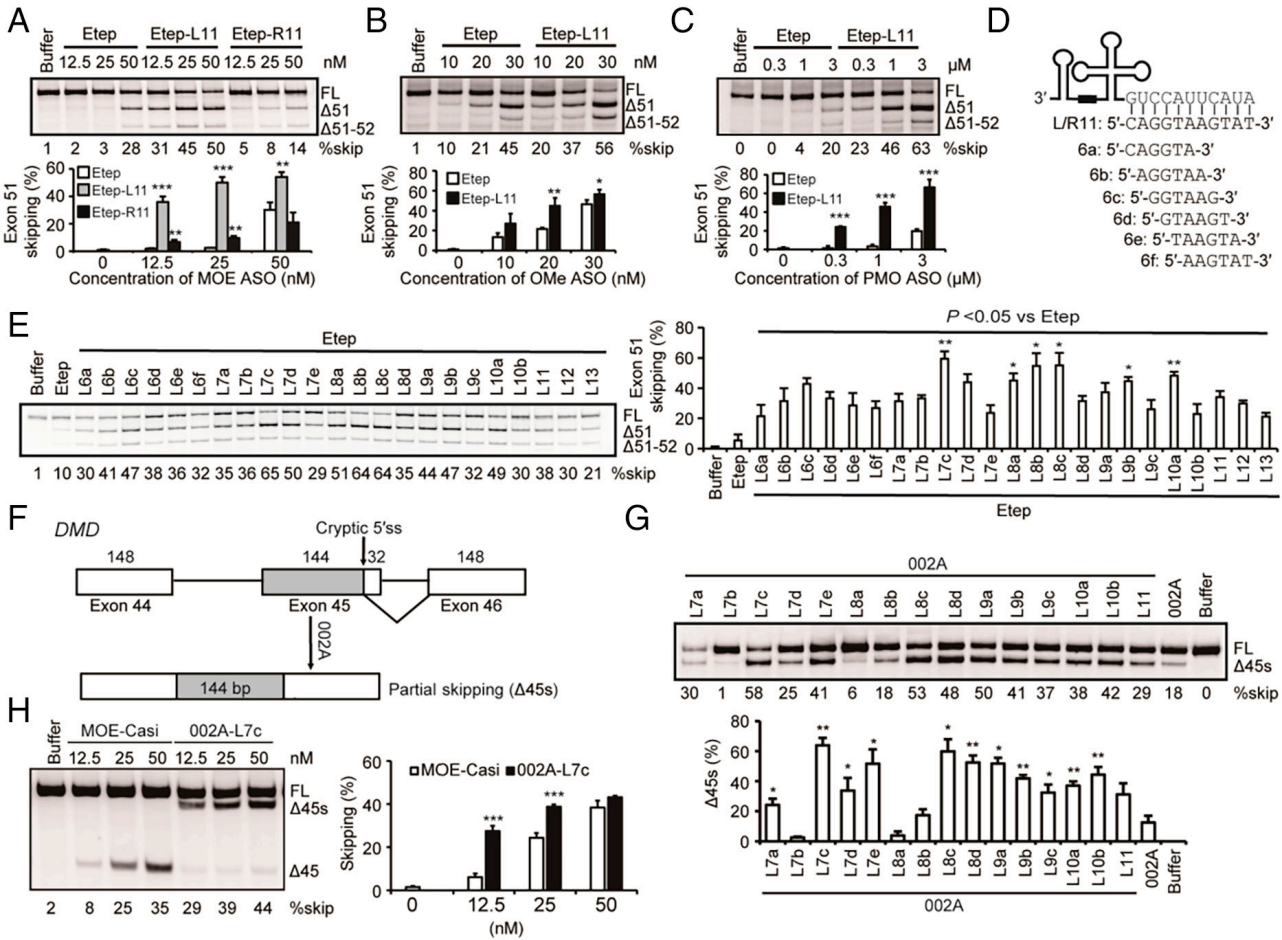
The effects of decoys with different lengths on enhancing exon skipping of *DMD*. (*A*) The effects of the 11-nt decoy attached to upstream (Etep-L11) or downstream (Etep-R11) of the MOE-version Etep on exon 51 skipping of the endogenous *DMD* gene were assayed in RD cells. Cells were transfected with each ASO at three concentrations (12.5, 25, and 50 nM). (*B*) The effects of the OMe-version Etep and Etep-L11 on *DMD* exon 51 splicing in RD cells. Cells were transfected as in (*A*). (*C*) The effects of the PMO-version Etep and Etep-L11 on *DMD* exon 51 splicing in RD cells. ASOs were delivered via gymnosis at 0.3, 1, and 3 μM. (*D*) The naming rule of decoys, taking all 6-nt decoys as examples, is illustrated along with the 11-nt decoy base-pairing to the free 5′ end of U1 snRNA. The same naming rule was applied to decoys of longer lengths. (*E*) Effects of decoys 6 to 13 nt long on *DMD* exon 51 skipping, when attached upstream of MOE-Etep. 25 nM of each ASO was transfected into RD cells. (*F*) Schematic of the use of the cryptic splice site in *DMD* exon 45. 002A triggers the use of the cryptic 5′ ss, leading to exclusion of the last 32 nt of the exon. (*G*) Effects of all 15 decoys 7 to 11 nt long on the use of the cryptic splice site of the endogenous *DMD* gene, when attached to the 5′ end of 002A. RD cells were transfected with 25 nM of each ASO. (*H*) Comparison of the exon-skipping efficacy between MOE/PS-modified casimersen (MOE-Casi) and 002A-L7c, using three concentrations (12.5, 25, and 50 nM) in RD cells. For panels (*A*–*C*, *E*, *G,* and *H*), RNA splicing was analyzed by sqRT-PCR; a representative gel image and quantitation of exon skipping (n = 3) are shown. Δ51, skipping of exon 51; Δ51-52, skipping of both exons 51 and 52. Δ45s: exclusion of a small portion (last 32 nt) of exon 45; Δ45: skipping of the whole exon 45. *P < 0.05, **P < 0.01, ***P < 0.001, compared with Etep in panels (*A*–*C*). *P < 0.05, **P < 0.01, compared with Etep-L11 in panel (*E*). *P < 0.05, **P < 0.01, all compared with 002A in panel (*G*). ***P < 0.001, compared with MOE-Casi in panel (*H*).

We next asked if 5D-ASO can be applied to eteplirsen (hereafter abbreviated as Etep) to enhance *DMD* exon 51 skipping (15). The 11-nt decoy was appended to either side of an MOE/PS-version of the 30-nt Etep (MOE-Etep) and tested in rhabdomyosarcoma (RD) cells. Cells were transiently transfected with ASOs at 12.5, 25, and 50 nM using Lipofectamine. Exon 51 splicing of the endogenous *DMD* gene was analyzed by sqRT-PCR. As shown in Fig. 2*A*, the decoy robustly promoted exon 51 skipping when attached to the 5′ end of MOE-Etep (Etep-L11). At 12.5 nM, the decoy increased exon skipping to 36% compared to 2% (MOE-Etep) and 1% (buffer). The effect was more moderate when the decoy was attached to the 3′ end (Etep-R11).

To corroborate that the method is not restricted to the use of MOE/PS modification, we tested Etep-L11 in its original version (i.e., PMO) and as an OMe/PS version. Decoy sequences were modified with the same chemistry as their corresponding exon-targeting part. Both PMO and OMe/PS chemistries showed marked boosting effects by the decoy (Fig. 2 *B* and *C*).

To further dissect which decoy sequences exert maximal effects, we designed all 20 possible decoys with lengths ranging from 6 to 10 nt that are fully complementary to different portions of the 11-nt 5′-end sequence of U1 snRNA. Decoys are named based on their length and the position of their complementary sequences in U1 snRNA. The lower case “a” stands for decoys hybridized to sequences ending at the 11th nucleotide G, “b” stands for decoys hybridized to sequences ending at the 10th nucleotide U, and so on (Fig. 2*D*). For example, “6b” means the 6-nt decoy AGGTAA (or AGGUAA) that exactly base-pairs to the sequence in U1 snRNA from positions 5 to 10 (UUACCU). We also tested two longer decoys complementary to the first 12 or 13 nt of U1 snRNA, respectively. In light of the data in Fig. 2*A*, we only tested decoys attached to the 5′ end of MOE-Etep. All ASOs were uniformly MOE/PS modified. Each ASO at 25 nM was transfected into RD cells. All 23 decoys were more effective in enhancing exon skipping compared to MOE-Etep (Fig. 2*E*), and six decoys (L7c, L8a, L8b, L8c, L9b, and L10a) were stronger than L11. The top three, L7c, L8b, and L8c, increased the percentage of exon 51 skipping from 5% on average (decoy-less MOE-Etep) to 55 to 59%. Six decoys (L6c, L7c, L8b, L8c, L9b, L10a) were further compared to L11 at three lower concentrations (10, 15, and 20 nM); L8c-attached MOE-Etep (Etep-L8c) consistently displayed the highest exon skipping percentages, whereas Etep-L6c had the lowest values (*SI Appendix*, Fig. S2).

Aside from Etep, we also identified other exon 51-targeting sequences whose exon-skipping effects could be robustly boosted by a short decoy. For example, a 29-nt bipartite ASO, 000A-L8c, consisting of a 21-nt exon-targeting part and L8c, had a comparable effect to Etep-L8c (*SI Appendix*, Fig. S3).

Exon 45 skipping is suitable to treat about 9% of DMD patients (16, 17). We identified a 22-nt ASO, 002A, that activates a cryptic 5′ss in exon 45, resulting in only the last 32 nt being spliced out, instead of the whole 176-nt exon (Fig. 2*F*). This partial skipping strategy, preserving 144 nt that encode 48 amino acids, is suitable for relevant mutations located downstream of exon 45. We tested 5′ss decoys with lengths ranging from 7 to 11 nt attached to 002A. At 25 nM, 11/15 decoys attached to the 5′ end displayed a twofold to fivefold increase in splicing of the 32-nt shorter exon, compared to 002A assayed in RD cells, and the top two were L7c and L8c, which increased cryptic splicing from 12% (002A) to 64% and 60%, respectively (Fig. 2*G*).

We next compared the effectiveness of 002A-L7c with the MOE/PS version of casimersen (MOE-Casi) in RD cells. As shown in Fig. 2*H*, at 12.5, 25, and 50 nM, MOE-Casi gave 6%, 24%, and 38% full exon 45 skipping, respectively, whereas the 5D ASO exhibited a significantly enhanced effect, with 27%, 39%, and 43% shortening of the last 32 nt of the exon, respectively.

To test whether 5D-ASO can serve as a general method for exon skipping, we further evaluated several additional disease-related genes, and found it to be operative for multiple exons tested, including *APP* exon 17, *PKM* exon 10 (mutually exclusive with exon 9), *ATXN3* exon 10, *CEP290* exon 41, *MDM4* exon 6, and *ERBB2* exon 19 (*SI Appendix*, Fig. S4).

### Off-Target Analysis of the 5D-ASO Method Using Etep-L8c.

**Fig. 3.**
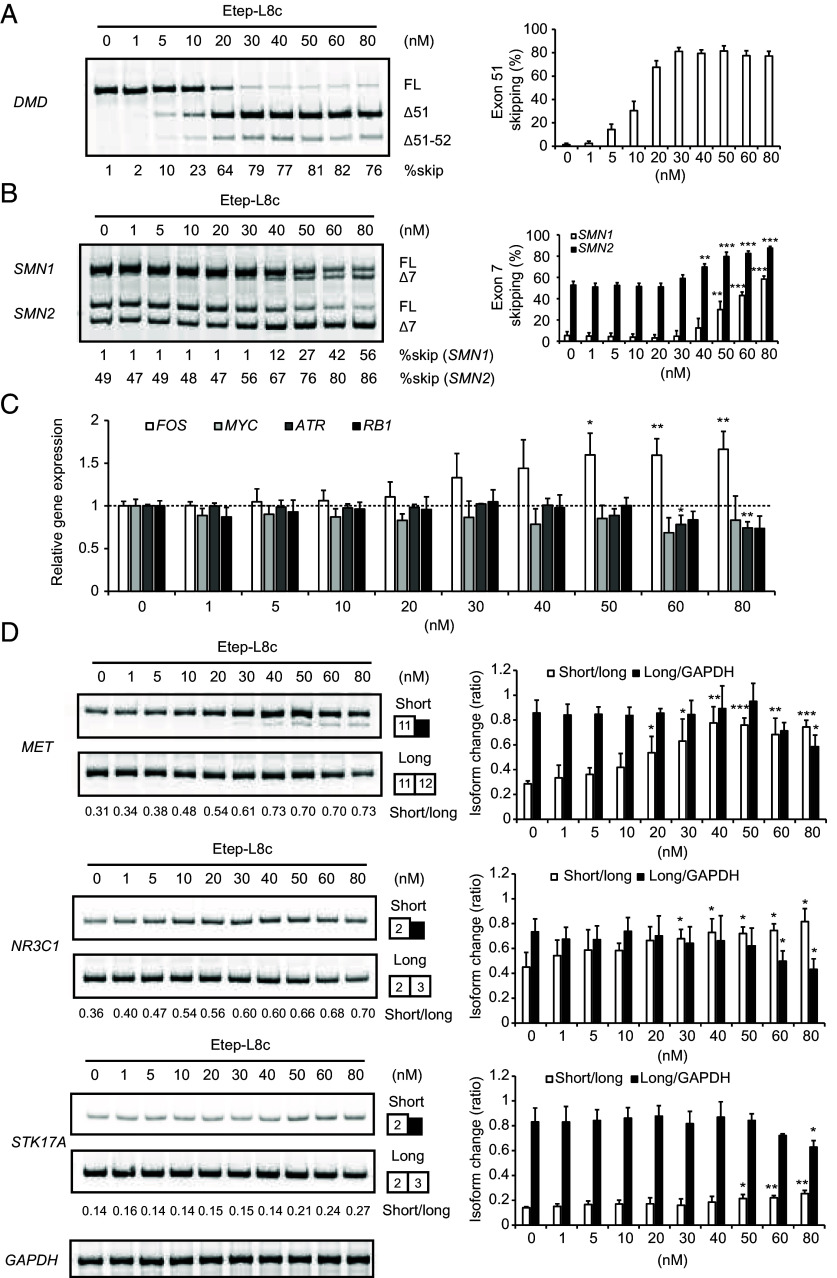
Off-target analysis of Etep-L8c in RD cells. Cells were transfected with Etep-L8c at a range of concentrations (0, 1, 5, 10, 20, 30, 40, 50, 60, and 80 nM). Three independent experiments were conducted and histograms of data were plotted. (*A* and *B*) Effects of Etep-L8c at the 10 concentrations on skipping of *DMD* exon 51 and *SMN1/2* exon 7 were assessed by sqRT-PCR. (*C* and *D*) Effects of Etep-L8c at the 10 concentrations on mRNA expression levels of *FOS*, *MYC*, *RB1,* and *ATR,* as well as PCPA activity of *MET*, *NR3C1,* and *STK17A*, were assessed by qRT-PCR or sqRT-PCR with *GAPDH* as internal control. *P < 0.05, **P < 0.01, ***P < 0.001, all compared with 0 nM (buffer control).

The main function of U1 snRNA is base-pairing to the 5′ss and initiating spliceosome assembly to remove introns (18). Another one of its functions is suppression of premature cleavage and polyadenylation (PCPA) of pre-mRNA to preserve transcriptome integrity, a process termed “telescripting” (19, 20). Thus, sequestration of U1 snRNA can affect gene expression through both splicing and PCPA (19–21). We explored whether there is a window of concentrations in which 5D ASOs exert maximal exon-skipping effects, without otherwise perturbing gene expression. To this end, we used a range of concentrations of Etep-L8c transfected into RD cells and examined expression alterations in *DMD* and multiple potential off-target genes. As shown in Fig. 3*A*, a significant increase in *DMD* exon 51 skipping was evident at 5 nM, and the maximal exon-skipping effect was reached at 30 nM. *SMN2* exon 7 splicing is sensitive to U1 snRNP levels (22, 23). We found that cells transfected with Etep-L8c at 30 nM or less showed no splicing changes in either *SMN1/2* genes, although exon 7 skipping started to increase at 40 nM for *SMN2* and 50 nM for *SMN1* (Fig. 3*B*). We also examined three other splicing events that were previously shown to be regulated by U1 snRNA levels (24); only one gene (*PRKAA2*) showed a subtle splicing change in 30-nM Etep-L8c-treated cells, whereas expression of the functional transcripts of all three genes (*PRKAA2*, *MRPL18*, and *CDC34*) was not affected (*SI Appendix*, Fig. S5).

A prior study used a 25-nt PMO to block the 5′ end of U1 snRNA and reported numerous transcriptome changes, including *FOS* and *MYC* as two of the most upregulated genes, and *ATR* and *RB1* as two of the most downregulated genes (24). We therefore examined mRNA-level changes of these four genes. *FOS* expression started to increase at 50 nM Etep-L8c, whereas *ATR* expression started to decrease at 60 nM, and expression of *MYC* and *RB1* was not altered at any tested concentration (Fig. 3*C*). We also analyzed three genes (*MET*, *NR3C1,* and *STK17A*) previously described to undergo PCPA upon U1 RNA inhibition (21, 24, 25). These PCPA-induced short isoforms were actually detectable without ASO treatment, and gradually increased starting at 20 (*MET*), 30 (*NR3C1*), or 50 nM (*STK17A*); however, the full-length transcript of each gene was not significantly reduced until the concentration of Etep-L8c reached 60 nM or greater (Fig. 3*D*). By comparison, Etep-L8c achieved 68% *DMD* exon 51 skipping at 20 nM.

### 5D ASOs Efficiently Induce *DMD* Exon 51 Skipping in Genetically Modified Mice.

To test whether 5D ASOs work in vivo, we generated mice with humanized *DMD* exon 51 (huEx51, strain C001775) and flanking intronic sequences. Male mice at ~8 wk of age were subcutaneously administered MOE-Etep at 200 mg/kg, or Etep-L8c at 100 or 200 mg/kg on days 0 and 2, with saline as a control (*SI Appendix*, Fig. S6*A*). Both ASOs were MOE/PS-modified. We harvested three skeletal muscles—diaphragm (DI), quadriceps femoris (QU), and triceps brachii (TR)—and heart at four time points: days 15, 30, 60, and 120; and we analyzed extracted RNA samples by sqRT-PCR (*SI Appendix*, Fig. S6*B*). MOE-Etep had no or negligible effects on exon 51 skipping in all muscle samples at all harvesting time points. In marked contrast, exon 51 skipping was observed in all skeletal muscles after Etep-L8c treatment. At most time points, the mean skipping percentage increased to over 20%, with the highest value being 50%, even when the administered dose of Etep-L8c was half that of MOE-Etep. Surprisingly, the effect of Etep-L8c steadily increased during the 4-mo period after treatment, and remained at the peak on day 120, suggesting high stability of MOE/PS modified ASOs after internalization by muscle cells.

We also performed pharmacokinetic analysis of Etep-L8c in an exon 52-deleted DMD mouse model (Δ52 model, *SI Appendix*, Fig. S7), which is similar to the previously described *mdx52* mice (26). After two intravenous injections at 100 mg/kg, mice were killed humanely at different time points, and blood, heart, diaphragm, and quadriceps samples were analyzed by liquid chromatography-mass spectrometry. ASO levels in the blood were reduced from the peak value (>2 × 10^6^ ng/mL) to 159.8 ng/mL at 24 h, and to 86.5 ng/mL at 360 h (15 d) postinjection. In contrast, 90 d postinjection, ASO levels remained at >14,300 ng/g in all tested muscle tissues (*SI Appendix*, Fig. S7), confirming the high stability of the ASO in muscle.

**Fig. 4.**
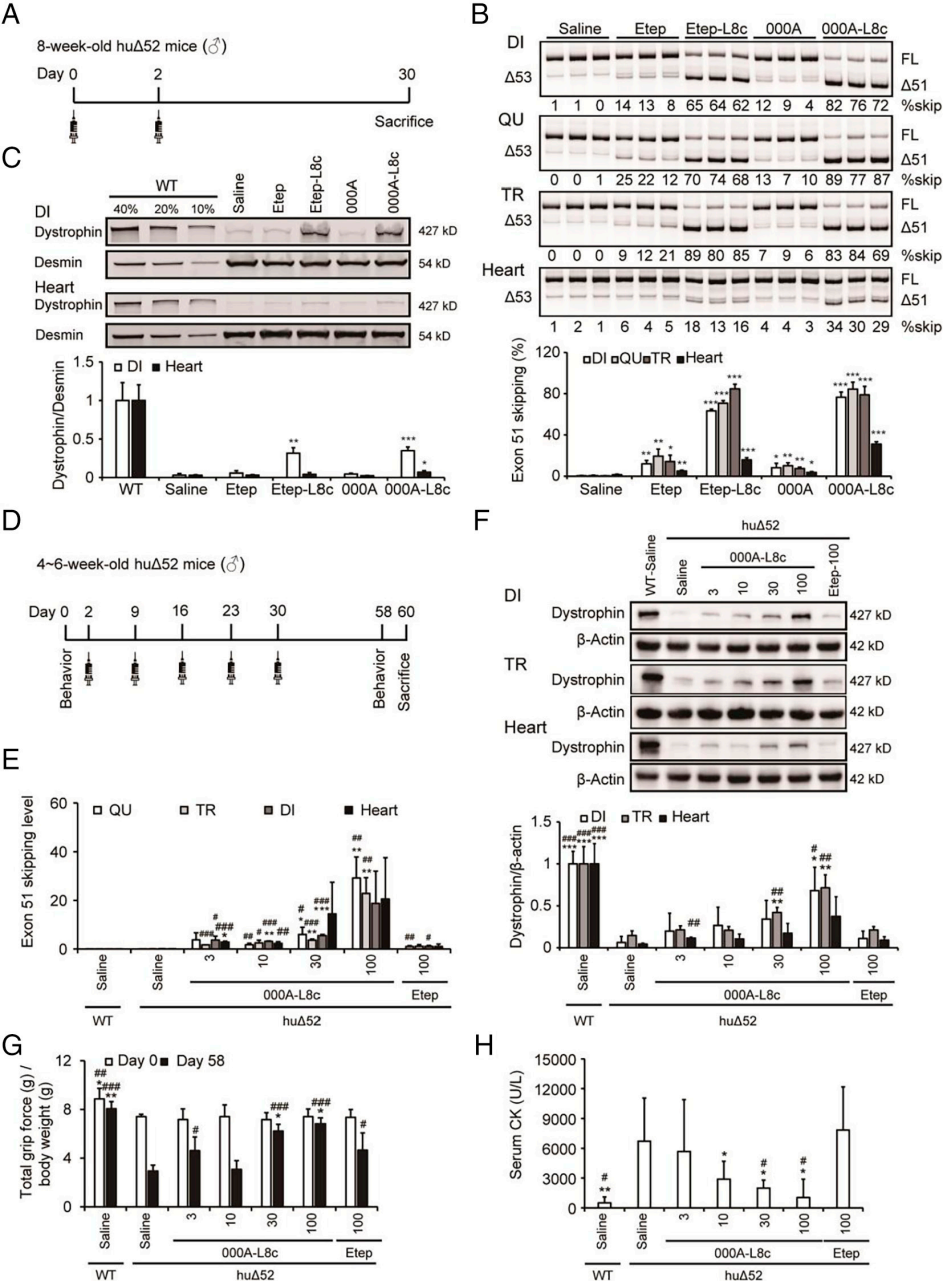
The effect of 000A-L8c on huΔ52 mice. (*A*) Schematic of ASO dosing schedule in the huΔ52 mouse model for the comparison of efficacy among different ASOs. ASOs were subcutaneously administered to 8-wk-old male huΔ52 mice at 100 mg/kg on days 0 and 2. On day 30, huΔ52 mice were killed humanely and muscle tissues were collected. (*B*) sqRT-PCR showed remarkable enhancement of exon 51 skipping in DI, QU, TR, and heart tissues by 000A-L8c or Etep-L8c, compared to 000A or MOE-Etep. (*C*) Western blot analysis revealed pronounced restoration of dystrophin by 000A-L8c (DI and heart) and Etep-L8c (DI). (*D*) Schematic of ASO dosing schedule of 000A-L8c and MOE-Etep in the huΔ52 mouse model. ASOs were administered to 4- to 6-wk-old male huΔ52 mice at four different doses for 000A-L8c (3, 10, 30, and 100 mg/kg) and at 100 mg/kg for MOE-Etep on days 2, 9, 16, 23, and 30 via the tail vein, and animals were sacrificed on day 60 for tissue collection. (*E*) qRT-PCR analysis of exon 51 skipping in skeletal muscles and heart. Data were normalized to *GAPDH* and presented as fold changes. (*F*) Western blot analysis revealed dose-dependent restoration of dystrophin by 000A-L8c in DI, TR, and heart. β-actin was used as internal control. (*G*) Grip strength testing for all limbs was performed on days 0 and 58. (*H*) Treatment with 000A-L8c reduced serum CK levels after five doses (10 mg/kg or above). Δ53: exon 53-skipped transcript. For each group, *n* = 3 except panel (*G*) (*n* = 5). *P < 0.05, **P < 0.01, ***P < 0.001, compared with saline/huΔ52 (panels *B* and *C*) or MOE-Etep (panels *E*–*H*). ^#^*P* < 0.05, ^##^*P* < 0.01, ^###^*P* < 0.001, compared with saline/huΔ52.

Encouraged by the above target-engagement data, we generated a DMD mouse model (huΔ52) with a large genomic fragment (introns 49 to 53) humanized and exon 52 deleted. The deletion causes a frameshift starting in exon 53, with a premature termination codon (UGA) at positions 2 to 4 in the exon. We injected male mice subcutaneously with 100 mg/kg MOE-Etep, Etep-L8c, 000A, or 000A-L8c, or saline on days 0 and 2. We sacrificed the mice on day 30 and collected muscle tissues for splicing analysis (Fig. 4*A*). Weak baseline exon 53 (but no exon 51) skipping was detectable. Treatment with decoy-less ASOs moderately increased exon 51 skipping from 0% (saline) to between 12 and 20% (MOE-Etep) or to between 7 and 10% (000A) in three skeletal muscles, with minimal effects in heart. By contrast, Etep-L8c and 000A-L8c induced robust exon 51 skipping in skeletal muscles, with the maximal percentage reaching ~90% and with moderate effects in heart, where the value increased from 1% (saline) to 16% (Etep-L8c) and 31% (000A-L8c) (Fig. 4*B*).

The exon 52-deleted mRNA isoform expressed from the mutated *Dmd* gene in the huΔ52 model generates a C-terminally truncated dystrophin (dystroΔ1168) that lacks 1,168 amino acids at the C-terminus, encoded by exons 52 to 79. Exon 51 skipping in this model generates an mRNA isoform that restores the reading frame, leading to production of an internally deleted dystrophin (dystroΔ117) that lacks only 117 amino acids encoded by the two missing exons (51 and 52). Using a monoclonal antibody, we detected expression of dystroΔ117 in tissues collected on day 30, but not dystroΔ1168, though the epitope is located between residues 1181 and 1388 encoded by exons 26 to 30 (27). While both decoy-less ASOs had no effect on protein expression, the two 5D ASOs increased dystroΔ117 expression in both skeletal and cardiac tissues, with expression levels reaching 32% (Etep-L8c) and 35% (000A-L8c) in DI and 4% (Etep-L8c) and 7% (000A-L8c) in heart, compared to full-length dystrophin expressed in the same tissues of wild-type mice (Fig. 4*C*).

Both 5D ASOs displayed superior potency in restoring dystrophin expression in mice, highlighting their therapeutic potential. Particularly, 000A-L8c, which is shorter, elicited overall higher exon skipping in mouse tissues than Etep-L8c. We next assessed the relative efficacy between 000A-L8c and MOE-Etep. Male huΔ52 mice were injected with 3, 10, 30, or 100 mg/kg 000A-L8c, or 100 mg/kg MOE-Etep via the tail vein, once a week for five consecutive weeks. Mice were sacrificed 30 d after the last injection (Fig. 4*D*). We measured the levels of the exon 51-skipped transcript and dystroΔ117 by qRT-PCR and immunoblotting, respectively. We observed a dose-dependent increase in exon skipping at both mRNA and protein levels across all tested tissues of mice treated with 000A-L8c. Remarkably, 3 mg/kg 000A-L8c showed a comparable effect to 100 mg/kg MOE-Etep in both skeletal and cardiac muscles, indicating a ~30-fold increase in potency. At the same dose of 100 mg/kg, 000A-L8c elicited a more robust increase in both exon 51-skipped transcript and dystroΔ117 levels than MOE-Etep (Fig. 4 *E* and *F*).

We next performed grip-strength and rotarod tests on days 0 and 58 to evaluate whether ASO treatment improved motor function of huΔ52 mice. Treatment with 000A-L8c at 30 and 100 mg/kg significantly improved the four-limb grip strength, compared to both saline and MOE-Etep groups (Fig. 4*G* and *SI Appendix*, Table S1), while the rotarod test revealed a trend of increasing mean values of latency as the dose increased, although these increases were not significant compared to the saline control, which we attribute to the large intragroup variability (*SI Appendix*, Fig. S8*A*).

Serum creatine kinase (CK) is commonly used as a diagnostic biomarker for DMD; its levels are consistently elevated in both patients and animal models (28, 29). We measured mouse CK in serum samples collected on day 60. As shown in Fig. 4*H*, 000A-L8c markedly reduced serum CK levels from 6,715 U/L (saline) to 2,883 U/L (10 mg/kg), 1,995 U/L (30 mg/kg) and 1,041 U/L (100 mg/kg), respectively, whereas MOE-Etep treatment resulted in no change.

We performed hematoxylin & eosin (H&E) staining to detect morphological changes in DI, QU, TR, and heart. We assessed the severity of muscle injury using a scoring system based on grading several histopathological parameters, including myofiber necrosis, fibrosis, and the presence of inflammatory and fatty cells. We observed myofiber injury and inflammatory cell infiltration in all tissues, which is consistent with the pathological changes detected in other murine DMD models (26, 30). 000A-L8c treatment at 100 mg/kg improved muscle status, compared to saline-treated huΔ52 mice, while MOE-Etep at the same dose did not result in any significant benefit (*SI Appendix*, Fig. S8*B* and Table S2).

We also tested 000A-L8c in male Δ52 mice. Subcutaneous administration with 000A-L8c, despite having one mismatch to the target mouse sequence, led to robust exon 51 skipping, marked dystrophin restoration and phenotypic improvement, including in both grip strength and rotarod performance (*SI Appendix*, Fig. S9 and Table S3). At 300 mg/kg, the effect persisted over 4 mo in skeletal muscle (*SI Appendix*, Fig. S10).

All three types of genetically modified mice showed no signs of toxicity after ASO treatment. To further evaluate the safety of the two 5D ASOs, we intravenously injected male wild-type C57BL/6N mice with 100 mg/kg 000A-L8c or Etep-L8c once a week for five consecutive weeks. We collected blood and urine samples on day 30 for biochemical analysis and urinalysis, respectively (*SI Appendix*, Tables S4 and S5). For the 14 serum and 12 urine parameters tested, the results were largely unremarkable, with the exception of serum creatinine, which showed a <threefold increase. We harvested liver and kidney tissues on day 30 and examined them with H&E staining, which revealed only slight hypertrophy of Kupffer cells, with basophilic vacuolation, and minor occurrence of basophilic granules in epithelial cells of the renal tubules (*SI Appendix*, Table S6). These are typical nonadverse effects of ASO accumulation in the two tissues (31, 32). No other abnormalities were observed.

**Fig. 5.**
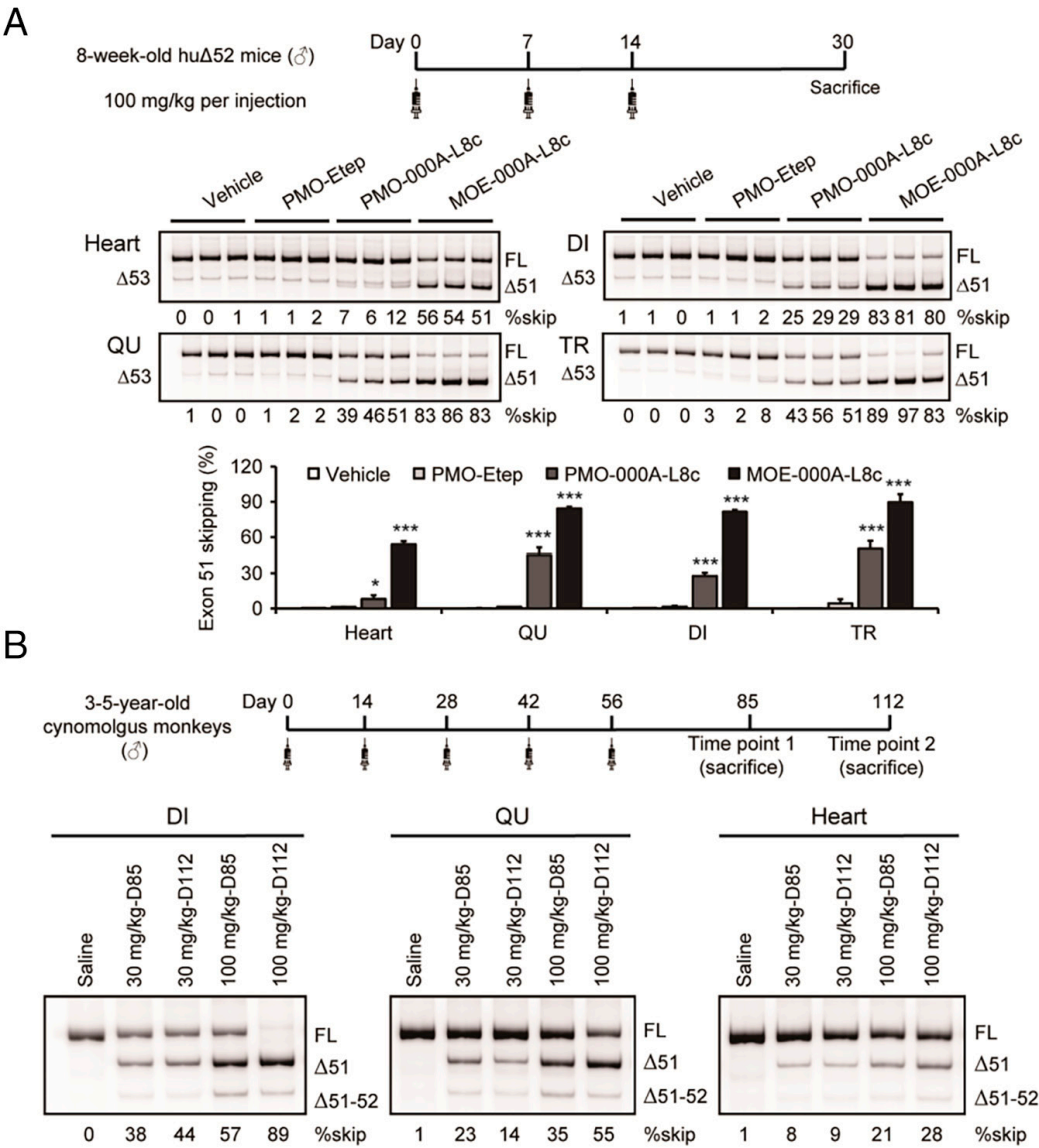
Comparison of MOE-version and PMO-version of 000A-L8c in huΔ52 mice, and analysis of 000A-L8c in cynomolgus monkeys. (*A*) Comparison of the efficacy of MOE and PMO versions of 000A-L8c in huΔ52 mice. Male mice 8-wk-old were subcutaneously administered with each ASO at 100 mg/kg on days 0, 7, and 14, and the FDA-approved eteplirsen (PMO-Etep) was used as a positive control. Mice were killed humanely 1 mo after the initial injection. and four muscle tissues (QU, DI, TR, and heart) were collected. Exon 51 skipping in tissue samples was analyzed by sqRT-PCR, with quantitation on the right (*n* = 3). *P < 0.05, ***P < 0.001, compared with PMO-Etep. ^##^*P* < 0.01, ^###^*P* < 0.001, compared with PMO-000A-L8c. (*B*) Analysis of exon 51 skipping in three tissues (DI, QU, and heart) of cynomolgus monkeys treated with MOE/PS-modified 000A-8Lc by sqRT-PCR. Male monkeys 3 to 5 y old were administered 000A-8Lc biweekly via intravenous infusion at 30 or 100 mg/kg on days 0, 14, 28, 42, and 56, and killed humanely on day 85 or 112. Only one monkey was used for each time point and dose.

Both 000A-L8c and MOE-Etep used in the above animal studies are MOE/PS modified, while the actual Etep is a PMO. We therefore compared MOE/PS and PMO versions of 000A-L8c in male huΔ52 mice, with the PMO-version Etep (PMO-Etep) as a positive control. We subcutaneously injected 8-wk-old mice weekly with each ASO at 100 mg/kg for three consecutive weeks and sacrificed them 2 wk after the last injection. We measured exon 51 skipping changes in heart, QU, DI, and TR tissues by sqRT-PCR. As shown in Fig. 5*A*, treatment with MOE-version 000A-L8c increased exon skipping to over 50% in heart and over 80% in all skeletal muscles, compared to ~8% (heart) and 25 to 51% (skeletal muscles) in mice treated with PMO-version 000A-L8c. PMO-Etep had little or negligible effect on exon skipping in all tissues.

### Efficacy and Safety Analysis of 000A-L8c in Cynomolgus Monkeys.

Due to close genetic and physiological similarities to humans, nonhuman primates play an important role in drug development. We used cynomolgus monkeys, which have the identical exon 51 ASO-target sequence as humans, to test the efficacy and safety of MOE/PS-modified 000A-L8c. We administered the ASO biweekly by intravenous infusion five times at 30 or 100 mg/kg, and the animals (7 in total) were sacrificed 4 or 8 wk after the last ASO infusion. Although we used only one monkey for each time point of each dose, the dose-dependent effect of 000A-L8c on exon 51 skipping in both skeletal muscle and heart tissues was apparent (Fig. 5*B*).

We collected blood and urine samples of treated monkeys at different time points and performed routine blood tests, coagulation function tests, blood biochemical tests, immunotoxicology tests, and urinalysis. Urinalysis results were within normal limits for all monkeys treated with 000A-L8c. At 30 mg/kg, among 49 tested blood parameters, only the C-reactive protein (CRP) concentration was moderately higher than normal at one time point for each monkey, and at 100 mg/kg, only increases in CRP and fibrinogen, as well decreases in C3 and lymphocytes at some time points were detected (*SI Appendix*, Table S7).

## Discussion

Since the concept of ASO-mediated exon-skipping therapeutics was proposed three decades ago (33), progress has been slow, reflecting a requirement to improve ASO design and/or delivery for more efficient exon skipping. Here, we developed a simple but effective method, 5D-ASO, to promote efficient exon skipping using bipartite ASOs with a short 5′ss consensus sequence as a 5′ or, in some cases, 3′ tail. The targeting portion was either empirically designed or obtained from a systematic screening procedure and serves two roles: first, by itself it promotes exon skipping through binding to a splicing enhancer element to occlude access of a splicing activator to the element, or by disrupting a splicing-favorable RNA structure; second, it acts as a carrier to bring the decoy to the vicinity of a 5′ss of interest, where the decoy presumably traps U1 snRNP to further repress splicing at that 5′ss. We confirmed 5D-ASO’s wide applicability with diverse genes and exons. Moreover, using *DMD* exon 51 as an example, we demonstrated that this method is robustly effective in vivo.

The efficiency of a U1 decoy depends heavily on its placement. Whether placement at the 5′ or 3′ end of an ASO results in optimal exon-skipping activity is context-dependent, although in most of our test cases, placement at the 5′ end was best, which can be explained by the decoy in this orientation being closer along the pre-mRNA to the 5′ss of interest. However, exceptions such as ASO1938-R11 being stronger than ASO1938-L11 for *SMN1/2* exon 7 skipping and (CUG)7-R8d being the most potent for *ATXN3* exon 10 suggest that other mechanisms are potentially involved, e.g., spatial proximity in three dimensions. The length is also a key factor in determining the efficiency of a decoy. Although, theoretically, an 11-nt decoy that fully hybridizes to the single-stranded 5′ end of U1 snRNA should have maximal binding affinity, we found that the optimal lengths often were 7 to 8 nt. In addition, for decoys with the same length, a shift by a single nucleotide sometimes resulted in marked differences in efficiency. These observations likely reflect the complexity of interactions between local sequences in the overall quaternary structure. An important question is why some 5D ASOs (e.g., Etep-L8c) gained a massive boost in exon skipping over their decoy-less counterparts, while others had more limited or no effects. The exon and flanking intron sequences in these extreme cases should be further investigated to elucidate the relevant features therein.

Direct interaction between the decoy tail and U1 snRNA is the core principle of the 5D-ASO method, which means any chemical modifications to improve pharmacology that are compatible with base pairing can be used, in contrast to approaches that involve recruitment of RNA-binding proteins, whose binding may not tolerate certain chemical modifications. Indeed, using L11 attached to Etep, we showed that morpholino and OMe modifications were similarly potent as MOE in promoting *DMD* exon 51 skipping (Fig. 2).

U1 snRNA undergoes 5′ cap hypermethylation and assembly into U1 snRNP in the cytoplasm, before being imported into the nucleus for final maturation and for its splicing and telescripting functions (34). As a result, base pairing between the decoy and U1 snRNA may enhance nuclear import of 5D ASOs, which could be another mechanism contributing to their enhanced efficacy. The long duration of action of 5D ASOs observed in mice might reflect debranching and degradation of the excised lariat with the skipped exon liberating the ASO for recycling. Despite sharing similar distribution profiles across blood, heart, and skeletal muscle, pharmacokinetic evaluation revealed prolonged tissue retention of Etep-L8c compared to a previously described 20-nt *Dmd* exon 23-skipping OMe/PS ASO, which is consistent with their differential exon-skipping effects (35). Therefore, alongside its superior potency, the enhanced pharmacokinetic profile—likely driven by a combination of its length, distinct chemical modifications, and primary sequence, including the tail—contributes to the sustained exon-skipping efficacy of Etep-L8c, although a minor contribution from differential metabolic stability between the two exon-skipped transcripts cannot be ruled out.

Gendron et al. previously designed bifunctional ASOs with a linear or branched 3′ tail comprising one or two human *HBB* 5′ss (GUUGGUAUG) to redirect *Bcl-x* splicing in favor of the pro-apoptotic Bcl-xS isoform (36). These ASOs worked in cell-free splicing assays, although the 5′ss sequence used in the study deviates substantially from the consensus. Bartys et al. tested 1 to 3 copies of the consensus 5′ss (CAGGUAAGU) as a regulatory portion (RP) to manipulate *PKM* splicing (37) and concluded that three copies were required to cause PKM2/PKM1 ratio changes; however, the ratio decreased only ~twofold in HeLa cells transfected with 250 nM of the 27-nt-tailed ASO, compared to its tail-less counterpart. In contrast, we achieved >sevenfold decrease in the ratio, using a tail as short as 8-nt, with the same transfection reagent and at a fivefold lower concentration (50 nM) (*SI Appendix*, Fig. S4). We note that the tail was added to the 3′ end of the ASO in that study, and based on our data, the effect should be stronger when it is added to the 5′ end.

Exon skipping has been extensively pursued as an approach to treat DMD, and it is applicable to 55% of all DMD-causing mutations, including 80% of DMD-causing deletions (17). However, all four conditionally approved exon-skipping PMOs have limited efficacy, resulting in marginal clinical benefit (38). To improve their efficacy, current efforts are mainly focused on chemistry and delivery improvements. Here we show that optimization of the ASO sequence itself—including the decoy portion—enhances efficacy. For the MOE-version 000A-L8c, we observed a robust improvement in exon skipping, which was cross-validated using both sqRT-PCR and qRT-PCR. To ensure technical accuracy, multiple RT-PCR assays flanking the ASO target region confirmed that the ASO does not interfere with reverse transcription, even when transfected at a high concentration (25 nM) (*SI Appendix*, Fig. S11). Certain in vivo findings were further validated by droplet digital PCR (*SI Appendix*, Fig. S12) and western blotting. Notably, the MOE-version 000A-8Lc outperformed its PMO counterpart in vivo, reflecting its longer half-life in the circulation, which is consistent with our previous study of *SMN2*-targeted ASOs (39). Although further histopathological evaluations—such as quantifying muscle fiber cross-sectional area in ASO-treated mice—are warranted, these collective data demonstrate a substantial increase in efficacy for MOE-version 000A-L8c compared to eteplirsen.

The decoy in the 5D ASOs, if present in excessive amounts, may sequester U1 snRNP, leading to global splicing defects and PCPA. However, U1 is highly abundant, and we demonstrated that there is a safe concentration window in which sufficient exon skipping is achieved, with negligible gene-expression perturbations. There are inconsistent reports regarding U1 snRNP and the risk of cancer. Oh et al. uncovered an inhibitory role of U1 snRNP in cancer cell migration and invasion in vitro (24). On the contrary, Cheng et al. found that overexpression of U1 snRNA upregulated some key cancer genes in vitro (40). Mikami et al. reported that in patients with recurrent high-risk prostate cancer, the expression level of the major U1 snRNA genes *RNU1-1/1-2* was significantly higher than in patients without recurrence (41). Our three in vitro assays in cells treated with Etep-L8c showed no increase in proliferation (*SI Appendix*, Fig. S13).

One caveat for systemic administration of ASOs to treat muscle diseases is the potential for liver and kidney toxicity, because these are the organs where ASOs tend to accumulate at the highest concentrations. However, mouse tissue staining, as well as serum and urine analyses in both mice and monkeys showed an overall safe profile. These data strongly support further clinical investigation of the lead 5D ASO as a potential drug to treat DMD rescuable by skipping of exon 51, and of similarly designed 5D ASOs to skip other exons in the appropriate subsets of DMD patients.

In summary, we have developed an efficient ASO-mediated exon-skipping platform with broad applicability. In the future, the in vivo performance of 5D ASOs may be further enhanced with novel chemistries that have better pharmacodynamic and pharmacokinetic properties, approaches that promote ASO cellular uptake or endosomal release, and tissue-specific delivery systems. For example, an antibody-oligonucleotide-conjugate (AOC) strategy using anti-TfR1 antibody linked to eteplirsen (42), or to an exon 44-skipping PMO, is under clinical evaluation (43, 44). These AOC products not only improve muscle uptake of the PMOs, but also extend their half-life in blood. We expect that a similar AOC strategy would likewise boost the efficacy of 000A-L8c.

## Materials and Methods

### Oligonucleotides, Cell Culture, and Transfections.

MOE/PS- and OMe/PS-modified ASOs with all 5-methyl cytosines were purchased from Biosyntech (Suzhou, Jiangsu, China) and General Biol (Chuzhou, Anhui, China); PMOs were from Apextide (Hangzhou, Zhejiang, China). Oligonucleotide sequences are listed in *SI Appendix*, Table S8. All cell types were cultured in Dulbecco’s modified Eagle’s medium (Life Technologies, Carlsbad, CA), supplemented with 10% (v/v) fetal bovine serum (FBS), 100 U/mL penicillin, and 100 μg/mL streptomycin at 37 °C in a humidified 5% CO_2_ atmosphere. Each ASO (other than PMOs) was transfected into 4 × 10^5^ cells with 500 ng of the *SMN1* minigene plasmid, or without any plasmid, using Lipofectamine 2000 (Life Technologies) in 12-well plates. 30 h posttransfection, cells were harvested for splicing analysis. PMOs were gymnotically delivered to cells for 30 h.

### sqRT-PCR and qRT-PCR.

Total RNA was isolated from cultured cells using TransZol Up reagent (TransGen Biotech, Beijing, China) and 2 μg of each RNA sample was reverse-transcribed into cDNA with TransScript® IV Reverse Transcriptase (TransGen Biotech) in a 20-μL reaction. For splicing or PCPA analysis, one of the primers was labeled with Cy5, and Cy5-labeled PCR products were separated on 6% native polyacrylamide gels, followed by fluorescence imaging using G-box (Syngene, Cambridge, UK). The extent of exon skipping was calculated as a percentage of the total spliced mRNA (%skip). qRT-PCR was performed using Applied Biosystems QuantStudio 3 System (Thermo Fisher Scientific, Waltham, MA) using PerfectStart Green qPCR SuperMix (TransGen Biotech). For mRNA level analysis of *FOS*, *MYC*, *RB1,* and *ATR*, *GAPDH* was used as an internal control. Primer sequences are listed in *SI Appendix*, Table S9.

### Animals.

Animal experiments involving mice were performed at Cyagen (Suzhou, Jiangsu, China) and Nanjing Normal University, and studies with cynomolgus monkeys were conducted at SAIFU Laboratories (Beijing, China); all protocols were approved by the Ethics Committees of the respective organizations.

The huΔ52 mouse model (*Dmd*^huΔ52/Y^ or *Dmd*^huΔ52/huΔ52^) was generated by deletion of part of the humanized genomic region comprising the last 550 nt of intron 51, the 118-nt exon 52 and the first 250 nt of intron 52 in a humanized mouse strain termed B6-hDMD(E49-53) (strain number: C001775, Cyagen, Suzhou, Jiangsu, China), in which the genomic DNA fragment of the mouse *Dmd* gene from the latter part of intron 48 to the front part of intron 53 was replaced by the corresponding sequence of the human *DMD* gene. Only male animals were used. ASOs were administered either subcutaneously or intravenously at the indicated doses. Mice were sacrificed using cervical dislocation, while cynomolgus monkeys were killed humanely after anesthesia, and tissues were snap-frozen in liquid N_2_ and kept at −80 °C.

Grip strength was measured using a grip-strength meter (BIO-GS4, Bioseb, Pinellas Park, FL). Mice were allowed to grasp the bar and/or grid of the meter with all four paws, and pulled back horizontally. Five trials at 1-min intervals for each mouse were performed, and the best three performances were recorded and normalized to body weight.

### Western Blotting.

Mouse tissues were ground in liquid N_2_ with mortar and pestle, and lysed with an equal volume of 2× SDS-PAGE loading buffer (Beyotime Biotechnology, Shanghai, China). Protein samples were separated by 6% SDS-PAGE and transferred to nitrocellulose membranes (Pall Life Sciences, Port Washington, NY) for 4 h. Blots were then incubated with an anti-dystrophin monoclonal antibody NCL-DYS1 (Leica, Wetzlar, Germany), or an anti-β-actin or anti-desmin polyclonal antibody (Proteintech, Wuhan, Hubei, China), followed by incubation with IRDye 680RD goat anti-mouse or IRDye 800CW goat anti-rabbit secondary antibody (LI-COR Biosciences, Lincoln, NE). Signals were detected with an Odyssey Infrared Imaging System (LI-COR Biosciences).

### Serum CK Measurement.

Mouse blood was collected from the orbital sinus of mice anesthetized with isoflurane, kept at room temperature for 2 h, and then serum was separated by centrifugation at 3,000 rpm for 15 min at 4 °C and stored at −80 °C for further analysis. A CK assay kit (Rayto, Shenzhen, Guangdong, China) was used according to the manufacturer’s instructions, and the absorption at 340 nm was measured in a Chemray 800 Auto Chemistry Analyzer (Rayto) to calculate serum CK activity.

### Statistical Analysis.

Experimental data were analyzed by two-tailed Student’s *t* tests with SPSS 16.0 (IBM, Armonk, NY) and presented as mean ± SD. A *P* value of less than 0.05 was considered significant.

## Supplementary Material

Appendix 01 (PDF)

## Data Availability

Study data are included in the article and/or *SI Appendix*.
