# Supplementary material for: An antisense method for efficient exon skipping and its application to Duchenne muscular dystrophy
Source: Proc Natl Acad Sci U S A. 2026 Aug 10;123(33):e2606494123. doi: 10.1073/pnas.2606494123 (PMC13486594; doi:10.1073/pnas.2606494123)
Supplement: Supplementary file 1 [file pnas.2606494123.sapp.pdf]

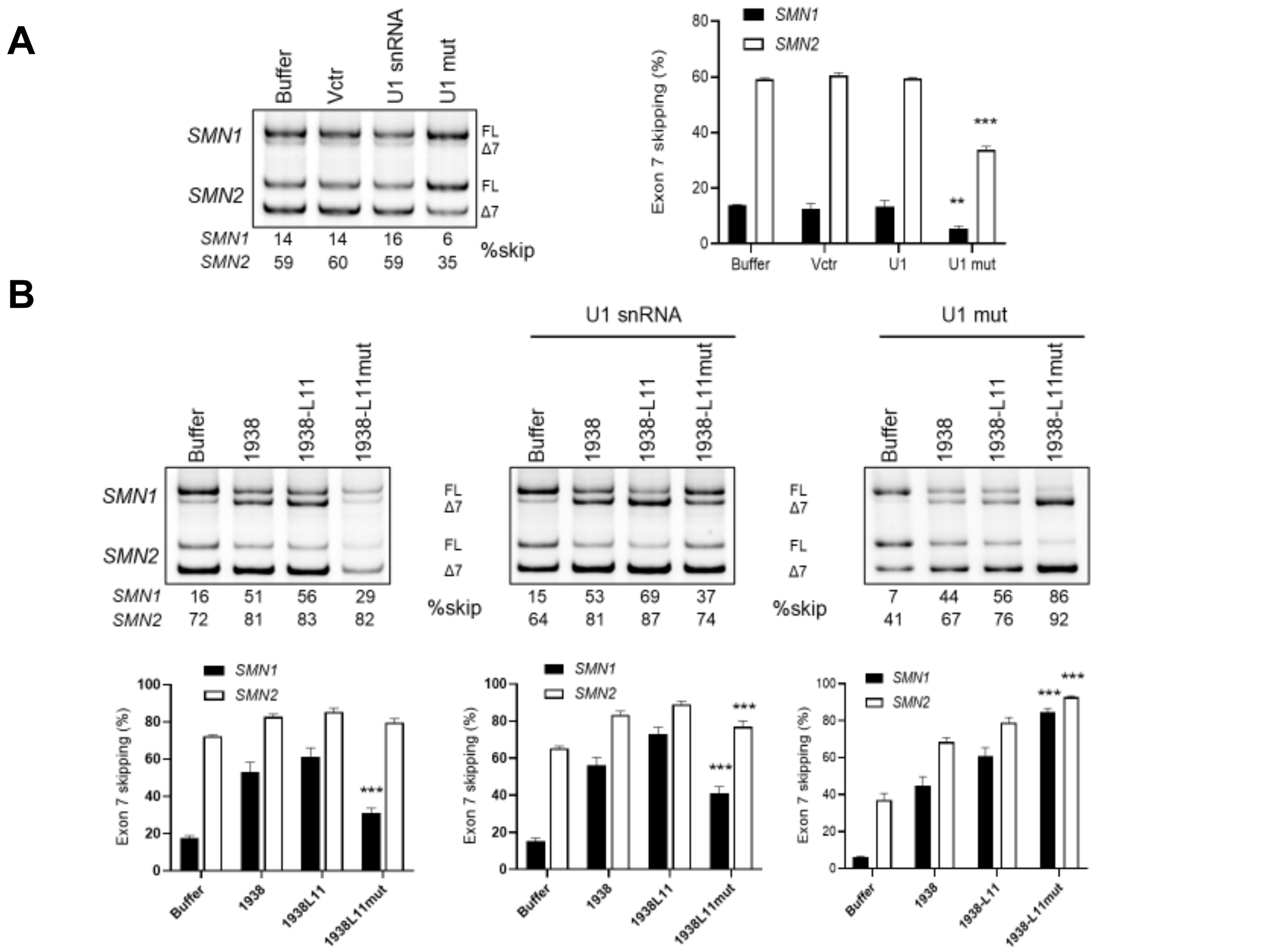


**Supplementary Fig. 1 | U1 snRNA suppressor assay confirms that the decoy used in bipartite ASOs acts through binding to U1 snRNA. (*A*).** A U1 snRNA mutant (U mut) markedly promoted *SMN1/2* exon 7 splicing. The construct expressing U1 snRNA was generated with vector pLenti-U6, and the mutant construct expressing U1 mut was generated by mutagenesis, with the 11-nt 5′-end sequence of U1 snRNA changed to 5′-AGACTTACTCC-3′, which is perfectly complementary to the 11-nt sequence at the 5′ss of *SMN1/2* exon 7. HEK293 cells were transfected with 500 ng of plasmid expressing the wild-type U1 snRNA or U1 mut. Empty vector (Vctr) and buffer were used as negative controls. Cells were collected 24 h post-transfection and exon 7 splicing of the endogenous *SMN1/2* genes was analyzed using sqRT-PCR. The percentage of exon 7 skipping (% skip) of each sample is shown below the gel image, and the quantitative data of % skip ($n$ = 3) are shown on the right. Note that at 500 ng per well, U1 snRNA failed to promote exon 7 splicing. ** $P < 0.05$, *** $P < 0.001$, compared with all other three groups. **(*B*).** HEK293 cells were transfected with 5 nM ASO1938, 1938-L11, or 1938-L11mut, or co-transfected with each ASO (5 nM) and a vector (500 ng) expressing U1 snRNA or U1 mut. ASO1938-L11mut represents ASO1938 extended at the 5′ end with a decoy targeting U1 mut (L11mut; seq: 5′-GGAGTAAGTCT-3′). Splicing was analyzed as in **(*A*)**. L11mut markedly enhanced ASO1938-mediated *SMN1/2* exon 7 skipping, compared with L11 in cells expressing U1 mut, but exerted no or inhibitory effects in vector-free cells or cells over-expressing U1 snRNA. These data demonstrate that the decoy operates, mainly if not entirely, via a U1 snRNA-dependent mechanism. *** $P < 0.001$, compared with 1938-L11.

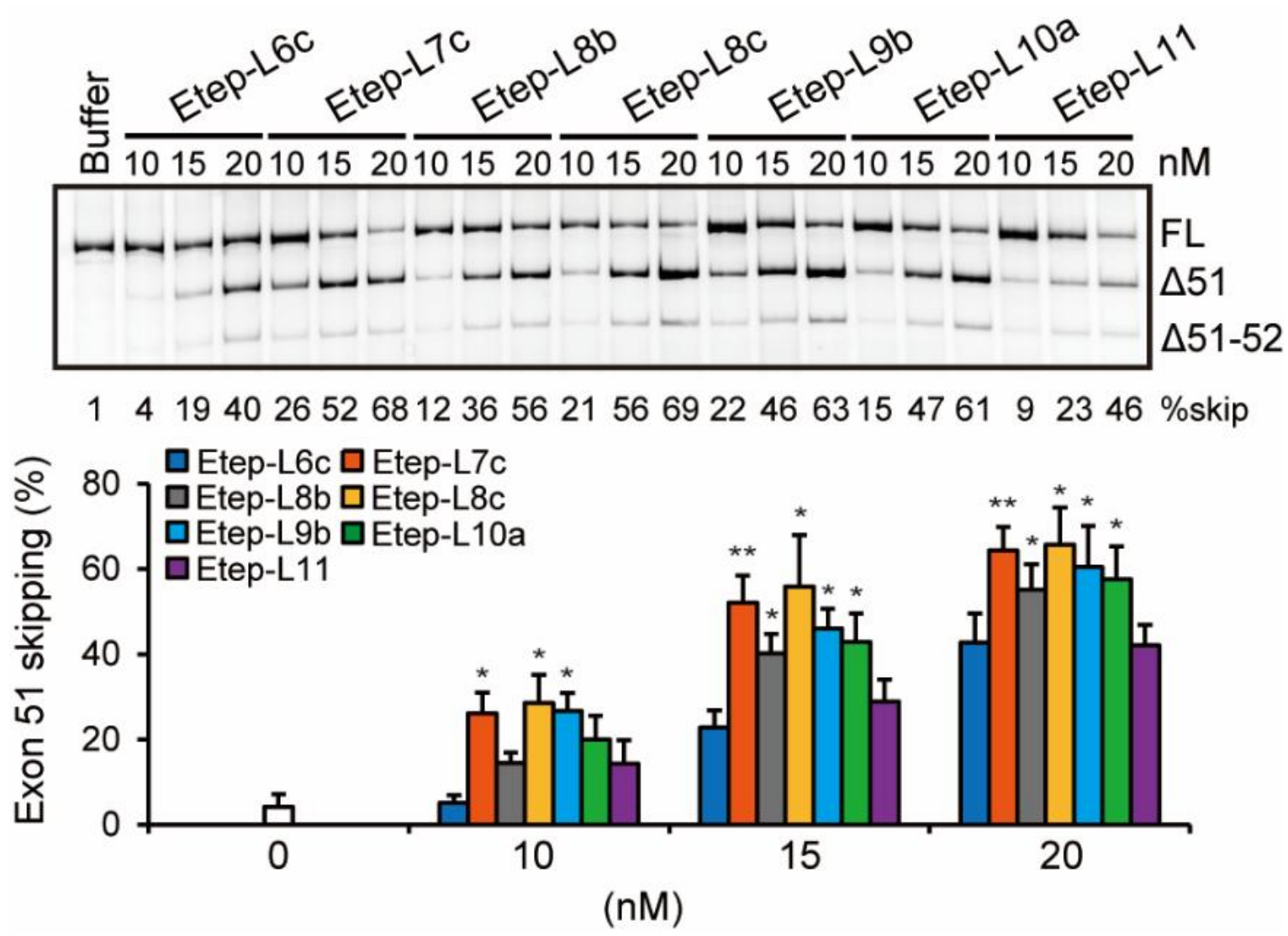


**Supplementary Fig. 2 | Comparison of six top decoys attached to the 5′ end of Etep, at three concentrations, on enhancing *DMD* exon 51 skipping.** The exon-skipping effects of Etep-L6c, L7c, L8b, L8c, L9b, L10a and L11 were compared. Each ASO was transfected at three concentrations (10, 15, and 20 nM) into RD cells. RNA samples were analyzed by Cy5-labeled sqRT-PCR. A representative image and quantitation of exon 51 skipping (% excl) from three independent experiments are shown. Buffer, sham transfection (without ASO); FL: full-length; Δ51: exon 51-skipped; Δ51-52: both exons (51 and 52)-skipped. * $P < 0.05$, ** $P < 0.01$, all compared with Etep-L11 at the same concentrations.

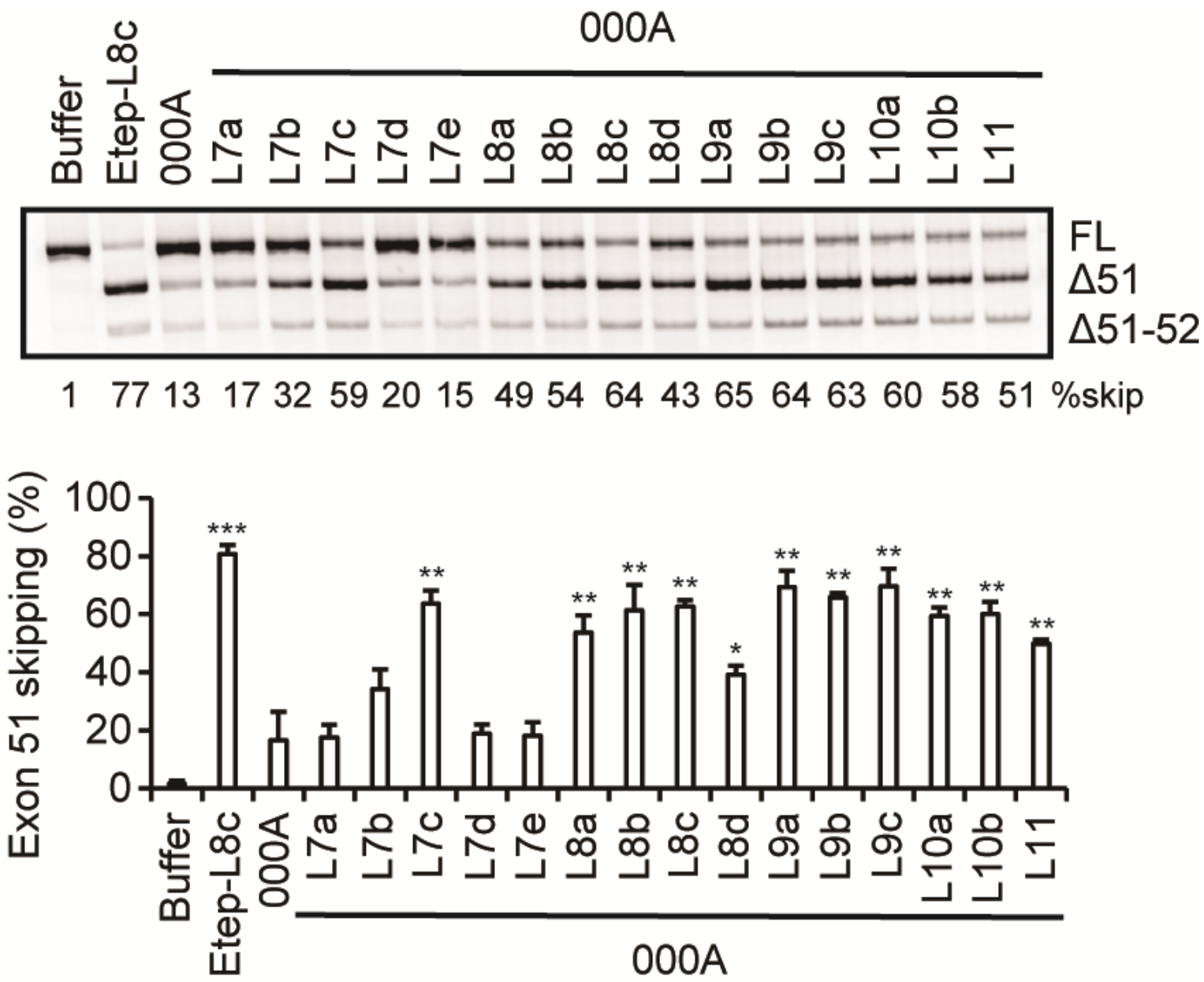


**Supplementary Fig. 3 | Comparison of 7-11 nt decoys attached to the 5′ end of 000A on enhancing *DMD* exon 51 skipping.** All 15 decoys with lengths of 7-11 nt attached to the 5′ end (L) of 000A were compared for their effects on *DMD* exon 51 skipping. 25 nM of each ASO was transfected into RD cells. Etep-L8c was used as positive control. RNA samples were analyzed by sqRT-PCR. A representative image and quantitation of exon 51 skipping (% excl, $n = 3$) are shown. * $P < 0.05$, ** $P < 0.01$, *** $P < 0.001$, all compared with 000A.

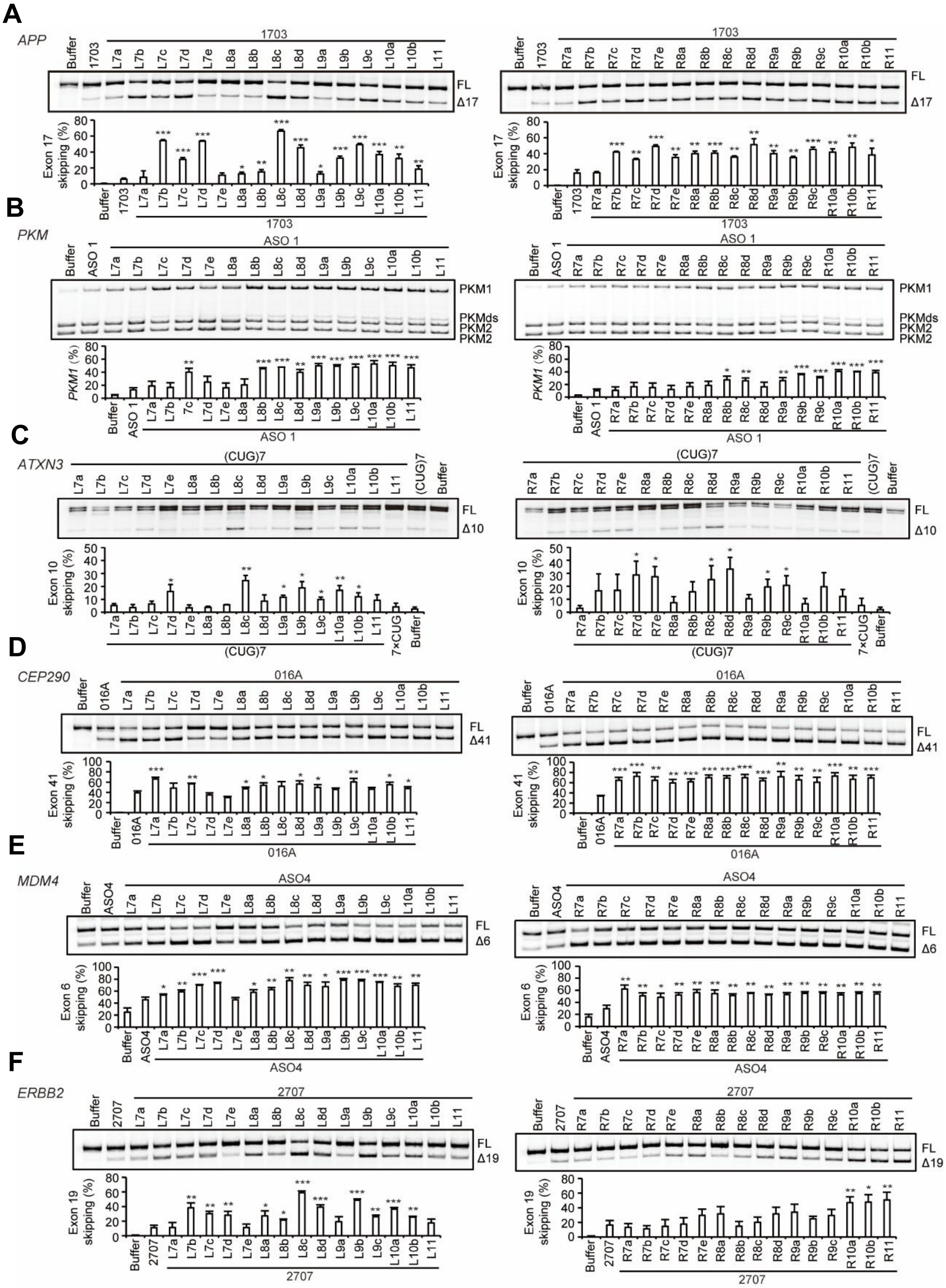
A
APP
Buffer
1703
L7a L7b L7c L7d L7e L8a L8b L8c L8d L9a L9b L9c L10a L10b L11
R7a R7b R7c R7d R7e R8a R8b R8c R8d R9a R9b R9c R10a R10b R11
FL
Δ17
Exon 17 skipping (%)
B
PKM
ASO 1
PKM1
PKMds
PKM2
PKM2
PKM1 (%)
C
ATXN3
(CUG)7
7×CUG
FL
Δ10
Exon 10 skipping (%)
D
CEP290
016A
FL
Δ41
Exon 41 skipping (%)
E
MDM4
ASO4
FL
Δ6
Exon 6 skipping (%)
F
ERBB2
2707
FL
Δ19
Exon 19 skipping (%)

**Supplementary Fig. 4 | The effect of decoys on enhancing exon skipping of six additional genes.** (***A***)**.** Effects of all 15 decoys with lengths of 7-11 nt on *APP* exon 17 skipping when attached to the 5′ (L) or 3′ (R) end of 1703. 50 nM of each ASO was transfected into HEK293 cells. (***B***)**.** Effects of these decoys on *PKM* exon 9 inclusion and exon 10 exclusion when attached to the 5′ or 3′ end of ASO1. 50 nM of each ASO was transfected into RD cells. Digestion of PCR products with PstI was performed to distinguish PKM1 (restriction site absent) from PKM2 (one site present). PKMds: both-exons (9 and 10) are skipped. (***C***)**.** Effects of these decoys on *ATXN3* exon 10 skipping when attached to the 5′ or 3′ end of (CUG)7. 40 nM of each ASO was transfected into A549 cells. (***D***)**.** Effects of all 15 decoys with lengths of 7-11 nt on *CEP290* exon 41 skipping when attached to the 5′ (L) or 3′ (R) end of 016A. 50 nM of each ASO was transfected into HEK293 cells. (***E***)**.** Effects of these decoys on *MDM4* exon 6 skipping when attached to the 5′ or 3′ end of ASO4. 50 nM of each ASO was transfected into HEK293 cells. (***F***)**.** Effects of these decoys on *ERBB2* exon 19 skipping when attached to the 5′ or 3′ end of 2707. 50 nM of each ASO was transfected into HeLa cells. All RNA samples were analyzed by sqRT-PCR. 1703, ASO1, (CUG)7, ASO4 and 2707 were based on previous studies (1-5); 016A was obtained from an ASO screen. A representative image and quantitation of exon skipping (% excl, $n = 3$) are shown. * $P < 0.05$, ** $P < 0.01$, *** $P < 0.001$, all compared with the corresponding decoy-less ASOs.

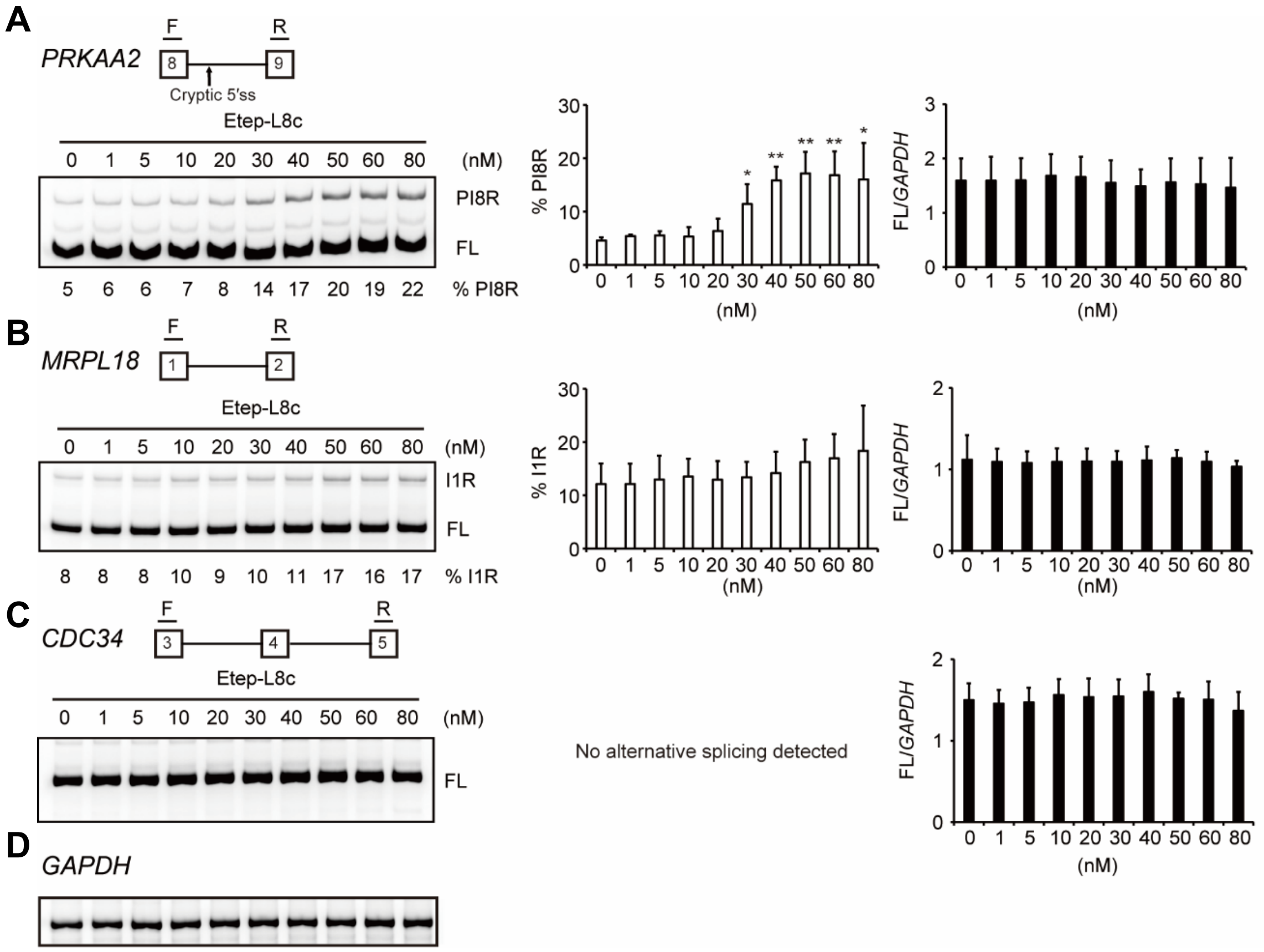


**Supplementary Fig. 5 | Examination of Etep-L8c's effects on three previously reported alternative splicing events.** The same RNA samples as in **Fig. 4*A*** were used for sqRT-PCR to examine the effects of Etep-L8c on a partial intron retention event of *PRKAA2* (***A***) due to an alternative 5′ss located in intron 8*,* intron 1 retention of *MRPL18* (***B***), and alternative splicing of *CDC34* (***C***) due to the presence of an alternative 5′ss and an alternative 3′ss in intron 4. *GAPDH* (***D***) was used as internal control. PI8R: partial intron 8 retention; I1R: intron 1 retention; F: forward primer; R: reverse primer. Quantitation of each alternatively spliced isoform as a percentage of total transcripts and the ratio of each normal FL transcript to *GAPDH* are shown ($n$ = 3). * $P < 0.05$, ** $P < 0.01$, all compared with 0 nM.

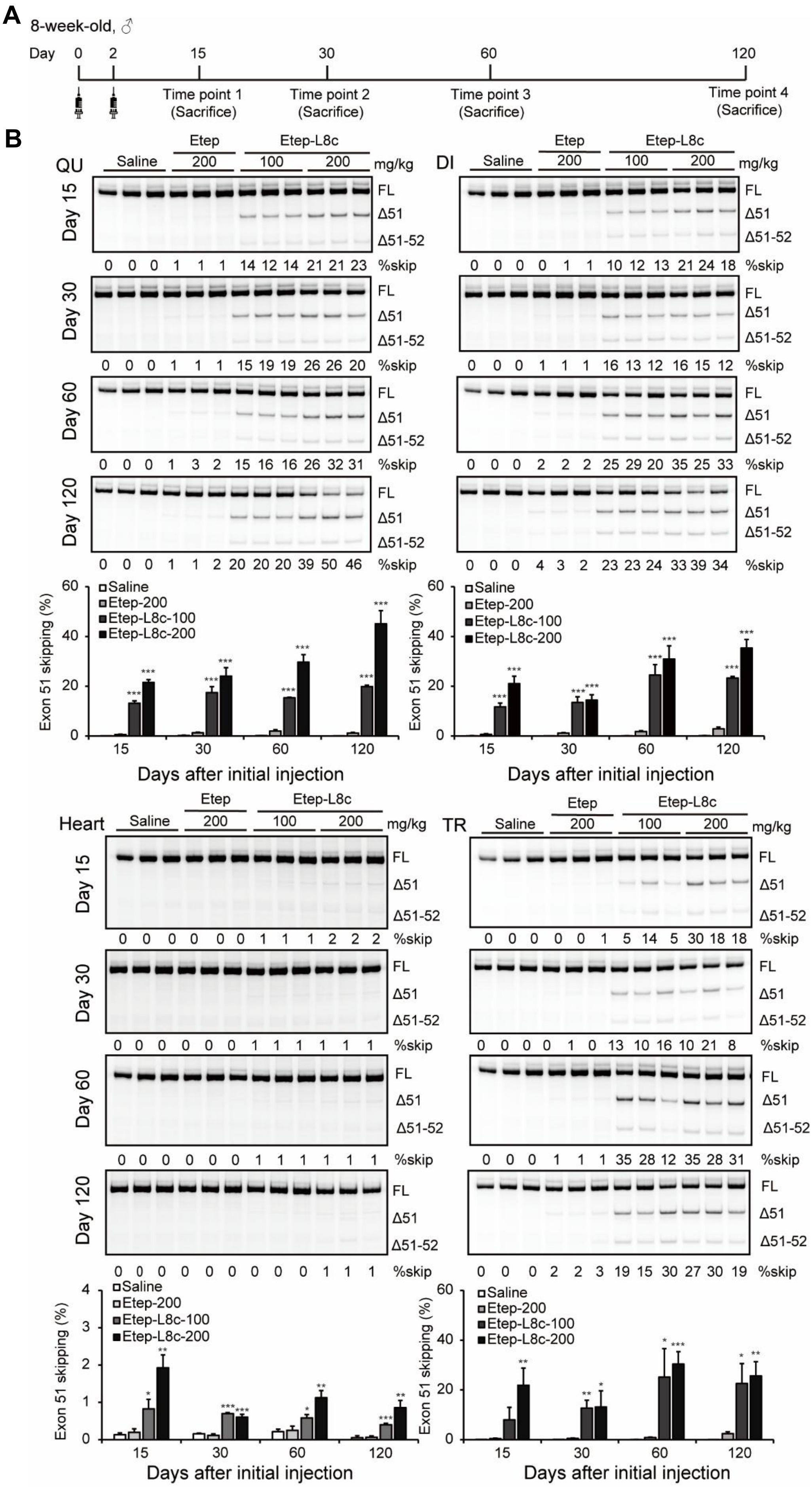

A
8-week-old, ♂
Day 0 2 15 30 60 120
Time point 1 (Sacrifice)
Time point 2 (Sacrifice)
Time point 3 (Sacrifice)
Time point 4 (Sacrifice)
B
QU
Saline
Etep 200
Etep-L8c 100 200
mg/kg
Day 15
FL
Δ51
Δ51-52
0 0 0 1 1 1 14 12 14 21 21 23 %skip
Day 30
0 0 0 1 1 1 15 19 19 26 26 20 %skip
Day 60
0 0 0 1 3 2 15 16 16 26 32 31 %skip
Day 120
0 0 0 1 1 2 20 20 20 39 50 46 %skip
Saline
Etep-200
Etep-L8c-100
Etep-L8c-200
Exon 51 skipping (%)
Days after initial injection
DI
0 0 0 0 1 1 10 12 13 21 24 18 %skip
0 0 0 1 1 1 16 13 12 16 15 12 %skip
0 0 0 2 2 2 25 29 20 35 25 33 %skip
0 0 0 4 3 2 23 23 24 33 39 34 %skip
Heart
0 0 0 0 0 0 1 1 1 2 2 2 %skip
0 0 0 0 0 0 1 1 1 1 1 1 %skip
0 0 0 0 0 0 1 1 1 1 1 1 %skip
0 0 0 0 0 0 0 0 0 1 1 1 %skip
TR
0 0 0 0 0 1 5 14 5 30 18 18 %skip
0 0 0 0 1 0 13 10 16 10 21 8 %skip
0 0 0 1 1 1 35 28 12 35 28 31 %skip
0 0 0 2 2 3 19 15 30 27 30 19 %skip

**Supplementary Fig. 6 | The effect of Etep-L8c on exon 51 skipping of a humanized *Dmd* gene in huEx51 mice.** Healthy huEx51 mice (*Dmd*$^{hu/Y}$ or *Dmd*$^{hu/hu}$; hu, humanized allele) were generated by GemPharmatech (Nanjing, Jiangsu, China) in the C57BL/6J background, in which a 10,766-nt genomic DNA fragment of the mouse *Dmd* gene, including the last 9,053 nt of intron 50, the 233-nt exon 51, and the first 1,480 nt of intron 51, was replaced by the corresponding 1,833-nt sequence of the human *DMD* gene, including the last 800 nt of intron 50, the 233-nt exon 51, and the first 800 nt of intron 51. (***A***)**.** Schematic of ASO dosing schedule in huEx51 mice. ASOs were subcutaneously administered to 8-week-old male huEx51 mice at 100 or 200 mg/kg on days 0 and 2. On days 15, 30, 60 and 120, mice were euthanized and muscle tissues were collected. (***B***)**.** Exon 51 skipping in QU, DI, TR and heart was analyzed by sqRT-PCR. Quantitation of data is shown on the bottom ($n = 3$). Saline, vehicle control; Δ51: exon 51-skipped; Δ51-52: both exons (51 and 52) skipped. * $P < 0.05$, ** $P < 0.01$, *** $P < 0.001$, all compared with Etep at the same harvesting time points.

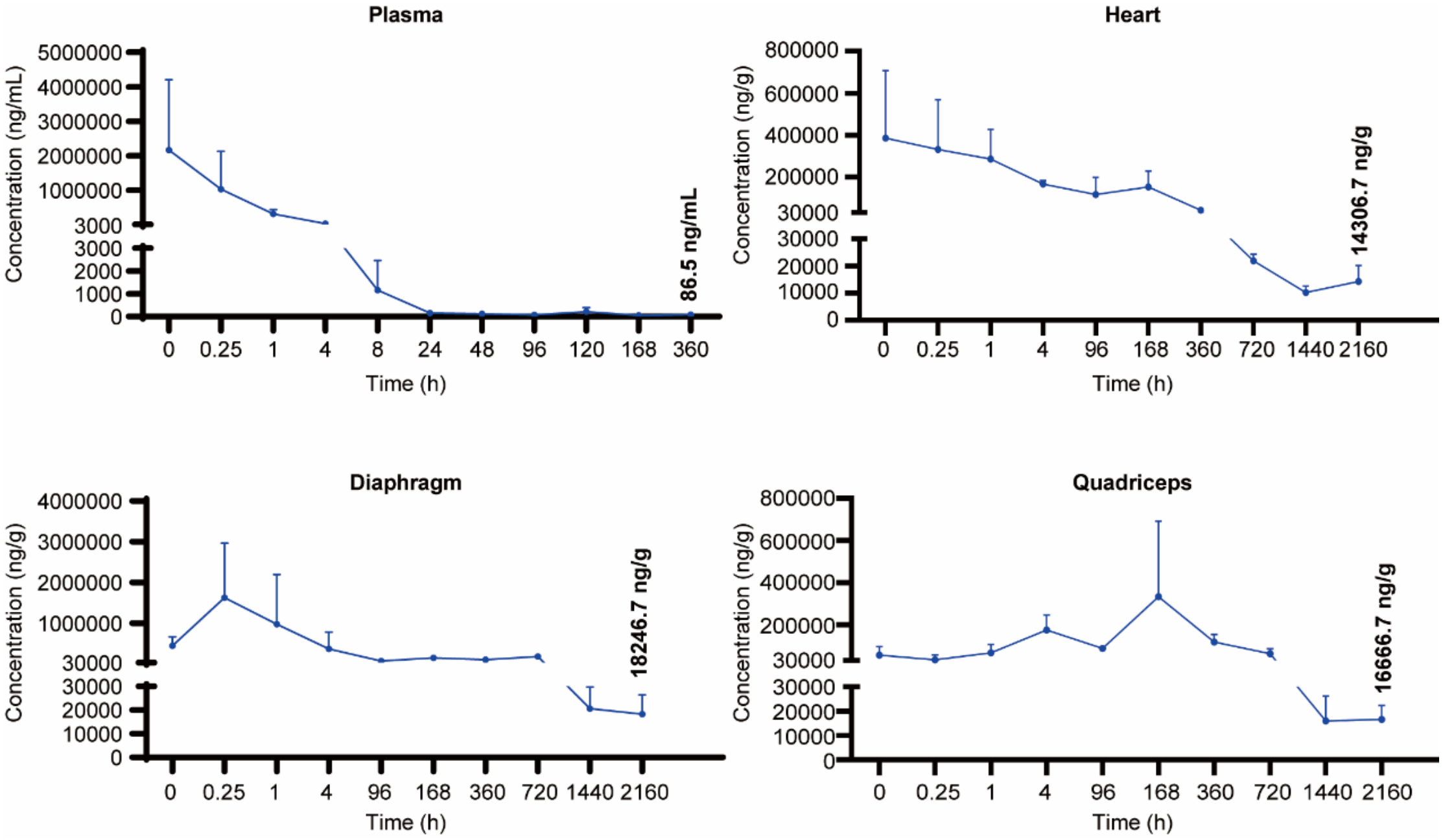


**Supplementary Fig. 7 | Pharmacokinetic (PK) analysis of Etep-L8c in Δ52 mice.** The Δ52 DMD mouse model was purchased from GemPharmatech (strain number: T049591), in which a genomic fragment from the latter part of intron 51 to the front part of intron 52 was deleted. 6-8-week-old male mice received one intravenous injection of Etep-L8c at 100 mg/kg every other day for a total of two injections. After the second injection, mice ($n = 3$) were euthanized at different time points up to 2160 h (90 days); the time point immediately after the second injection was set as 0 h. Four tissues (blood, heart, diaphragm, and quadriceps) were collected and analyzed by liquid chromatography-mass spectrometry using Waters Alliance HPLC system (Waters Corp., Milford, MA, USA) and AB SCIEX Triple Quad™ 6500 tandem mass spectrometer (SCIEX, Framingham, MA, USA). Etep-L8c remained at relatively high levels (>14,300 ng/g) in muscle tissues 2160 h after treatment, whereas its plasma levels sharply dropped from above 2,000,000 ng/mL to 159.8 ng/mL at 24 h and 86.5 ng/mL at 360 h after the treatment.

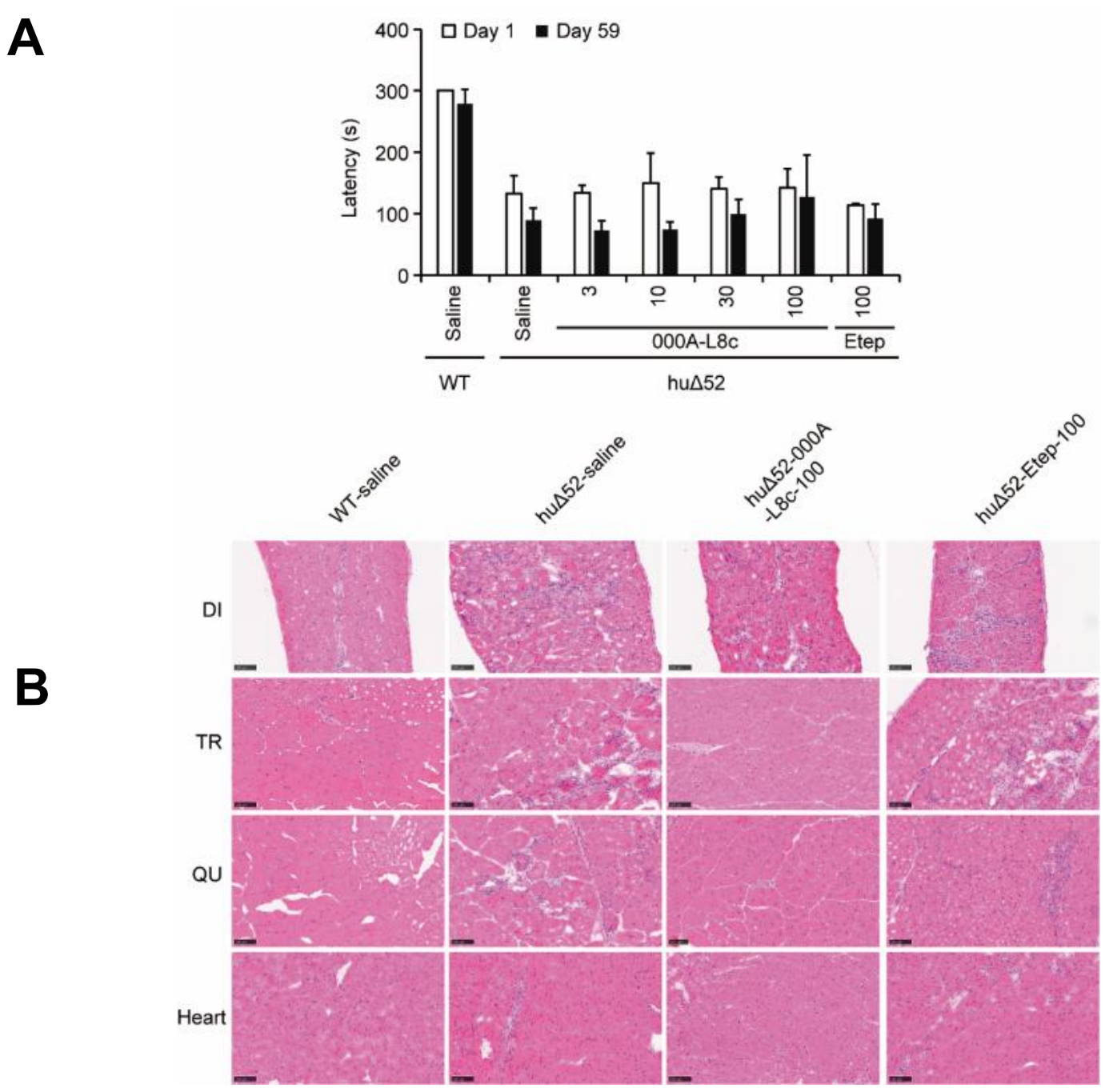


**Supplementary Fig. 8 | Rotarod test and tissue staining of huΔ52 mice after treatment with 000A-L8c.** Mice were injected via the tail vein with 000A-L8c at different doses (3, 10, 30 and 100 mg/kg) or 100 mg/kg Etep, once a week for five consecutive weeks, as shown in **Fig. 4*D***. (***A***)**.** Rotarod test was performed on day 1 (before treatment) and day 59 (after treatment) ($n$ = 5) with a 5-channel rotarod apparatus. Pre-trained mice were placed on the rotating rod with an accelerating speed from 5 to 40 rpm over a span of 300 s. Three trials at 15-min intervals were performed for each mouse and fall latency was recorded. No significant improvement was observed, which is likely due to high intra-group variability, as the coefficient of variation in the 100 mg/kg 000A-L8c-treated group exceeded 30%. (***B***). Cross sections of diaphragm (DI), triceps brachii (TR), quadriceps femoris (QU), and heart tissues from huΔ52 mice treated with 000A-L8c (100 mg/kg), Etep (100 mg/kg), or saline, or from WT mice treated with saline. 000A-L8c but not Etep improved muscle pathology. Muscle tissues were fixed in 4% (v/v) formaldehyde at 4 °C overnight and embedded in paraffin. 5-μm tissue sections were cut for standard H&E staining. Images were captured using a DMIL inverted microscope (Leica, Wetzlar, Germany). The severity of muscle injury was scored based on myofiber necrosis, regeneration, inflammatory cell infiltration and fatty infiltration. Blank areas are due to ice-crystal artifacts during tissue processing. Scale bar = 100 μm.

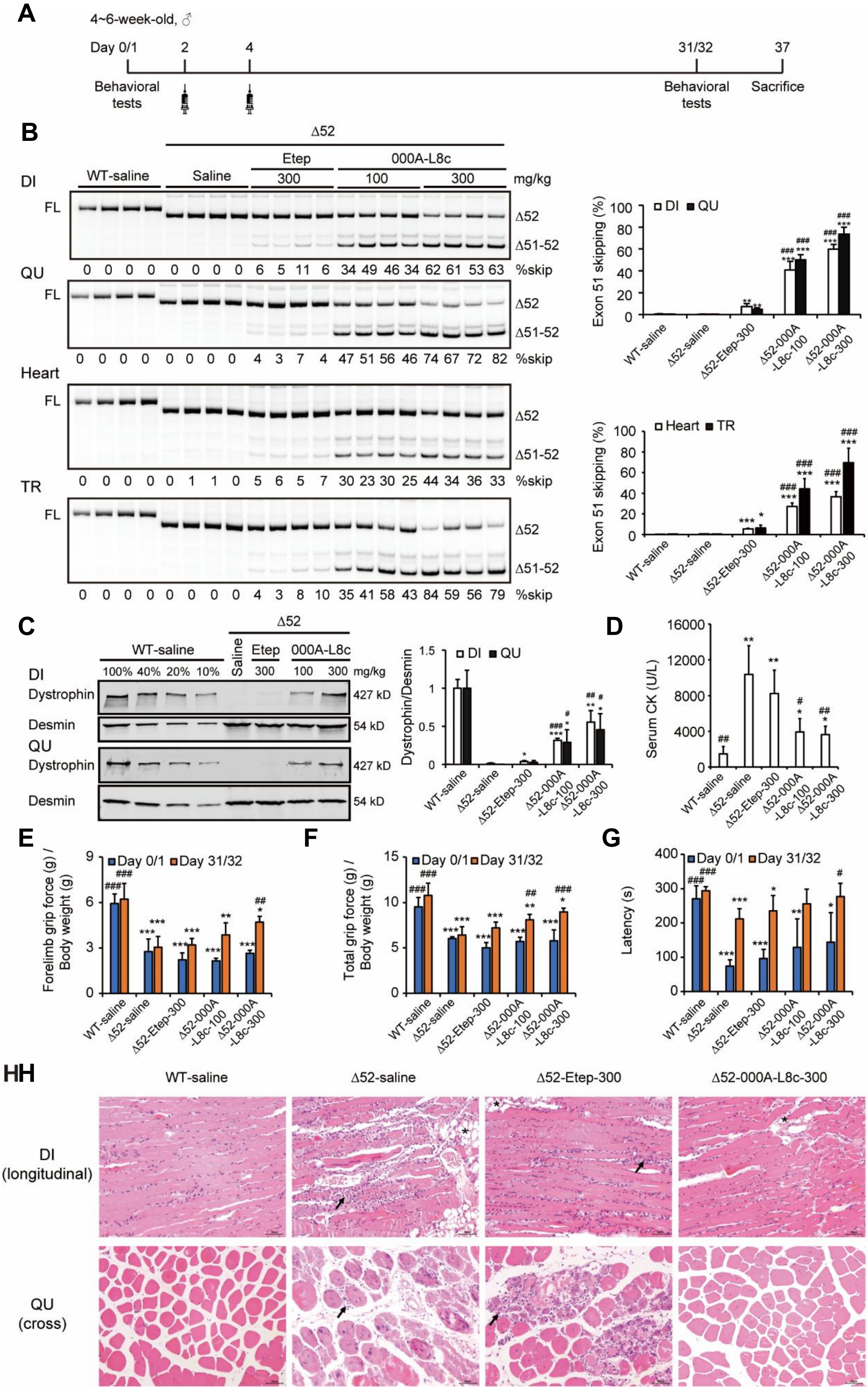
A
4~6-week-old, ♂
Day 0/1
2
4
31/32
37
Behavioral tests
Behavioral tests
Sacrifice
B
Δ52
WT-saline
Saline
Etep
000A-L8c
300
100
300
mg/kg
DI
QU
Heart
TR
FL
Δ52
Δ51-52
%skip
Exon 51 skipping (%)
DI
QU
Heart
TR
WT-saline
Δ52-saline
Δ52-Etep-300
Δ52-000A-L8c-100
Δ52-000A-L8c-300
C
WT-saline
100%
40%
20%
10%
Saline
Etep
000A-L8c
Dystrophin
Desmin
427 kD
54 kD
Dystrophin/Desmin
D
Serum CK (U/L)
E
Day 0/1
Day 31/32
Forelimb grip force (g) / Body weight (g)
F
Total grip force (g) / Body weight (g)
G
Latency (s)
H
WT-saline
Δ52-saline
Δ52-Etep-300
Δ52-000A-L8c-300
DI (longitudinal)
QU (cross)

**Supplementary Fig. 9 | Comparison of therapeutic efficacy between 000A-L8c and Etep in the Δ52 mouse model.** (***A***)**.** Schematic of ASO dosing schedule in Δ52 mice. ASOs were subcutaneously administered to 4-6-week-old male mice at 100 or 300 mg/kg on days 2 and 4. On day 37, mice were euthanized, and muscle tissues were collected. (***B***)**.** 000A-L8c enhanced exon 51 skipping in DI, QU, TR, and heart. Quantitation of exon 51 skipping (% of the transcript lacking both exons) is shown on the right ($n$ = 4). (***C***)**.** 000A-L8c enhanced dystrophin protein levels in DI and QU. Desmin was used as loading control. Relative band intensities were quantitated (right, $n$ = 3). (***D***)**.** 000A-L8c reduced serum CK levels ($n$ = 4). Forelimb grip strength (***E***), four-limb grip strength (***F***), and latency of rotarod test (***G***) were measured on day 0/1 and day 31/32 ($n$ = 5). (***H***)**.** 000A-L8c improved muscle pathology in DI (longitudinal) and QU (cross) ($n$ = 3). Scale bar = 50 μm. Arrows, inflammatory infiltration; asterisks, fatty infiltration. FL: full-length transcript in WT mice, Δ52: transcript without exon 52; Δ51-52: transcript lacking both exons 51 and 52. For panels (***B*** and ***C***), * $P < 0.05$, ** $P < 0.01$, *** $P < 0.001$, all compared with Δ52-saline; $^{\#}$ $P < 0.05$, ## $P < 0.01$, $^{\#\#\#}$ $P < 0.001$, all compared with Etep-treated Δ52 mice. For panels (***D*** and ***E-G***), * $P < 0.05$, ** $P < 0.01$, *** $P < 0.001$, all compared with saline-treated WT mice on the same days; # $P < 0.05$, ## $P < 0.01$, ### $P < 0.001$, all compared with saline-treated Δ52 mice on the same days.

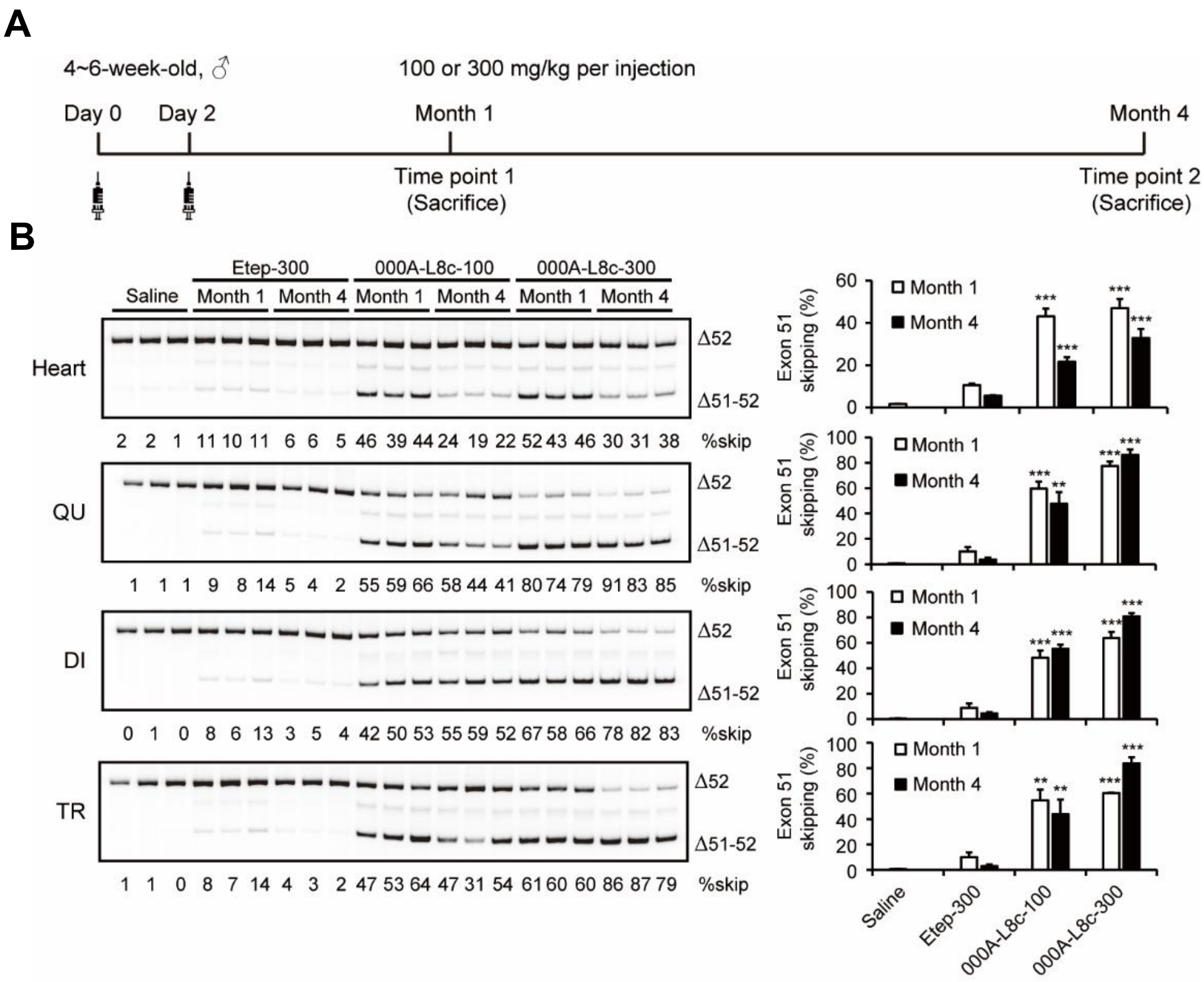


**Supplementary Fig. 10 | The effect of 000A-L8c on Δ52 mice at one and four months post-treatment.** (***A***). Schematic of ASO dosing schedule in Δ52 mice. ASOs were subcutaneously administered to 4-6-week-old male Δ52 mice at 100 or 300 mg/kg on days 0 and 2. Mice were euthanized at one or four months after the second injection, and four muscle tissues (heart, QU, DI and TR) were collected. (***B***)**.** Exon 51 skipping in the four tissues was analyzed by sqRT-PCR. Quantitation of exon 51 splicing is shown on the right ($n = 3$). * $P < 0.05$, ** $P < 0.01$, *** $P < 0.001$, all compared with Etep-300 at the same harvesting time points.

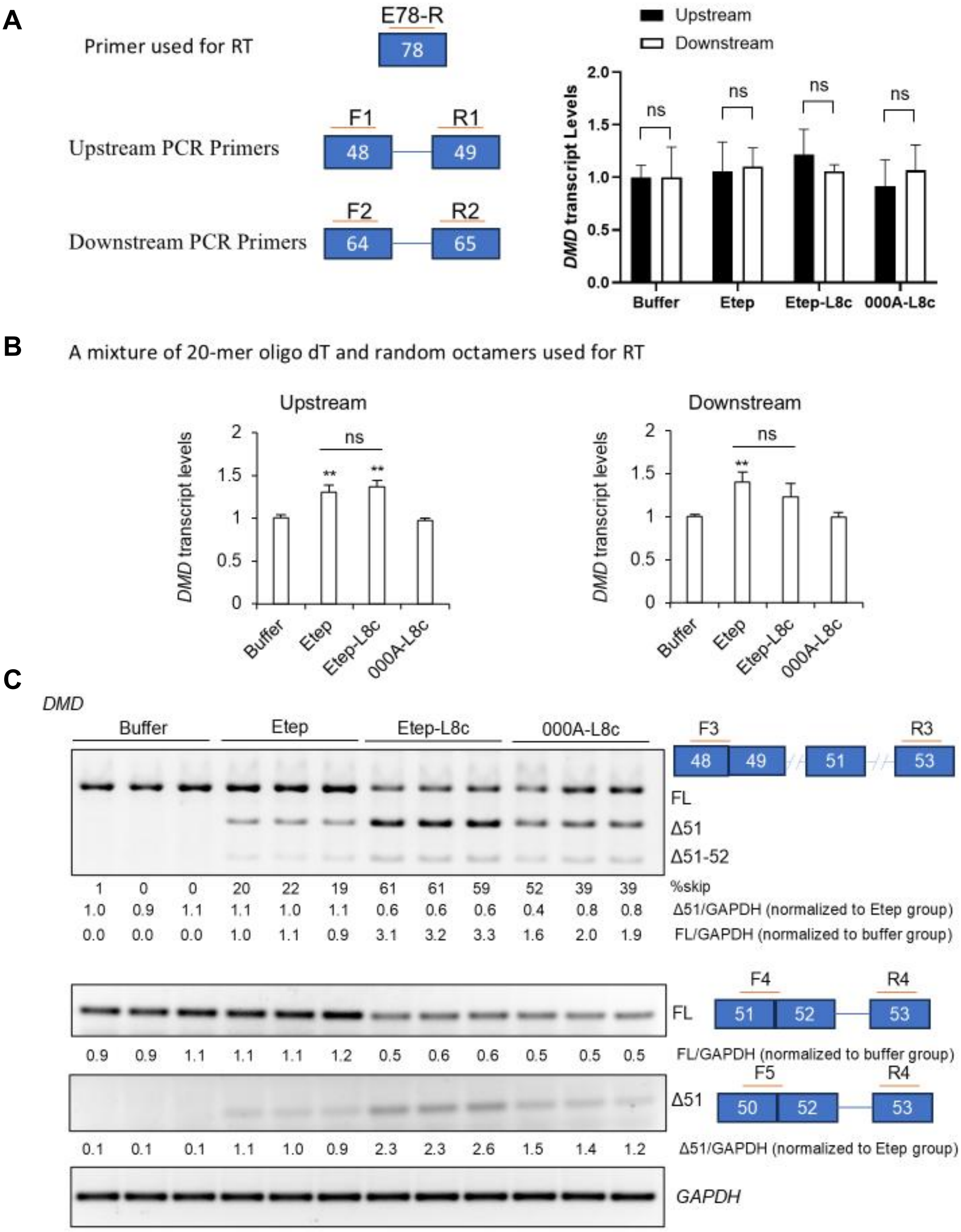
A
Primer used for RT
E78-R
78
Upstream PCR Primers
F1
R1
48
49
Downstream PCR Primers
F2
R2
64
65
Upstream
Downstream
DMD transcript Levels
ns
Buffer
Etep
Etep-L8c
000A-L8c
B
A mixture of 20-mer oligo dT and random octamers used for RT
Upstream
Downstream
DMD transcript levels
C
DMD
FL
Δ51
Δ51-52
%skip
Δ51/GAPDH (normalized to Etep group)
FL/GAPDH (normalized to buffer group)
F3
R3
F4
R4
F5
GAPDH

**Supplementary Fig. 11 | RT-PCR analysis of *DMD* transcripts with primers that target regions outside of the ASO targets.** RD cells were transfected with 25 nM Etep, Etep-L8c, or 000A-L8c; buffer was used as a negative control. 30 h after transfection, cells were harvested and total RNA samples were isolated for splicing analysis (*n* = *3*). The primer set targets exons 48 and 49 for upstream qRT-PCR (upRT-PCR), or exons 64 and 65 for downstream qRT-PCR (dwRT-PCR). ***(A).*** A specific primer targeting exon 78 (E78-R) was used for the reverse transcription (RT) reaction and both upRT-PCR and dwRT-PCR assays were performed. All data were normalized to the buffer group and the mean value of the buffer group was set as 1. No significant differences in *DMD* transcript levels were observed between the upRT-PCR and dwRT-PCR assays in cells treated with either ASO, indicating that neither ASO at 25 nM interfered with the RT reaction. ***(B).*** A mixture of 20-mer oligo dT and random 8-nt primers was used for the RT reaction, and both upRT-PCR and dwRT-PCR assays were performed. All data were normalized to the buffer group. In both qRT-PCR assays, 000A-L8c treatment resulted in similar transcript levels compared to the buffer control, indicating that the 5D ASO treatment does not affect reverse transcription efficiency and mRNA stability. Interestingly, transfection with MOE-Etep (Etep) and Etep-L8c led to a slight increase in total *DMD* transcript levels compared to the buffer control. ***(C).*** The same samples in panels ***A*** and ***B*** were also analyzed by sqRT-PCR. A primer set targeting the junction of exons 48 and 49 and exon 53 was used for all transcripts. A primer set targeting the junction of exons 51 and 52 and exon 53 was used only for the full-length (FL) transcript, and a primer set targeting the junctions of exons 50 and 52 and exon 53 was used for the exon 51-skipped transcript (Δ51). Δ51-52 represents both exons skipped. The data obtained from two single-transcript sqRT-PCR analyses are consistent overall with the data from the all-transcript sqRT-PCR analysis. *GAPDH* was used as an internal control. Primer information is as follows (from 5′ to 3′): E78-R: CATCTGTCATGACTGATACTAAGGACTC; F1: CTGTGGTTATCTCCTATTAGG AATCAGTTGG; R1: CTCTTCCACATCCGGTTGTTTAGCTTGAAC; F2: GCCATG AAACTCCGAAGACTG; R2: AGGC GGTCATAAATAGTGGTCA; F3 and R3: the same as Exon48-Ex49-F and Ex53-22to43-R (see **Table S9**); F4: GATGATCATCAAG CAGAAGGCAAC; R4: GGTGTTCTTGTACTTCATCCCACTG; F5: GACCACTAT TGGAGCCTGCAAC. ** $P < 0.01$, all compared with buffer; ns, not significant.

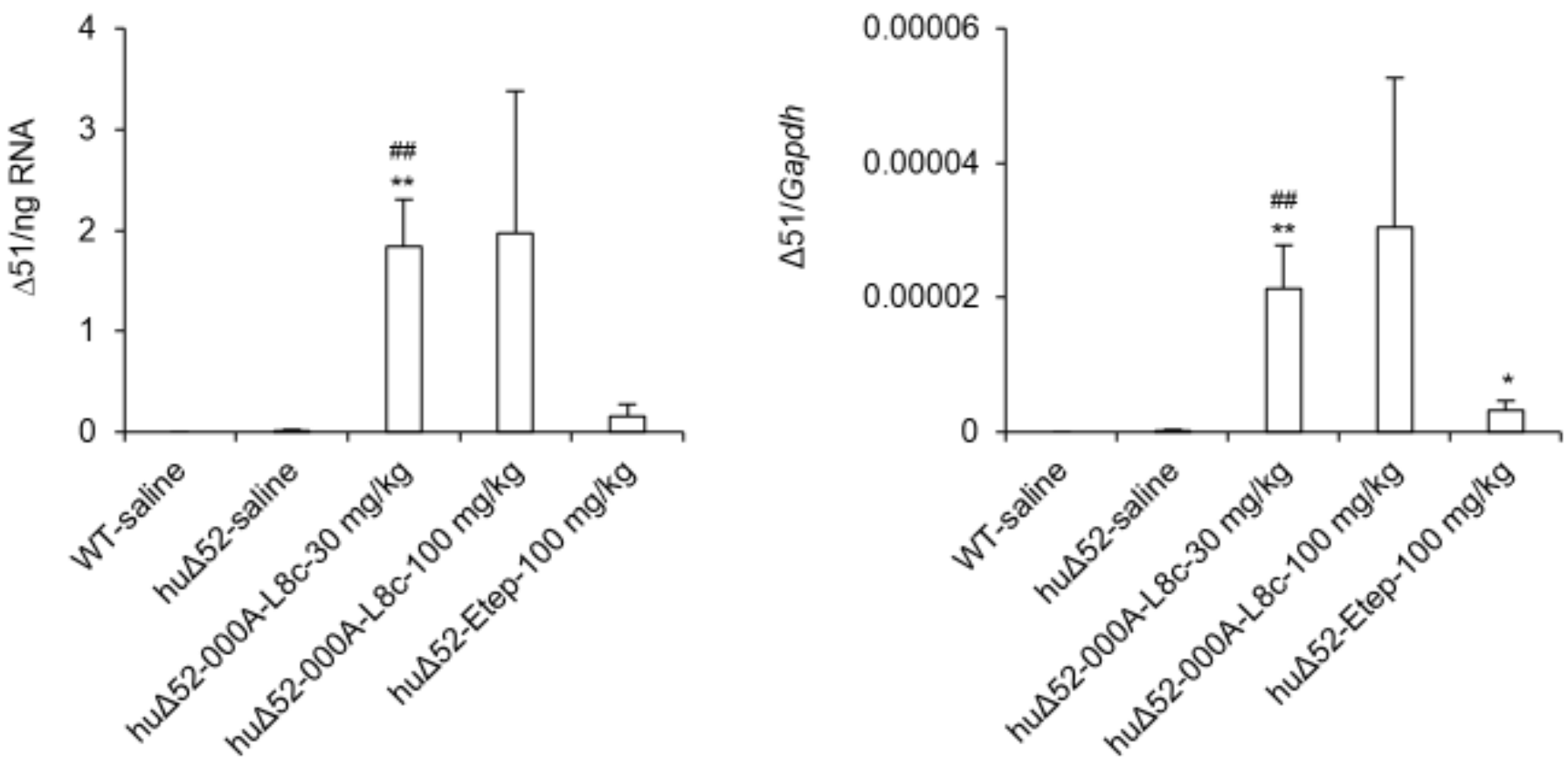


**Supplementary Fig. 12 | Droplet digital PCR (ddPCR) analysis of mouse tissues.** The same quadriceps RNA samples as in **Fig. 4E** were used, and ddPCR was performed on the SG-16-3000 system (RainSure, Suzhou, China). Post-amplification, droplets were read on a Dscanner4-1000 droplet reader (RainSure), and absolute gene copy numbers were calculated using Genecount software with Poisson statistics (LuminUltra Technologies, Fredericton, New Brunswick, Canada). Copies of the *DMD* exon 51-skipped transcript per ng RNA (Δ51/ng) and the ratio of the Δ51 copies to *Gapdh* (Δ51/*Gapdh*) were calculated. *Gapdh* was used as an internal control. The primers and probes used were as follows (5′ to 3′): for Δ51, forward primer: CCACTATTGGAGCC TTTGAAAG, reverse primer: AGACCTGCTCAGCTTCTTCC, and probe: ACACCT TCAGAACCGGAGGCAACAGTT with 5′-HEX and 3′-MGB modifications; for *Gapdh*, forward primer: CATCACTGCCACCCAGAAGACTG, reverse primer: ATGC CAGTGAGCTTCCCGTTCAG, and probe: CCTGCATCCACTGGTGCTGCCAAG GCT with 5′-Cy5.5 and 3′-MGB modifications. * $P < 0.05$, ** $P < 0.01$, all compared with huΔ52-saline; ## $P < 0.01$, all compared with huΔ52-Etep-100 mg/kg.

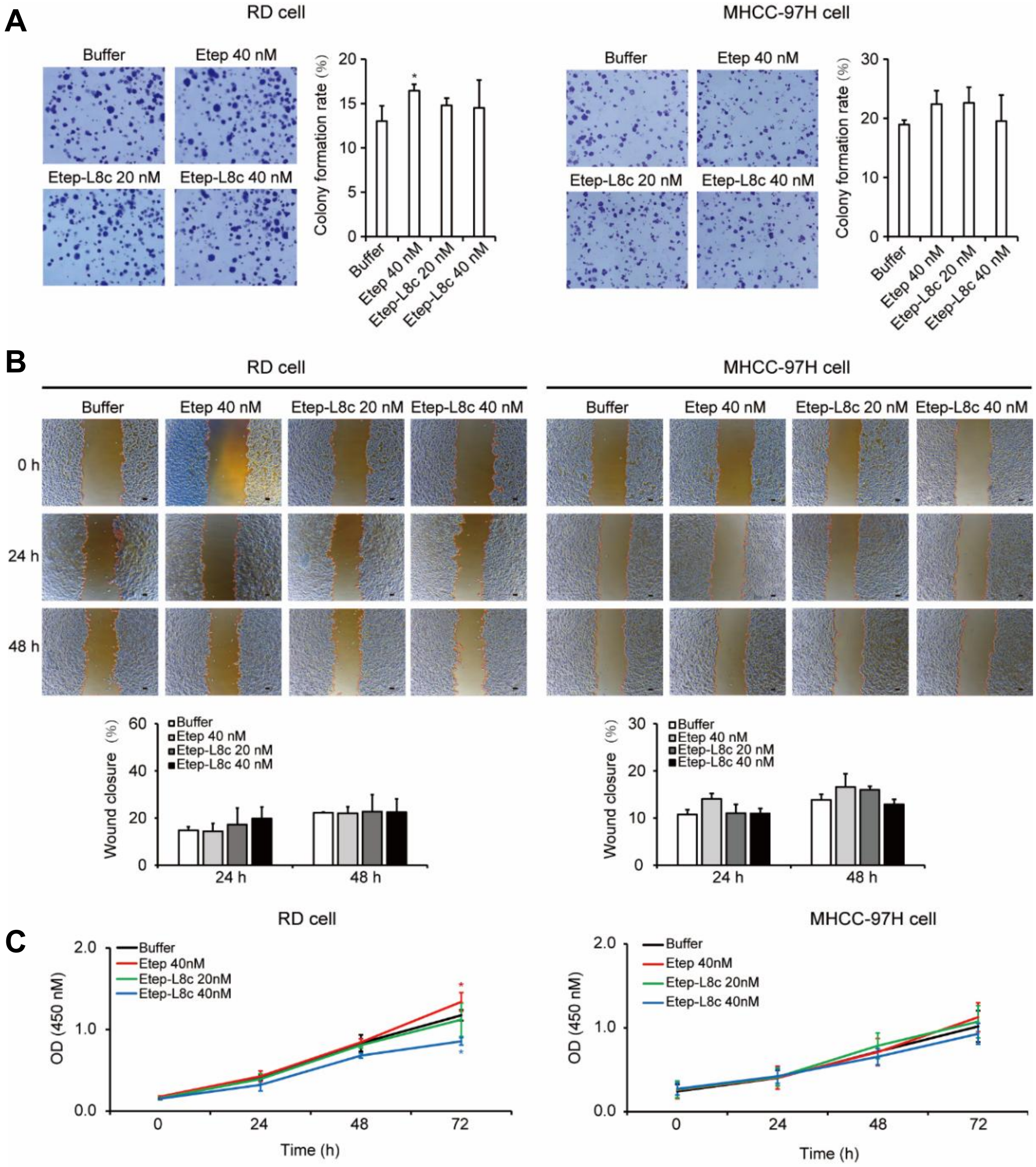


**Supplementary Fig. 13 | The effects of Etep-L8c and Etep on proliferation of RD and MHCC-97H cells.** $10^6$ cells/well were seeded into 6-well plates. Buffer, 40 nM Etep, and 20 or 40 nM Etep-L8c were transfected at 80-90% confluence, respectively. (***A***)**.** For the colony-forming assay, transfected cells were seeded into 6-well plates at a density of 1,000 cells/well. After 10 days, cells were fixed with 4% paraformaldehyde for 20 min, stained with crystal violet staining solution for 30 min, followed by colony counting. (***B***)**.** For the wound-healing assay, a scratch was made in the middle of each well using a pipette tip, 5 h post-transfection. Cells were incubated in medium supplemented with 1% FBS. The wounds were photographed under a microscope at 0, 24, and 48 h. (***C***)**.** For the cell-viability assay, transfected cells were seeded into 96-well plates at a density of 3,000 cells/well, and then incubated for 0, 24, 48, or 72 h. After using a TransDetect® Cell Counting Kit, the OD value at 450 nm was measured on a microplate reader. Buffer: sham transfection. Scale bar = 100 µm. For panels (***A-C***), the results from three independent experiments are quantitated. $*P < 0.05$, all compared with buffer.

**Supplementary Table 1.** The mouse body weights and forces (n =3) recorded in the grip strength test as in **Fig. 4*G***.

| Group | Weight (g) Day 0 | Force 1 (g) Day 0 | Force 2 (g) Day 0 | Force 3 (g) Day 0 | Weight (g) Day 58 | Force 1 (g) Day 58 | Force 2 (g) Day 58 | Force 3 (g) Day 58 |
|---|---|---|---|---|---|---|---|---|
| WT-saline | 21.8 | 211.5 | 217 | 217 | 30.8 | 274 | 268 | 281 |
| | 24.2 | 230.5 | 228 | 210.5 | 34.9 | 252 | 251 | 260 |
| | 24.1 | 195.5 | 199.5 | 199 | 32.1 | 269 | 256 | 254 |
| | 22.8 | 212.5 | 212 | 211.5 | 29.9 | 233.5 | 235 | 230 |
| | 23.9 | 192 | 181 | 181.5 | 30.7 | 264 | 250 | 242 |
| huΔ52-saline | 21 | 157 | 165 | 160 | 30.2 | 93 | 97 | 100 |
| | 21 | 158.5 | 165 | 140 | 37 | 113 | 107 | 124 |
| | 22 | 160 | 164 | 164 | 31.2 | 76 | 69 | 93 |
| | 22 | 158 | 152.5 | 158 | 31 | 77 | 62 | 80 |
| | 21.2 | 167.5 | 152.5 | 158 | 30.4 | 97 | 117 | 107 |
| huΔ52-000A-L8c-3 mg/kg | 21.9 | 189 | 186 | 183 | 35.3 | 94 | 109 | 113 |
| | 20.9 | 134.5 | 131.5 | 134 | 31.7 | 179 | 165 | 180 |
| | 24.2 | 200 | 186 | 162 | 35.9 | 159 | 174 | 168 |
| | 19.7 | 138 | 136.5 | 140.5 | 26.5 | 144 | 151 | 166 |
| | 23.3 | 156 | 149 | 143 | 33.4 | 140 | 138 | 130 |
| huΔ52-000A-L8c-10 mg/kg | 19.8 | 150 | 162 | 157 | 29.8 | 96 | 107 | 85 |
| | 18 | 175.5 | 150.5 | 152 | 26.3 | 61 | 63 | 61 |
| | 23.3 | 158.5 | 144 | 159.5 | 37.4 | 133 | 106 | 96 |
| | 20.6 | 134.5 | 147 | 131.5 | 31 | 91 | 87 | 69 |
| | 19 | 124 | 135.5 | 140 | 29.9 | 126 | 123 | 128 |
| huΔ52-000A-L8c-30 mg/kg | 21.4 | 134 | 138.5 | 143.5 | 30.3 | 202 | 220 | 212 |
| | 19.7 | 150.5 | 158.5 | 133.5 | 28.5 | 173 | 184 | 182 |
| | 20 | 164 | 150 | 151.5 | 28.2 | 150 | 166 | 176 |
| | 22.1 | 154.5 | 134 | 148 | 33.1 | 205 | 220 | 216 |
| | 21.3 | 161.5 | 169 | 146.5 | 34.2 | 192 | 204 | 178 |
| huΔ52-000A-L8c-100 mg/kg | 23.2 | 162.5 | 151 | 165 | 32.2 | 193 | 216 | 189 |
| | 21.7 | 163.5 | 166.5 | 141 | 31 | 207 | 203 | 186 |
| | 18.5 | 128 | 128 | 119.5 | 27.8 | 216 | 202 | 185 |
| | 18.6 | 147.5 | 147.5 | 147.5 | 29.8 | 219 | 213 | 195 |
| | 22 | 180.5 | 189.5 | 171.5 | 30.5 | 236 | 225 | 204 |
| huΔ52-Etep-100 mg/kg | 19.9 | 159.5 | 140.5 | 137 | 28.1 | 154 | 156 | 160 |
| | 22.5 | 167.5 | 161.5 | 163 | 32.6 | 71 | 81 | 73 |
| | 22 | 168.5 | 155 | 155.5 | 32.1 | 184 | 173 | 180 |
| | 22.1 | 182.5 | 185.5 | 185.5 | 31.6 | 164 | 180 | 174 |
| | 23.6 | 151.5 | 154.5 | 158 | 36.8 | 172 | 161 | 151 |

**Supplementary Table 2**. Pathology scores, performed by a third-party organization (Cyagen, Suzhou, Jiangsu, China), of heart, triceps brachii, quadriceps femoris, and diaphragm of huΔ52 mice treated with ASOs at 100 mg/kg, as shown in **Fig. 4*D***.

Score: 0, none; 0.5, slight; 1, mild; 2, moderate; 3, severe; 4, profound.

| Heart | | | | | |
|---|---|---|---|---|---|
| Mouse group | No. | Myofiber necrosis | Myofiber fibrosis | Inflammatory infiltration | Total score |
| WT-saline | 1 | 0 | 0 | 0 | 0 |
| | 2 | 0 | 0 | 0.5 | 0.5 |
| | 3 | 0 | 0 | 0 | 0 |
| | *n* = 3 | Mean ± SD = 0.17 ± 0.29 | | | |
| huΔ52-saline | 1 | 3 | 2 | 2 | 7 |
| | 2 | 3 | 1 | 2 | 6 |
| | 3 | 2 | 1 | 1 | 4 |
| | *n* = 3 | Mean ± SD = 5.67 ± 1.53 | | | |
| huΔ52-000A-L8c | 1 | 1 | 0 | 1 | 2 |
| | 2 | 2 | 0 | 1 | 3 |
| | 3 | 1 | 0 | 1 | 2 |
| | *n* = 3 | Mean ± SD = 2.33 ± 0.58 | | | |
| huΔ52-Etep | 1 | 1 | 0 | 1 | 2 |
| | 2 | 2 | 1 | 1 | 4 |
| | 3 | 1 | 0 | 1 | 2 |
| | *n* = 3 | Mean ± SD = 2.67 ± 1.15 | | | |

**Supplementary Table 2 (continued).**

| Triceps brachii | | | | | |
|---|---|---|---|---|---|
| Mouse group | No. | Myofiber necrosis | Myofiber fibrosis | Inflammatory infiltration | Total score |
| WT-saline | 1 | 0 | 1 | 0 | 1 |
| | 2 | 0 | 0 | 0 | 0 |
| | 3 | 0 | 1 | 0 | 1 |
| | $n$ = 3 | Mean ± SD = 0.67 ± 0.58 | | | |
| huΔ52-saline | 1 | 3 | 1 | 2 | 6 |
| | 2 | 2 | 2 | 2 | 6 |
| | 3 | 2 | 2 | 2 | 6 |
| | $n$ = 3 | Mean ± SD = 6.00 ± 0.00 | | | |
| huΔ52-000A-L8c | 1 | 1 | 2 | 1 | 4 |
| | 2 | 2 | 1 | 0.5 | 3.5 |
| | 3 | 1 | 0 | 0.5 | 1.5 |
| | $n$ = 3 | Mean ± SD = 3.00 ± 1.32 | | | |
| huΔ52-Etep | 1 | 4 | 2 | 2 | 8 |
| | 2 | 3 | 2 | 1 | 6 |
| | 3 | 3 | 2 | 2 | 7 |
| | $n$ = 3 | Mean ± SD = 7.00 ± 1.00 | | | |

**Supplementary Table 2 (continued).**

| Quadriceps femoris | | | | | |
|---|---|---|---|---|---|
| Mouse group | No. | Myofiber necrosis | Myofiber fibrosis | Inflammatory infiltration | Total score |
| WT-saline | 1 | 0 | 0 | 0.5 | 0.5 |
| | 2 | 1 | 0 | 0.5 | 1.5 |
| | 3 | 0 | 1 | 0.5 | 1.5 |
| | *n* = 3 | Mean ± SD = 1.67 ± 0.58 | | | |
| huΔ52-saline | 1 | 3 | 3 | 1 | 7 |
| | 2 | 2 | 1 | 1 | 4 |
| | 3 | 3 | 1 | 1 | 5 |
| | *n* = 3 | Mean ± SD = 5.33 ± 1.53 | | | |
| huΔ52-000A-L8c | 1 | 1 | 1 | 0.5 | 2.5 |
| | 2 | 1 | 2 | 0.5 | 3.5 |
| | 3 | 2 | 2 | 2 | 6 |
| | *n* = 3 | Mean ± SD = 4.00 ± 1.80 | | | |
| huΔ52-Etep | 1 | 2 | 1 | 2 | 5 |
| | 2 | 3 | 1 | 2 | 6 |
| | 3 | 2 | 2 | 1 | 5 |
| | *n* = 3 | Mean ± SD = 5.33 ± 0.58 | | | |

**Supplementary Table 2 (continued).**

| Diaphragm | | | | | |
|---|---|---|---|---|---|
| Mouse group | No. | Myofiber necrosis | Myofiber fibrosis | Inflammatory infiltration | Total score |
| WT-saline | 1 | 0 | 0 | 0 | 0 |
| | 2 | 0 | 1 | 0.5 | 1.5 |
| | 3 | 0 | 0 | 0.5 | 0.5 |
| | *n* = 3 | Mean ± SD = 0.67 ± 0.76 | | | |
| huΔ52-saline | 1 | 3 | 4 | 3 | 10 |
| | 2 | 3 | 3 | 2 | 8 |
| | 3 | 2 | 2 | 2 | 6 |
| | *n* = 3 | Mean ± SD = 8.00 ± 2.00 | | | |
| huΔ52-000A-L8c | 1 | 0 | 2 | 0.5 | 2.5 |
| | 2 | 0 | 1 | 0.5 | 1.5 |
| | 3 | 1 | 1 | 1 | 3 |
| | *n* = 3 | Mean ± SD = 2.33 ± 0.76 | | | |
| huΔ52-Etep | 1 | 0 | 2 | 2 | 4 |
| | 2 | 3 | 0 | 2 | 5 |
| | 3 | 1 | 0 | 1 | 2 |
| | *n* = 3 | Mean ± SD = 3.67 ± 1.53 | | | |

**Supplementary Table 3.** Pathology scores, performed by a third-party organization (OGpharma, Nanjing, Jiangsu, China), of quadriceps femoris and diaphragm of Δ52 mice treated with ASOs at 300 mg/kg, as shown in **Supplementary Fig. 9**.

Score: 0, none; 0.5, slight; 1, mild; 2, moderate; 3, severe; 4, profound.

| Quadriceps femoris | | | | | |
|---|---|---|---|---|---|
| Mouse group | No. | Myofiber necrosis | Myofiber regeneration | Inflammatory infiltration | Total score |
| Δ52-saline | 1 | 1 | 0 | 0.5 | 1.5 |
| | 2 | 1 | 0 | 0.5 | 1.5 |
| | 3 | 1 | 0 | 1 | 2 |
| | *n* = 3 | Mean ± SD = 1.67 ± 0.29 | | | |
| Δ52-Etep | 1 | 0.5 | 0 | 0.5 | 1 |
| | 2 | 2 | 1 | 1 | 4 |
| | 3 | 0.5 | 0 | 0.5 | 1 |
| | *n* = 3 | Mean ± SD = 2.00 ± 1.73 | | | |
| Δ52-000A-L8c | 1 | 0.5 | 0 | 0 | 0.5 |
| | 2 | 0 | 0 | 0 | 0 |
| | 3 | 0 | 0 | 0 | 0 |
| | *n* = 3 | Mean ± SD = 0.17 ± 0.29 | | | |
| WT-saline | 1 | 0 | 0 | 0 | 0 |
| | 2 | 0 | 0 | 0 | 0 |
| | 3 | 0 | 0 | 0 | 0 |
| | *n* = 3 | Mean ± SD = 0.0 ± 0.00 | | | |

**Supplementary Table 3 (continued).**

| Diaphragm | | | | | | |
|---|---|---|---|---|---|---|
| Mouse group | No. | Myofiber necrosis | Fatty infiltration | Inflammatory infiltration | Myofiber regeneration | Total score |
| Δ52-saline | 1 | 0 | 0 | 0.5 | 0 | 0.5 |
| | 2 | 0.5 | 0.5 | 1 | 0 | 2 |
| | 3 | 1 | 1 | 2 | 0 | 4 |
| | *n* = 3 | Mean ± SD = 2.17 ± 1.76 | | | | |
| Δ52-Etep | 1 | 0 | 0 | 2 | 0 | 2 |
| | 2 | 1 | 0 | 2 | 0 | 3 |
| | 3 | 0 | 1 | 0 | 0.5 | 1.5 |
| | *n* = 3 | Mean ± SD = 2.17 ± 0.76 | | | | |
| Δ52-000A-L8c | 1 | 0 | 0 | 0 | 0 | 0 |
| | 2 | 0.5 | 0.5 | 0.5 | 0 | 1.5 |
| | 3 | 0 | 0.5 | 0 | 0 | 0.5 |
| | *n* = 3 | Mean ± SD = 0.7 ± 0.76 | | | | |
| WT-saline | 1 | 0 | 0 | 0 | 0 | 0 |
| | 2 | 0 | 1 | 0 | 0 | 1 |
| | 3 | 0 | 0 | 0 | 0 | 0 |
| | *n* = 3 | Mean ± SD = 0.3 ± 0.58 | | | | |

**Supplementary Table 4.** Blood biochemical data of wild-type C57BL/6N mice (*n* = 7-10) 30 days after the initial ASO treatment. Wild-type mice were injected via the tail vein with 100 mg/kg Etep-L8c or 000A-L8c, with saline as control, once a week for five consecutive weeks. Blood was collected from the abdominal aorta of mice anesthetized with isoflurane, and serum was separated by centrifugation at 3,000 rpm for 15 min at 4 °C. Blood biochemical tests, including aspartate transaminase (AST), alanine transaminase (ALT), creatine kinase (CK), alkaline phosphatase (ALP), urea, creatinine (CREA), total protein (TP), albumin (ALB), globulin (GLB), glucose (GLU), total bilirubin (TBil), total cholesterol (TC), triglyceride (TG) and lactic dehydrogenase (LDH), were performed using a Hitachi 7180 instrument. Biochemical analysis was performed by a third-party organization, SAIFU Laboratories (Beijing, China). Normal ranges of these biochemical parameters were obtained from Charles River Laboratories (www.criver.com). N.A., not available.

| Tests (mean ± SD) | Saline | Etep-L8c | 000A-L8c | Normal ranges (approx. 95% Interval) |
|---|---|---|---|---|
| AST (U/L) | 43.05 ± 11.67 | 34.43 ± 6.64 | 40.91 ± 7.02 | 35.70-111.20 |
| ALT (U/L) | 30.83 ± 8.31 | 32.57 ± 5.47 | 38.66 ± 6.30 | 16.00-79.30 |
| CK (U/L) | 49.3 ± 25.7 | 41.6 ± 11.1 | 40.9 ± 11.5 | N.A. |
| ALP (U/L) | 93.34 ± 12.09 | 85.67 ± 23.05 | 91.44 ± 37.74 | 30.70-70.40 |
| CREA (μmol/L) | 2.94 ± 1.06 | 4.89 ± 1.86 | 7.53 ± 3.73 | 7.07-21.22 |
| UREA (mmol/L) | 7.47 ± 0.79 | 7.60 ± 1.01 | 8.03 ± 0.77 | 5.75-11.60 |
| TP (g/L) | 48.23 ± 1.71 | 47.89 ± 4.18 | 49.53 ± 2.66 | 46.70-62.90 |
| ALB (g/L) | 30.70 ± 1.58 | 30.18 ± 2.17 | 30.03 ± 1.63 | 24.20-34.90 |
| GLB (g/L) | 17.53 ± 1.16 | 17.71 ± 2.36 | 19.50 ± 1.39 | N.A. |
| GLU (mmol/L) | 10.55 ± 1.47 | 10.42 ± 2.57 | 10.26 ± 1.52 | 9.61-20.86 |
| TBIL (μmol/L) | 1.14 ± 0.87 | 1.25 ± 0.60 | 1.02 ± 0.47 | 1.37-3.93 |
| TC (mmol/L) | 2.62 ± 0.43 | 3.27 ± 0.28 | 3.34 ± 0.17 | 2.09-4.22 |
| TG (mmol/L) | 0.64 ± 0.14 | 0.57 ± 0.10 | 0.63 ± 0.17 | 0.62-2.02 |
| LDH (U/L) | 232.6 ± 82.2 | 216.7 ± 63.0 | 237.6 ± 52.5 | N.A. |

**Supplementary Table 5.** Urinalysis results of wild-type mice (*n* = 10) 30 days after the initial ASO treatment. Wild-type mice injected with 100 mg/kg Etep-L8c or 000A-L8c, or saline as in **Supplementary Table 4**. Urine was collected from metabolic cages and assayed with a URIT-500B analyzer. Urinalysis was performed by SAIFU Laboratories (Beijing, China).

| Urinalysis | Saline | Etep-L8c | 000A-L8c |
|---|---|---|---|
| Color | Pale yellow | Pale yellow | Pale yellow |
| Clarity | Slightly cloudy | Slightly cloudy | Slightly cloudy |
| Glucose | Negative | Negative | Negative |
| Bilirubin | Negative | Negative | Negative |
| Ketones | Negative | Negative | Negative |
| Blood | Negative | Negative | Negative |
| Protein | Negative | Negative | Negative |
| Nitrite | Negative | Negative | Negative |
| Leukocyte esterase | Negative | Negative | Negative |
| Urobilinogen | Normal | Normal | Normal |
| pH | 6.5-7.5 | 6.0-7.5 | 7.0 |
| Specific gravity | 1.015-1.025 | 1.015-1.025 | 1.015-1.025 |

**Supplementary Table 6.** Histopathology analysis of liver and kidney from wild-type mice treated with 100 mg/kg Etep-L8c or 000A-L8c, or saline, as shown in **Supplementary Table 4**. Tissue staining was performed by SAIFU Laboratories (Beijing, China).

| Tissue | Appearance | Grade | Number of cases ($n$ = 10) | | |
|---|---|---|---|---|---|
| | | | Saline | Etep-L8c | 000A-L8c |
| Liver | Hepatic Kupffer cells exhibit hypertrophy and basophilic vacuolation. | Normal | 10 | 0 | 0 |
| | | Slight | 0 | 10 | 10 |
| | | Mild | 0 | 0 | 0 |
| | | Moderate | 0 | 0 | 0 |
| | | Severe | 0 | 0 | 0 |
| Kidney | Basophilic granules in renal tubules. | Normal | 10 | 0 | 0 |
| | | Slight | 0 | 10 | 9 |
| | | Mild | 0 | 0 | 1 |
| | | Moderate | 0 | 0 | 0 |
| | | Severe | 0 | 0 | 0 |

**Supplementary Table 7.** Laboratory tests of blood and urine samples collected from cynomolgus monkeys.

| Blood routine tests | | Day | RBC (10^12/L) | HGB (g/L) | HCT (%) | MCV (fL) | MCH (pg) | MCHC (g/L) | RET (10^9/L) | RET (%) | WBC (10^9/L) | NEUT (10^9/L) |
|---|---|---|---|---|---|---|---|---|---|---|---|---|
| Saline | Monkey 1 | -3 | 5.09 | 133 | 42.2 | 82.9 | 26.1 | 315 | 56.0 | 1.10 | 14.72 | 4.59 |
| | | 16 | 5.12 | 132 | 41.7 | 81.4 | 25.8 | 317 | 64.0 | 1.25 | 12.40 | 5.81 |
| | | 64 | 5.30 | 134 | 42.3 | 79.8 | 25.3 | 317 | 65.7 | 1.24 | 13.14 | 4.17 |
| | Monkey 2 | -3 | 5.44 | 126 | 43.1 | 79.2 | 23.2 | 292 | 37.5 | 0.69 | 7.22 | 1.85 |
| | | 16 | 5.32 | 124 | 42.3 | 79.5 | 23.3 | 293 | 45.8 | 0.86 | 5.71 | 1.53 |
| | | 64 | 5.51 | 129 | 43.1 | 78.2 | 23.4 | 299 | 53.4 | 0.97 | 10.51 | 4.49 |
| | Monkey 3 | -3 | 5.17 | 141 | 44.0 | 85.1 | 27.3 | 320 | 93.1 | 1.80 | 12.54 | 2.76 |
| | | 16 | 5.00 | 136 | 42.7 | 85.4 | 27.2 | 319 | 61.0 | 1.22 | 8.41 | 1.52 |
| | | 64 | 4.73 | 128 | 40.0 | 84.6 | 27.1 | 320 | 51.1 | 1.08 | 13.31 | 2.74 |
| | | 86 | 5.12 | 138 | 43.7 | 85.4 | 27.0 | 316 | 54.3 | 1.06 | 12.32 | 1.92 |
| | | 113 | 5.39 | 146 | 44.5 | 82.6 | 27.1 | 328 | 52.8 | 0.98 | 6.98 | 1.48 |
| 000A-L8c, 30 mg/kg | Monkey 1 | -3 | 5.01 | 116 | 38.9 | 77.6 | 23.2 | 298 | 64.1 | 1.28 | 14.75 | 4.35 |
| | | 16 | 4.88 | 115 | 38.3 | 78.5 | 23.6 | 300 | 65.9 | 1.35 | 9.34 | 3.40 |
| | | 64 | 5.47 | 132 | 42.6 | 77.9 | 24.1 | 310 | 45.9 | 0.84 | 12.24 | 3.86 |
| | | 86 | 5.95 | 141 | 46.3 | 77.8 | 23.7 | 305 | 44.6 | 0.75 | 13.04 | 2.38 |
| | Monkey 2 | -3 | 6.24 | 143 | 48.0 | 76.9 | 22.9 | 298 | 36.8 | 0.59 | 13.34 | 2.69 |
| | | 16 | 5.28 | 121 | 41.1 | 77.8 | 22.9 | 294 | 78.7 | 1.49 | 10.15 | 3.07 |
| | | 64 | 5.78 | 134 | 45.4 | 78.5 | 23.2 | 295 | 56.1 | 0.97 | 13.81 | 3.51 |
| | | 86 | 6.13 | 144 | 48.7 | 79.4 | 23.5 | 296 | 71.1 | 1.16 | 16.36 | 4.01 |
| | | 113 | 6.09 | 141 | 46.9 | 77.0 | 23.2 | 301 | 51.8 | 0.85 | 13.87 | 6.95 |
| 000A-L8c, 100 mg/kg | Monkey 1 | -3 | 5.15 | 128 | 42.5 | 82.5 | 24.9 | 301 | 62.8 | 1.22 | 12.01 | 5.22 |
| | | 16 | 4.79 | 119 | 39.3 | 82.0 | 24.8 | 303 | 76.6 | 1.60 | 10.87 | 5.65 |
| | | 64 | 5.75 | 142 | 46.0 | 80.0 | 24.7 | 309 | 39.1 | 0.68 | 9.22 | 4.00 |
| | | 86 | 6.44 | 157 | 50.2 | 78.0 | 24.4 | 313 | 49.6 | 0.77 | 10.82 | 2.99 |
| | Monkey 2 | -3 | 5.62 | 130 | 43.9 | 78.1 | 23.1 | 296 | 36.0 | 0.64 | 11.34 | 4.23 |
| | | 16 | 5.02 | 117 | 39.4 | 78.5 | 23.3 | 297 | 56.7 | 1.13 | 12.19 | 3.32 |
| | | 64 | 5.17 | 127 | 41.7 | 80.7 | 24.6 | 305 | 31.0 | 0.60 | 7.80 | 3.28 |
| | | 86 | 5.75 | 144 | 46.3 | 80.5 | 25.0 | 311 | 40.3 | 0.70 | 7.50 | 1.02 |
| | | 113 | 5.51 | 138 | 44.3 | 80.4 | 25.0 | 312 | 33.6 | 0.61 | 11.81 | 7.15 |

**Supplementary Table 7 (continued).**

| Blood routine tests | | Day | NEUT (%) | LYMPH (10^9/L) | LYMPH (%) | MONO (10^9/L) | MONO (%) | EO (10^9/L) | EO (%) | BASO (10^9/L) | BASO (%) | PLT (10^9/L) |
|---|---|---|---|---|---|---|---|---|---|---|---|---|
| Saline | Monkey 1 | -3 | 31.2 | 9.10 | 61.8 | 0.96 | 6.5 | 0.03 | 0.2 | 0.04 | 0.3 | 544 |
| | | 16 | 46.8 | 6.03 | 48.6 | 0.53 | 4.3 | 0.01 | 0.1 | 0.02 | 0.2 | 528 |
| | | 64 | 31.7 | 8.18 | 62.3 | 0.73 | 5.6 | 0.03 | 0.2 | 0.03 | 0.2 | 560 |
| | Monkey 2 | -3 | 25.6 | 4.65 | 64.4 | 0.54 | 7.5 | 0.17 | 2.4 | 0.01 | 0.1 | 283 |
| | | 16 | 26.7 | 3.77 | 66.0 | 0.38 | 6.7 | 0.02 | 0.4 | 0.01 | 0.2 | 308 |
| | | 64 | 42.7 | 5.09 | 48.4 | 0.83 | 7.9 | 0.09 | 0.9 | 0.01 | 0.1 | 304 |
| | Monkey 3 | -3 | 22.0 | 8.32 | 66.3 | 1.13 | 9.0 | 0.31 | 2.5 | 0.02 | 0.2 | 414 |
| | | 16 | 18.1 | 5.98 | 71.1 | 0.84 | 10.0 | 0.06 | 0.7 | 0.01 | 0.1 | 425 |
| | | 64 | 20.6 | 8.93 | 67.1 | 1.26 | 9.5 | 0.35 | 2.6 | 0.03 | 0.2 | 446 |
| | | 86 | 15.6 | 7.88 | 64.0 | 2.19 | 17.8 | 0.30 | 2.4 | 0.03 | 0.2 | 400 |
| | | 113 | 21.2 | 4.83 | 69.2 | 0.58 | 8.3 | 0.07 | 1.0 | 0.02 | 0.3 | 466 |
| 000A-L8c, 30 mg/kg | Monkey 1 | -3 | 29.5 | 9.72 | 65.9 | 0.59 | 4.0 | 0.06 | 0.4 | 0.03 | 0.2 | 413 |
| | | 16 | 36.4 | 5.39 | 57.7 | 0.45 | 4.8 | 0.09 | 1.0 | 0.01 | 0.1 | 397 |
| | | 64 | 31.5 | 7.79 | 63.6 | 0.45 | 3.7 | 0.12 | 1.0 | 0.02 | 0.2 | 367 |
| | | 86 | 18.3 | 9.56 | 73.3 | 0.71 | 5.4 | 0.36 | 2.8 | 0.03 | 0.2 | 385 |
| | Monkey 2 | -3 | 20.3 | 9.73 | 72.9 | 0.79 | 5.9 | 0.11 | 0.8 | 0.02 | 0.1 | 387 |
| | | 16 | 30.2 | 6.25 | 61.6 | 0.62 | 6.1 | 0.19 | 1.9 | 0.02 | 0.2 | 452 |
| | | 64 | 25.4 | 9.43 | 68.3 | 0.74 | 5.4 | 0.11 | 0.8 | 0.02 | 0.1 | 410 |
| | | 86 | 24.5 | 10.93 | 66.8 | 1.09 | 6.7 | 0.30 | 1.8 | 0.03 | 0.2 | 396 |
| | | 113 | 50.0 | 6.09 | 43.9 | 0.66 | 4.8 | 0.16 | 1.2 | 0.01 | 0.1 | 379 |
| 000A-L8c, 100 mg/kg | Monkey 1 | -3 | 43.4 | 5.92 | 49.3 | 0.80 | 6.7 | 0.06 | 0.5 | 0.01 | 0.1 | 433 |
| | | 16 | 51.9 | 4.39 | 40.4 | 0.76 | 7.0 | 0.06 | 0.6 | 0.01 | 0.1 | 450 |
| | | 64 | 43.3 | 4.35 | 47.2 | 0.79 | 8.6 | 0.07 | 0.8 | 0.01 | 0.1 | 483 |
| | | 86 | 27.7 | 6.85 | 63.3 | 0.77 | 7.1 | 0.20 | 1.8 | 0.01 | 0.1 | 429 |
| | Monkey 2 | -3 | 37.3 | 6.13 | 54.1 | 0.65 | 5.7 | 0.23 | 2.0 | 0.10 | 0.9 | 353 |
| | | 16 | 27.3 | 7.84 | 64.3 | 0.81 | 6.6 | 0.19 | 1.6 | 0.03 | 0.2 | 436 |
| | | 64 | 42.0 | 3.86 | 49.5 | 0.61 | 7.8 | 0.03 | 0.4 | 0.02 | 0.3 | 350 |
| | | 86 | 13.6 | 5.86 | 78.1 | 0.56 | 7.5 | 0.04 | 0.5 | 0.02 | 0.3 | 388 |
| | | 113 | 60.5 | 3.44 | 29.1 | 1.13 | 9.6 | 0.08 | 0.7 | 0.01 | 0.1 | 338 |

**Supplementary Table 7 (continued).**

| Coagulation function tests | | Day | PT (s) | APTT (s) | Fbg C. (g/L) |
|---|---|---|---|---|---|
| Saline | Monkey 1 | -3 | 9.00 | 25.30 | 1.88 |
| | | 16 | 9.80 | 24.50 | 1.93 |
| | | 64 | 9.40 | 24.40 | 1.86 |
| | Monkey 2 | -3 | 8.70 | 23.20 | 1.69 |
| | | 16 | 9.20 | 20.30 | 1.73 |
| | | 64 | 9.50 | 22.90 | 1.54 |
| | Monkey 3 | -3 | 9.20 | 25.40 | 1.71 |
| | | 16 | 9.80 | 23.30 | 1.69 |
| | | 64 | 9.90 | 25.40 | 1.36 |
| | | 86 | 9.60 | 25.50 | 1.63 |
| | | 113 | 9.30 | 23.40 | 1.60 |
| 000A-L8c, 30 mg/kg | Monkey 1 | -3 | 8.70 | 29.20 | 1.93 |
| | | 16 | 9.10 | 27.20 | 2.01 |
| | | 64 | 8.50 | 29.20 | 1.88 |
| | | 86 | 8.80 | 29.40 | 1.93 |
| | Monkey 2 | -3 | 8.90 | 27.00 | 2.10 |
| | | 16 | 9.50 | 27.50 | 2.07 |
| | | 64 | 8.90 | 27.50 | 1.88 |
| | | 86 | 9.10 | 28.90 | 1.89 |
| | | 113 | 8.90 | 25.20 | 1.91 |
| 000A-L8c, 100 mg/kg | Monkey 1 | -3 | 8.60 | 23.50 | 1.81 |
| | | 16 | 9.50 | 25.40 | 2.16 |
| | | 64 | 8.50 | 22.90 | 2.25 |
| | | 86 | 8.30 | 24.00 | 2.29 |
| | Monkey 2 | -3 | 9.40 | 23.70 | 1.63 |
| | | 16 | 10.70 | 25.40 | 1.98 |
| | | 64 | 9.70 | 25.20 | 2.07 |
| | | 86 | 9.20 | 24.10 | 1.98 |
| | | 113 | 9.80 | 23.80 | 4.09 |

**Supplementary Table 7 (continued).**

| Blood biochemical tests | | Day | AST (U/L) | ALT (U/L) | GGT (U/L) | CK (U/L) | ALP (U/L) | UREA (mmol/L) | CREA (μmol/L) | TP (g/L) | ALB (g/L) | GLB (g/L) |
|---|---|---|---|---|---|---|---|---|---|---|---|---|
| Saline | Monkey 1 | -3 | 54.5 | 48.4 | 73.1 | 611 | 473.6 | 7.61 | 54.8 | 66.6 | 40.5 | 26.1 |
| | | 16 | 81.0 | 57.7 | 76.3 | 1135 | 549.5 | 5.78 | 55.2 | 66.9 | 41.4 | 25.5 |
| | | 64 | 45.2 | 47.8 | 89.8 | 352 | 710.1 | 8.28 | 66.7 | 67.4 | 39.4 | 28.0 |
| | Monkey 2 | -3 | 36.6 | 40.0 | 116.8 | 164 | 586.0 | 5.94 | 40.9 | 63.8 | 39.9 | 23.9 |
| | | 16 | 46.4 | 42.4 | 109.5 | 206 | 654.7 | 9.40 | 47.8 | 67.0 | 41.8 | 25.2 |
| | | 64 | 39.9 | 41.7 | 126.6 | 255 | 726.1 | 8.37 | 50.5 | 64.2 | 39.4 | 24.8 |
| | Monkey 3 | -3 | 51.1 | 37.8 | 87.7 | 278 | 712.5 | 7.34 | 63.7 | 72.3 | 43.5 | 28.8 |
| | | 16 | 80.2 | 50.4 | 98.2 | 620 | 963.7 | 5.74 | 56.5 | 72.5 | 44.3 | 28.2 |
| | | 64 | 48.0 | 35.3 | 118.6 | 253 | 1116.5 | 6.35 | 73.2 | 68.2 | 40.6 | 27.6 |
| | | 86 | 48.1 | 36.1 | 124.1 | 280 | 1113.2 | 6.26 | 60.3 | 69.1 | 40.9 | 28.2 |
| | | 113 | 42.9 | 34.1 | 121.6 | 363 | 1139.8 | 7.41 | 70.8 | 72.1 | 43.7 | 28.4 |
| 000A-L8c, 30 mg/kg | Monkey 1 | -3 | 46.7 | 32.7 | 93.6 | 272 | 790.2 | 5.75 | 52.0 | 67.8 | 42.6 | 25.2 |
| | | 16 | 67.5 | 39.4 | 84.9 | 447 | 767.4 | 3.89 | 50.4 | 69.2 | 44.0 | 25.2 |
| | | 64 | 44.0 | 26.6 | 87.6 | 200 | 763.1 | 5.68 | 66.3 | 72.3 | 43.0 | 29.3 |
| | | 86 | 42.9 | 32.2 | 87.4 | 264 | 708.0 | 5.71 | 58.3 | 71.2 | 41.3 | 29.9 |
| | Monkey 2 | -3 | 47.4 | 27.5 | 131.7 | 209 | 687.4 | 7.79 | 50.8 | 81.6 | 49.7 | 31.9 |
| | | 16 | 70.7 | 43.0 | 96.6 | 240 | 694.7 | 7.13 | 47.7 | 72.9 | 45.9 | 27.0 |
| | | 64 | 35.6 | 25.9 | 130.4 | 248 | 846.9 | 6.48 | 61.8 | 72.8 | 44.8 | 28.0 |
| | | 86 | 46.5 | 33.1 | 140.0 | 406 | 832.8 | 6.92 | 56.8 | 78.0 | 45.9 | 32.1 |
| | | 113 | 47.4 | 26.6 | 132.6 | 490 | 735.6 | 6.54 | 68.4 | 67.7 | 42.3 | 25.4 |
| 000A-L8c, 100 mg/kg | Monkey 1 | -3 | 57.9 | 35.5 | 73.9 | 333 | 519.0 | 6.79 | 42.2 | 64.3 | 40.3 | 24.0 |
| | | 16 | 80.9 | 64.6 | 60.4 | 217 | 656.3 | 6.40 | 51.9 | 65.0 | 40.7 | 24.3 |
| | | 64 | 60.5 | 63.3 | 63.0 | 389 | 664.1 | 6.64 | 70.0 | 69.0 | 40.2 | 28.8 |
| | | 86 | 53.9 | 113.7 | 51.7 | 187 | 548.1 | 6.98 | 58.3 | 73.1 | 40.9 | 32.2 |
| | Monkey 2 | -3 | 36.1 | 35.8 | 71.7 | 166 | 387.2 | 5.98 | 57.0 | 74.3 | 36.7 | 37.6 |
| | | 16 | 96.4 | 43.3 | 58.9 | 658 | 386.9 | 5.84 | 63.6 | 69.2 | 35.2 | 34.0 |
| | | 64 | 40.1 | 41.5 | 80.0 | 130 | 550.0 | 6.72 | 68.5 | 74.0 | 35.8 | 38.2 |
| | | 86 | 47.6 | 62.5 | 71.4 | 236 | 559.7 | 8.02 | 73.7 | 74.7 | 37.3 | 37.4 |
| | | 113 | 28.4 | 26.6 | 63.2 | 179 | 401.7 | 7.83 | 89.0 | 71.1 | 35.4 | 35.7 |

## Supplementary Table 7 (continued).

| Blood biochemical tests | | Day | ALB/GLB | GLU (mmol/L) | TBIL (μmol/L) | TC (mmol/L) | TG (mmol/L) | Na (mmol/L) | K (mmol/L) | Cl (mmol/L) | LDH (U/L) |
|---|---|---|---|---|---|---|---|---|---|---|---|
| Saline | Monkey 1 | -3 | 1.55 | 3.1 | 2.2 | 2.72 | 0.39 | 149 | 5.31 | 106 | 503 |
| | | 16 | 1.62 | 2.6 | 2.9 | 3.01 | 0.42 | 150 | 4.70 | 112 | 656 |
| | | 64 | 1.41 | 2.3 | 2.4 | 2.58 | 0.32 | 153 | 4.54 | 111 | 557 |
| | Monkey 2 | -3 | 1.67 | 3.3 | 1.8 | 3.58 | 0.42 | 148 | 4.91 | 108 | 353 |
| | | 16 | 1.66 | 2.1 | 4.4 | 4.17 | 0.56 | 151 | 4.89 | 116 | 497 |
| | | 64 | 1.59 | 3.1 | 2.6 | 3.08 | 0.55 | 151 | 4.54 | 113 | 502 |
| | Monkey 3 | -3 | 1.51 | 4.0 | 1.5 | 3.11 | 0.36 | 148 | 5.18 | 104 | 464 |
| | | 16 | 1.57 | 4.1 | 3.0 | 3.51 | 0.38 | 151 | 4.63 | 110 | 616 |
| | | 64 | 1.47 | 3.8 | 1.3 | 2.94 | 0.26 | 153 | 5.03 | 112 | 679 |
| | | 86 | 1.45 | 4.0 | 1.9 | 2.95 | 0.30 | 148 | 5.07 | 105 | 539 |
| | | 113 | 1.54 | 3.4 | 3.4 | 3.45 | 0.57 | 147 | 4.60 | 108 | 440 |
| 000A-L8c, 30 mg/kg | Monkey 1 | -3 | 1.69 | 3.6 | 3.0 | 3.30 | 0.40 | 146 | 5.41 | 104 | 834 |
| | | 16 | 1.75 | 3.1 | 3.5 | 3.38 | 0.40 | 151 | 5.70 | 112 | 1109 |
| | | 64 | 1.47 | 3.2 | 2.5 | 3.71 | 0.22 | 152 | 4.96 | 107 | 1179 |
| | | 86 | 1.38 | 3.3 | 3.0 | 3.95 | 0.33 | 148 | 5.61 | 108 | 959 |
| | Monkey 2 | -3 | 1.56 | 3.3 | 3.6 | 3.61 | 0.57 | 153 | 5.74 | 104 | 291 |
| | | 16 | 1.70 | 3.3 | 4.4 | 3.15 | 0.43 | 152 | 4.47 | 112 | 334 |
| | | 64 | 1.60 | 3.9 | 3.5 | 3.01 | 0.36 | 158 | 3.60 | 108 | 284 |
| | | 86 | 1.43 | 3.5 | 5.1 | 3.06 | 0.49 | 152 | 4.87 | 105 | 347 |
| | | 113 | 1.67 | 3.5 | 5.5 | 3.06 | 0.50 | 146 | 4.08 | 105 | 413 |
| 000A-L8c, 100 mg/kg | Monkey 1 | -3 | 1.68 | 2.5 | 3.1 | 3.45 | 0.65 | 150 | 4.62 | 107 | 770 |
| | | 16 | 1.67 | 2.5 | 2.7 | 3.40 | 0.57 | 152 | 4.01 | 111 | 524 |
| | | 64 | 1.40 | 3.2 | 1.6 | 4.47 | 0.47 | 155 | 4.31 | 111 | 679 |
| | | 86 | 1.27 | 3.4 | 3.1 | 4.44 | 0.62 | 148 | 4.56 | 106 | 584 |
| | Monkey 2 | -3 | 0.98 | 3.7 | 1.7 | 2.67 | 0.22 | 150 | 6.26 | 108 | 563 |
| | | 16 | 1.04 | 4.6 | 1.9 | 2.22 | 0.29 | 150 | 4.36 | 114 | 455 |
| | | 64 | 0.94 | 3.6 | 1.1 | 2.95 | 0.19 | 155 | 5.16 | 114 | 450 |
| | | 86 | 1.00 | 4.3 | 2.3 | 2.82 | 0.41 | 151 | 6.34 | 110 | 548 |
| | | 113 | 0.99 | 3.8 | 2.1 | 3.14 | 0.34 | 150 | 5.21 | 113 | 368 |

**Supplementary Table 7 (continued).**

| Immunotoxicology tests | | Day | CD45+CD3+ (%) | CD45+CD3+ CD4+CD8- (%) | CD45+CD3+ CD4-CD8+ (%) | CD4+/CD8+ | C3 (mg/dL) | C4 (mg/dL) | CRP (mg/L) |
|---|---|---|---|---|---|---|---|---|---|
| Saline | Monkey 1 | -3 | 46.62 | 26.10 | 17.89 | 1.46 | 81.5 | 14.1 | 1.4 |
| | | 16 | 48.06 | 29.66 | 15.67 | 1.89 | 80.4 | 13.2 | 0.0 |
| | | 64 | 54.23 | 31.97 | 18.77 | 1.70 | 87.7 | 18.6 | 2.3 |
| | Monkey 2 | -3 | 72.39 | 39.07 | 28.07 | 1.39 | 95.4 | 5.1 | 1.8 |
| | | 16 | 75.67 | 41.95 | 28.34 | 1.48 | 96.3 | 6.0 | 2.3 |
| | | 64 | 77.92 | 45.62 | 27.83 | 1.64 | 92.9 | 7.1 | 3.4 |
| | Monkey 3 | -3 | 57.19 | 37.40 | 15.47 | 2.42 | 87.5 | 17.5 | 1.8 |
| | | 16 | 61.83 | 41.30 | 16.11 | 2.56 | 84.5 | 16.0 | 7.9 |
| | | 64 | 65.95 | 44.82 | 16.52 | 2.71 | 84.2 | 18.4 | 1.7 |
| | | 86 | - | - | - | - | 86.5 | 17.5 | 2.0 |
| | | 113 | - | - | - | - | 83.2 | 16.6 | 0.9 |
| 000A-L8c, 30 mg/kg | Monkey 1 | -3 | 54.44 | 24.65 | 24.93 | 0.99 | 92.8 | 14.6 | 3.4 |
| | | 16 | 60.85 | 31.31 | 25.38 | 1.23 | 65.3 | 16.8 | 18.1 |
| | | 64 | 64.71 | 34.03 | 26.22 | 1.30 | 97.4 | 21.0 | 2.7 |
| | | 86 | - | - | - | - | 110.6 | 24.5 | 5.3 |
| | Monkey 2 | -3 | 66.37 | 31.18 | 29.25 | 1.07 | 111.8 | 15.4 | 3.5 |
| | | 16 | 75.76 | 39.89 | 29.22 | 1.37 | 65.6 | 12.9 | 13.7 |
| | | 64 | 74.96 | 35.52 | 33.13 | 1.07 | 93.7 | 16.3 | 2.8 |
| | | 86 | - | - | - | - | 108.0 | 19.8 | 4.9 |
| | | 113 | - | - | - | - | 94.0 | 16.9 | 2.9 |
| 000A-L8c, 100 mg/kg | Monkey 1 | -3 | 69.83 | 24.70 | 37.51 | 0.66 | 94.7 | 16.4 | 8.9 |
| | | 16 | 76.31 | 31.17 | 37.20 | 0.84 | 58.0 | 20.5 | 27.3 |
| | | 64 | 76.74 | 26.64 | 42.37 | 0.63 | 101.9 | 24.4 | 11.3 |
| | | 86 | - | - | - | - | 107.2 | 24.7 | 9.8 |
| | Monkey 2 | -3 | 64.84 | 23.79 | 35.02 | 0.68 | 88.5 | 17.2 | 3.2 |
| | | 16 | 64.87 | 26.03 | 30.44 | 0.86 | 36.7 | 7.5 | 14.5 |
| | | 64 | 58.41 | 26.12 | 24.05 | 1.09 | 81.6 | 20.3 | 9.5 |
| | | 86 | - | - | - | - | 91.2 | 20.6 | 5.4 |
| | | 113 | - | - | - | - | 94.2 | 26.7 | 51.6 |

## Supplementary Table 7 (continued).

| Urinalysis | | Day | UCOL | UCLA | UGLU | UBIL | UKET | UBLD | UpH | UPRO | URO | UNIT | ULEU | US.G. |
|---|---|---|---|---|---|---|---|---|---|---|---|---|---|---|
| Saline | Monkey 1 | 64 | L yellow | – | – | – | – | – | 8.5 | – | Normal | – | – | 1.003 |
| | Monkey 2 | 64 | Straw | 1+ | – | – | – | 1+ | 9.0 | – | Normal | – | – | 1.002 |
| | Monkey 3 | 64 | L yellow | 1+ | – | – | – | – | 8.5 | – | Normal | + | – | 1.006 |
| | | 86 | L yellow | 1+ | – | – | – | + – | 8.5 | – | Normal | – | – | 1.001 |
| | | 113 | Straw | 2+ | – | – | – | + – | 8.5 | – | Normal | – | – | 1.005 |
| 000A-L8c, 30 mg/kg | Monkey 1 | 64 | L yellow | 2+ | – | – | – | + – | 9.0 | – | Normal | – | – | 1.007 |
| | | 86 | Straw | 1+ | – | – | – | + – | 8.5 | – | Normal | + | – | 1.003 |
| | Monkey 2 | 64 | Straw | – | – | – | – | + – | 9.0 | – | Normal | + | – | 1.003 |
| | | 86 | Straw | – | – | – | – | + – | 8.5 | – | Normal | + | – | 1.004 |
| | | 113 | Straw | – | – | – | – | – | 8.0 | – | Normal | – | – | 1.005 |
| 000A-L8c, 100 mg/kg | Monkey 1 | 64 | Yellow | 2+ | – | – | – | – | 9.0 | – | Normal | – | – | 1.027 |
| | | 86 | Straw | – | – | – | – | + – | 8.5 | – | Normal | + | – | 1.004 |
| | Monkey 2 | 64 | Straw | – | – | – | – | + – | 9.0 | 1+ | Normal | + | – | 1.018 |
| | | 86 | Straw | – | – | – | – | – | 7.5 | – | Normal | + | – | 1.005 |
| | | 113 | Straw | – | – | – | – | – | 8.5 | 1+ | Normal | + | – | 1.028 |

**Supplementary Table 8.** List of ASO sequences used in this study. The decoy portion is underlined.

| ASO name | ASO sequence (5′-3′) | Target |
|---|---|---|
| 1938 | TGTGAGCACCTTCCTTCTTT | |
| 1938-L#1 | AGGTATGTGAGCACCTTCCTTCTTT | |
| 1938-L#2 | GTAAGTGTGAGCACCTTCCTTCTTT | |
| 1938-L#3 | AGTATTGTGAGCACCTTCCTTCTTT | |
| 1938-R#1 | TGTGAGCACCTTCCTTCTTTAGGTA | |
| 1938-R#2 | TGTGAGCACCTTCCTTCTTTGTAAG | |
| 1938-R#3 | TGTGAGCACCTTCCTTCTTTAGTAT | |
| 1938-L11 | CAGGTAAGTATTGTGAGCACCTTCCTTCTTT | *SMN1/2* |
| 1938-R11 | TGTGAGCACCTTCCTTCTTTCAGGTAAGTAT | |
| 2203 | GTAAGGAAAATAAAGGAAGT | |
| 2203-L11 | CAGGTAAGTATGTAAGGAAAATAAAGGAAGT | |
| 2203-R11 | GTAAGGAAAATAAAGGAAGTCAGGTAAGTAT | |
| 0120 | CATAATGCTGGCAGACTTAC | |
| 0120-L11 | CAGGTAAGTATCATAATGCTGGCAGACTTAC | |
| 0120-R11 | CATAATGCTGGCAGACTTACCAGGTAAGTAT | |

## Supplementary Table 8 (continued).

| ASO name | ASO sequence (5′-3′) | Target |
|---|---|---|
| Etep | CTCCAACATCAAGGAAGATGGCATTTCTAG | |
| Etep-L6a | CAGGTACTCCAACATCAAGGAAGATGGCATTTCTAG | |
| Etep-L6b | AGGTAACTCCAACATCAAGGAAGATGGCATTTCTAG | |
| Etep-L6c | GGTAAGCTCCAACATCAAGGAAGATGGCATTTCTAG | |
| Etep-L6d | GTAAGTCTCCAACATCAAGGAAGATGGCATTTCTAG | |
| Etep-L6e | TAAGTACTCCAACATCAAGGAAGATGGCATTTCTAG | |
| Etep-L6f | AAGTATCTCCAACATCAAGGAAGATGGCATTTCTAG | |
| Etep-L7a | CAGGTAACTCCAACATCAAGGAAGATGGCATTTCTAG | |
| Etep-L7b | AGGTAAGCTCCAACATCAAGGAAGATGGCATTTCTAG | |
| Etep-L7c | GGTAAGTCTCCAACATCAAGGAAGATGGCATTTCTAG | |
| Etep-L7d | GTAAGTACTCCAACATCAAGGAAGATGGCATTTCTAG | |
| Etep-L7e | TAAGTATCTCCAACATCAAGGAAGATGGCATTTCTAG | |
| Etep-L8a | CAGGTAAGCTCCAACATCAAGGAAGATGGCATTTCTAG | |
| Etep-L8b | AGGTAAGTCTCCAACATCAAGGAAGATGGCATTTCTAG | |
| Etep-L8c | GGTAAGTACTCCAACATCAAGGAAGATGGCATTTCTAG | |
| Etep-L8d | GTAAGTATCTCCAACATCAAGGAAGATGGCATTTCTAG | |
| Etep-L9a | CAGGTAAGTCTCCAACATCAAGGAAGATGGCATTTCTAG | |
| Etep-L9b | AGGTAAGTACTCCAACATCAAGGAAGATGGCATTTCTAG | |
| Etep-L9c | GGTAAGTATCTCCAACATCAAGGAAGATGGCATTTCTAG | |
| Etep-L10a | CAGGTAAGTACTCCAACATCAAGGAAGATGGCATTTCTAG | *DMD* exon 51 |
| Etep-L10b | AGGTAAGTATCTCCAACATCAAGGAAGATGGCATTTCTAG | |
| Etep-L11 | CAGGTAAGTATCTCCAACATCAAGGAAGATGGCATTTCTAG | |
| Etep-R11 | CTCCAACATCAAGGAAGATGGCATTTCTAGCAGGTAAGTAT | |
| 000A | TCTAGTTTGGAGATGGCAGTT | |
| 000A-L7a | CAGGTAATCTAGTTTGGAGATGGCAGTT | |
| 000A-L7b | AGGTAAGTCTAGTTTGGAGATGGCAGTT | |
| 000A-L7c | GGTAAGTTCTAGTTTGGAGATGGCAGTT | |
| 000A-L7d | GTAAGTATCTAGTTTGGAGATGGCAGTT | |
| 000A-L7e | TAAGTATTCTAGTTTGGAGATGGCAGTT | |
| 000A-L8a | CAGGTAAGTCTAGTTTGGAGATGGCAGTT | |
| 000A-L8b | AGGTAAGTTCTAGTTTGGAGATGGCAGTT | |
| 000A-L8c | GGTAAGTATCTAGTTTGGAGATGGCAGTT | |
| 000A-L8d | GTAAGTATTCTAGTTTGGAGATGGCAGTT | |
| 000A-L9a | CAGGTAAGTTCTAGTTTGGAGATGGCAGTT | |
| 000A-L9b | AGGTAAGTATCTAGTTTGGAGATGGCAGTT | |
| 000A-L9c | GGTAAGTATTCTAGTTTGGAGATGGCAGTT | |
| 000A-L10a | CAGGTAAGTATCTAGTTTGGAGATGGCAGTT | |
| 000A-L10b | AGGTAAGTATTCTAGTTTGGAGATGGCAGTT | |
| 000A-L11 | CAGGTAAGTATTCTAGTTTGGAGATGGCAGTT | |

## Supplementary Table 8 (continued).

| ASO name | ASO sequence (5′-3′) | Target |
|---|---|---|
| Casimersen | CAATGCCATCCTGGAGTTCCTG | *DMD exon 45* |
| 002A | GTCGCCCTACCTCTTTTTTCTG | |
| 002A-L7a | CAGGTAAGTCGCCCTACCTCTTTTTTCTG | |
| 002A-L7b | AGGTAAGGTCGCCCTACCTCTTTTTTCTG | |
| 002A-L7c | GGTAAGTGTCGCCCTACCTCTTTTTTCTG | |
| 002A-L7d | GTAAGTAGTCGCCCTACCTCTTTTTTCTG | |
| 002A-L7e | TAAGTATGTCGCCCTACCTCTTTTTTCTG | |
| 002A-L8a | CAGGTAAGGTCGCCCTACCTCTTTTTTCTG | |
| 002A-L8b | AGGTAAGTGTCGCCCTACCTCTTTTTTCTG | |
| 002A-L8c | GGTAAGTAGTCGCCCTACCTCTTTTTTCTG | |
| 002A-L8d | GTAAGTATGTCGCCCTACCTCTTTTTTCTG | |
| 002A-L9a | CAGGTAAGTGTCGCCCTACCTCTTTTTTCTG | |
| 002A-L9b | AGGTAAGTAGTCGCCCTACCTCTTTTTTCTG | |
| 002A-L9c | GGTAAGTATGTCGCCCTACCTCTTTTTTCTG | |
| 002A-L10a | CAGGTAAGTAGTCGCCCTACCTCTTTTTTCTG | |
| 002A-L10b | AGGTAAGTATGTCGCCCTACCTCTTTTTTCTG | |
| 002A-L11 | CAGGTAAGTATGTCGCCCTACCTCTTTTTTCTG | |
| 002A-R7a | GTCGCCCTACCTCTTTTTTCTGCAGGTAA | |
| 002A-R7b | GTCGCCCTACCTCTTTTTTCTGAGGTAAG | |
| 002A-R7c | GTCGCCCTACCTCTTTTTTCTGGGTAAGT | |
| 002A-R7d | GTCGCCCTACCTCTTTTTTCTGGTAAGTA | |
| 002A-R7e | GTCGCCCTACCTCTTTTTTCTGTAAGTAT | |
| 002A-R8a | GTCGCCCTACCTCTTTTTTCTGCAGGTAAG | |
| 002A-R8b | GTCGCCCTACCTCTTTTTTCTGAGGTAAGT | |
| 002A-R8c | GTCGCCCTACCTCTTTTTTCTGGGTAAGTA | |
| 002A-R8d | GTCGCCCTACCTCTTTTTTCTGGTAAGTAT | |
| 002A-R9a | GTCGCCCTACCTCTTTTTTCTGCAGGTAAGT | |
| 002A-R9b | GTCGCCCTACCTCTTTTTTCTGAGGTAAGTA | |
| 002A-R9c | GTCGCCCTACCTCTTTTTTCTGGGTAAGTAT | |
| 002A-R10a | GTCGCCCTACCTCTTTTTTCTGCAGGTAAGTA | |
| 002A-R10b | GTCGCCCTACCTCTTTTTTCTGAGGTAAGTAT | |
| 002A-R11 | GTCGCCCTACCTCTTTTTTCTGCAGGTAAGTAT | |

## Supplementary Table 8 (continued).

| ASO name | ASO sequence (5′-3′) | Target |
|---|---|---|
| 1703 | CTGCAAAGAACACCTTGA | |
| 1703-L7a | CAGGTAACTGCAAAGAACACCTTGA | |
| 1703-L7b | AGGTAAGCTGCAAAGAACACCTTGA | |
| 1703-L7c | GGTAAGTCTGCAAAGAACACCTTGA | |
| 1703-L7d | GTAAGTACTGCAAAGAACACCTTGA | |
| 1703-L7e | TAAGTATCTGCAAAGAACACCTTGA | |
| 1703-L8a | CAGGTAAGCTGCAAAGAACACCTTGA | |
| 1703-L8b | AGGTAAGTCTGCAAAGAACACCTTGA | |
| 1703-L8c | GGTAAGTACTGCAAAGAACACCTTGA | |
| 1703-L8d | GTAAGTATCTGCAAAGAACACCTTGA | |
| 1703-L9a | CAGGTAAGTCTGCAAAGAACACCTTGA | |
| 1703-L9b | AGGTAAGTACTGCAAAGAACACCTTGA | |
| 1703-L9c | GGTAAGTATCTGCAAAGAACACCTTGA | |
| 1703-L10a | CAGGTAAGTACTGCAAAGAACACCTTGA | |
| 1703-L10b | AGGTAAGTATCTGCAAAGAACACCTTGA | |
| 1703-L11 | CAGGTAAGTATCTGCAAAGAACACCTTGA | *APP* |
| 1703-R7a | CTGCAAAGAACACCTTGACAGGTAA | |
| 1703-R7b | CTGCAAAGAACACCTTGAAGGTAAG | |
| 1703-R7c | CTGCAAAGAACACCTTGAGGTAAGT | |
| 1703-R7d | CTGCAAAGAACACCTTGAGTAAGTA | |
| 1703-R7e | CTGCAAAGAACACCTTGATAAGTAT | |
| 1703-R8a | CTGCAAAGAACACCTTGACAGGTAAG | |
| 1703-R8b | CTGCAAAGAACACCTTGAAGGTAAGT | |
| 1703-R8c | CTGCAAAGAACACCTTGAGGTAAGTA | |
| 1703-R8d | CTGCAAAGAACACCTTGAGTAAGTAT | |
| 1703-R9a | CTGCAAAGAACACCTTGACAGGTAAGT | |
| 1703-R9b | CTGCAAAGAACACCTTGAAGGTAAGTA | |
| 1703-R9c | CTGCAAAGAACACCTTGAGGTAAGTAT | |
| 1703-R10a | CTGCAAAGAACACCTTGACAGGTAAGTA | |
| 1703-R10b | CTGCAAAGAACACCTTGAAGGTAAGTAT | |
| 1703-R11 | CTGCAAAGAACACCTTGACAGGTAAGTAT | |

## Supplementary Table 8 (continued).

| ASO name | ASO sequence (5′-3′) | Target |
|---|---|---|
| ASO 1 | AGGCGGCGGAGTTCCTCA | *PKM* |
| ASO 1-L7a | CAGGTAAAGGCGGCGGAGTTCCTCA | |
| ASO 1-L7b | AGGTAAGAGGCGGCGGAGTTCCTCA | |
| ASO 1-L7c | GGTAAGTAGGCGGCGGAGTTCCTCA | |
| ASO 1-L7d | GTAAGTAAGGCGGCGGAGTTCCTCA | |
| ASO 1-L7e | TAAGTATAGGCGGCGGAGTTCCTCA | |
| ASO 1-L8a | CAGGTAAGAGGCGGCGGAGTTCCTCA | |
| ASO 1-L8b | AGGTAAGTAGGCGGCGGAGTTCCTCA | |
| ASO 1-L8c | GGTAAGTAAGGCGGCGGAGTTCCTCA | |
| ASO 1-L8d | GTAAGTATAGGCGGCGGAGTTCCTCA | |
| ASO 1-L9a | CAGGTAAGTAGGCGGCGGAGTTCCTCA | |
| ASO 1-L9b | AGGTAAGTAAGGCGGCGGAGTTCCTCA | |
| ASO 1-L9c | GGTAAGTATAGGCGGCGGAGTTCCTCA | |
| ASO 1-L10a | CAGGTAAGTAAGGCGGCGGAGTTCCTCA | |
| ASO 1-L10b | AGGTAAGTATAGGCGGCGGAGTTCCTCA | |
| ASO 1-L11 | CAGGTAAGTATAGGCGGCGGAGTTCCTCA | |
| ASO 1-R7a | AGGCGGCGGAGTTCCTCACAGGTAA | |
| ASO 1-R7b | AGGCGGCGGAGTTCCTCAAGGTAAG | |
| ASO 1-R7c | AGGCGGCGGAGTTCCTCAGGTAAGT | |
| ASO 1-R7d | AGGCGGCGGAGTTCCTCAGTAAGTA | |
| ASO 1-R7e | AGGCGGCGGAGTTCCTCATAAGTAT | |
| ASO 1-R8a | AGGCGGCGGAGTTCCTCACAGGTAAG | |
| ASO 1-R8b | AGGCGGCGGAGTTCCTCAAGGTAAGT | |
| ASO 1-R8c | AGGCGGCGGAGTTCCTCAGGTAAGTA | |
| ASO 1-R8d | AGGCGGCGGAGTTCCTCAGTAAGTAT | |
| ASO 1-R9a | AGGCGGCGGAGTTCCTCACAGGTAAGT | |
| ASO 1-R9b | AGGCGGCGGAGTTCCTCAAGGTAAGTA | |
| ASO 1-R9c | AGGCGGCGGAGTTCCTCAGGTAAGTAT | |
| ASO 1-R10a | AGGCGGCGGAGTTCCTCACAGGTAAGTA | |
| ASO 1-R10b | AGGCGGCGGAGTTCCTCAAGGTAAGTAT | |
| ASO 1-R11 | AGGCGGCGGAGTTCCTCACAGGTAAGTAT | |

## Supplementary Table 8 (continued).

| ASO name | ASO sequence (5′-3′) | Target |
|---|---|---|
| 7 X CUG | CTGCTGCTGCTGCTGCTGCTG | |
| 7 X CUG-L7a | CAGGTAACTGCTGCTGCTGCTGCTGCTG | |
| 7 X CUG-L7b | AGGTAAGCTGCTGCTGCTGCTGCTGCTG | |
| 7 X CUG-L7c | GGTAAGTCTGCTGCTGCTGCTGCTGCTG | |
| 7 X CUG-L7d | GTAAGTACTGCTGCTGCTGCTGCTGCTG | |
| 7 X CUG-L7e | TAAGTATCTGCTGCTGCTGCTGCTGCTG | |
| 7 X CUG-L8a | CAGGTAAGCTGCTGCTGCTGCTGCTGCTG | |
| 7 X CUG-L8b | AGGTAAGTCTGCTGCTGCTGCTGCTGCTG | |
| 7 X CUG-L8c | GGTAAGTACTGCTGCTGCTGCTGCTGCTG | |
| 7 X CUG-L8d | GTAAGTATCTGCTGCTGCTGCTGCTGCTG | |
| 7 X CUG-L9a | CAGGTAAGTCTGCTGCTGCTGCTGCTGCTG | |
| 7 X CUG-L9b | AGGTAAGTACTGCTGCTGCTGCTGCTGCTG | |
| 7 X CUG-L9c | GGTAAGTATCTGCTGCTGCTGCTGCTGCTG | |
| 7 X CUG-L10a | CAGGTAAGTACTGCTGCTGCTGCTGCTGCTG | |
| 7 X CUG-L10b | AGGTAAGTATCTGCTGCTGCTGCTGCTGCTG | |
| 7 X CUG-L11 | CAGGTAAGTATCTGCTGCTGCTGCTGCTGCTG | *ATXN3* |
| 7 X CUG-R7a | CTGCTGCTGCTGCTGCTGCTGCAGGTAA | |
| 7 X CUG-R7b | CTGCTGCTGCTGCTGCTGCTGAGGTAAG | |
| 7 X CUG-R7c | CTGCTGCTGCTGCTGCTGCTGGGTAAGT | |
| 7 X CUG-R7d | CTGCTGCTGCTGCTGCTGCTGGTAAGTA | |
| 7 X CUG-R7e | CTGCTGCTGCTGCTGCTGCTGTAAGTAT | |
| 7 X CUG-R8a | CTGCTGCTGCTGCTGCTGCTGCAGGTAAG | |
| 7 X CUG-R8b | CTGCTGCTGCTGCTGCTGCTGAGGTAAGT | |
| 7 X CUG-R8c | CTGCTGCTGCTGCTGCTGCTGGGTAAGTA | |
| 7 X CUG-R8d | CTGCTGCTGCTGCTGCTGCTGGTAAGTAT | |
| 7 X CUG-R9a | CTGCTGCTGCTGCTGCTGCTGCAGGTAAGT | |
| 7 X CUG-R9b | CTGCTGCTGCTGCTGCTGCTGAGGTAAGTA | |
| 7 X CUG-R9c | CTGCTGCTGCTGCTGCTGCTGGGTAAGTAT | |
| 7 X CUG-R10a | CTGCTGCTGCTGCTGCTGCTGCAGGTAAGTA | |
| 7 X CUG-R10b | CTGCTGCTGCTGCTGCTGCTGAGGTAAGTAT | |
| 7 X CUG-R11 | CTGCTGCTGCTGCTGCTGCTGCAGGTAAGTAT | |

## Supplementary Table 8 (continued).

| ASO name | ASO sequence (5′-3′) | Target |
|---|---|---|
| 016A | GAGTTCTTCAATTAGACTTT | |
| 016A-L7a | CAGGTAAGAGTTCTTCAATTAGACTTT | |
| 016A-L7b | AGGTAAGGAGTTCTTCAATTAGACTTT | |
| 016A-L7c | GGTAAGTGAGTTCTTCAATTAGACTTT | |
| 016A-L7d | GTAAGTAGAGTTCTTCAATTAGACTTT | |
| 016A-L7e | TAAGTATGAGTTCTTCAATTAGACTTT | |
| 016A-L8a | CAGGTAAGGAGTTCTTCAATTAGACTTT | |
| 016A-L8b | AGGTAAGTGAGTTCTTCAATTAGACTTT | |
| 016A-L8c | GGTAAGTAGAGTTCTTCAATTAGACTTT | |
| 016A-L8d | GTAAGTATGAGTTCTTCAATTAGACTTT | |
| 016A-L9a | CAGGTAAGTGAGTTCTTCAATTAGACTTT | |
| 016A-L9b | AGGTAAGTAGAGTTCTTCAATTAGACTTT | |
| 016A-L9c | GGTAAGTATGAGTTCTTCAATTAGACTTT | |
| 016A-L10a | CAGGTAAGTAGAGTTCTTCAATTAGACTTT | |
| 016A-L10b | AGGTAAGTATGAGTTCTTCAATTAGACTTT | |
| 016A-L11 | CAGGTAAGTATGAGTTCTTCAATTAGACTTT | *CEP290* |
| 016A-R7a | GAGTTCTTCAATTAGACTTTCAGGTAA | |
| 016A-R7b | GAGTTCTTCAATTAGACTTTAGGTAAG | |
| 016A-R7c | GAGTTCTTCAATTAGACTTTGGTAAGT | |
| 016A-R7d | GAGTTCTTCAATTAGACTTTGTAAGTA | |
| 016A-R7e | GAGTTCTTCAATTAGACTTTTAAGTAT | |
| 016A-R8a | GAGTTCTTCAATTAGACTTTCAGGTAAG | |
| 016A-R8b | GAGTTCTTCAATTAGACTTTAGGTAAGT | |
| 016A-R8c | GAGTTCTTCAATTAGACTTTGGTAAGTA | |
| 016A-R8d | GAGTTCTTCAATTAGACTTTGTAAGTAT | |
| 016A-R9a | GAGTTCTTCAATTAGACTTTCAGGTAAGT | |
| 016A-R9b | GAGTTCTTCAATTAGACTTTAGGTAAGTA | |
| 016A-R9c | GAGTTCTTCAATTAGACTTTGGTAAGTAT | |
| 016A-R10a | GAGTTCTTCAATTAGACTTTCAGGTAAGTA | |
| 016A-R10b | GAGTTCTTCAATTAGACTTTAGGTAAGTAT | |
| 016A-R11 | GAGTTCTTCAATTAGACTTTCAGGTAAGTAT | |

## Supplementary Table 8 (continued).

| ASO name | ASO sequence (5′-3′) | Target |
|---|---|---|
| ASO4 | CGTGTGGTGATTTTACCTTCAGTTG | |
| ASO4-L7a | CAGGTAACGTGTGGTGATTTTACCTTCAGTTG | |
| ASO4-L7b | AGGTAAGCGTGTGGTGATTTTACCTTCAGTTG | |
| ASO4-L7c | GGTAAGTCGTGTGGTGATTTTACCTTCAGTTG | |
| ASO4-L7d | GTAAGTACGTGTGGTGATTTTACCTTCAGTTG | |
| ASO4-L7e | TAAGTATCGTGTGGTGATTTTACCTTCAGTTG | |
| ASO4-L8a | CAGGTAAGCGTGTGGTGATTTTACCTTCAGTTG | |
| ASO4-L8b | AGGTAAGTCGTGTGGTGATTTTACCTTCAGTTG | |
| ASO4-L8c | GGTAAGTACGTGTGGTGATTTTACCTTCAGTTG | |
| ASO4-L8d | GTAAGTATCGTGTGGTGATTTTACCTTCAGTTG | |
| ASO4-L9a | CAGGTAAGTCGTGTGGTGATTTTACCTTCAGTTG | |
| ASO4-L9b | AGGTAAGTACGTGTGGTGATTTTACCTTCAGTTG | |
| ASO4-L9c | GGTAAGTATCGTGTGGTGATTTTACCTTCAGTTG | |
| ASO4-L10a | CAGGTAAGTACGTGTGGTGATTTTACCTTCAGTTG | |
| ASO4-L10b | AGGTAAGTATCGTGTGGTGATTTTACCTTCAGTTG | |
| ASO4-L11 | CAGGTAAGTATCGTGTGGTGATTTTACCTTCAGTTG | *MDM4* |
| ASO4-R7a | CGTGTGGTGATTTTACCTTCAGTTGCAGGTAA | |
| ASO4-R7b | CGTGTGGTGATTTTACCTTCAGTTGAGGTAAG | |
| ASO4-R7c | CGTGTGGTGATTTTACCTTCAGTTGGGTAAGT | |
| ASO4-R7d | CGTGTGGTGATTTTACCTTCAGTTGGTAAGTA | |
| ASO4-R7e | CGTGTGGTGATTTTACCTTCAGTTGTAAGTAT | |
| ASO4-R8a | CGTGTGGTGATTTTACCTTCAGTTGCAGGTAAG | |
| ASO4-R8b | CGTGTGGTGATTTTACCTTCAGTTGAGGTAAGT | |
| ASO4-R8c | CGTGTGGTGATTTTACCTTCAGTTGGGTAAGTA | |
| ASO4-R8d | CGTGTGGTGATTTTACCTTCAGTTGGTAAGTAT | |
| ASO4-R9a | CGTGTGGTGATTTTACCTTCAGTTGCAGGTAAGT | |
| ASO4-R9b | CGTGTGGTGATTTTACCTTCAGTTGAGGTAAGTA | |
| ASO4-R9c | CGTGTGGTGATTTTACCTTCAGTTGGGTAAGTAT | |
| ASO4-R10a | CGTGTGGTGATTTTACCTTCAGTTGCAGGTAAGTA | |
| ASO4-R10b | CGTGTGGTGATTTTACCTTCAGTTGAGGTAAGTAT | |
| ASO4-R11 | CGTGTGGTGATTTTACCTTCAGTTGCAGGTAAGTAT | |

**Supplementary Table 8 (continued).**

| ASO name | ASO sequence (5′-3′) | Target |
|---|---|---|
| 2707 | ATCCAGATGCCCTGG | |
| 2707-L7a | CAGGTAAATCCAGATGCCCTGG | |
| 2707-L7b | AGGTAAGATCCAGATGCCCTGG | |
| 2707-L7c | GGTAAGTATCCAGATGCCCTGG | |
| 2707-L7d | GTAAGTAATCCAGATGCCCTGG | |
| 2707-L7e | TAAGTATATCCAGATGCCCTGG | |
| 2707-L8a | CAGGTAAGATCCAGATGCCCTGG | |
| 2707-L8b | AGGTAAGTATCCAGATGCCCTGG | |
| 2707-L8c | GGTAAGTAATCCAGATGCCCTGG | |
| 2707-L8d | GTAAGTATATCCAGATGCCCTGG | |
| 2707-L9a | CAGGTAAGTATCCAGATGCCCTGG | |
| 2707-L9b | AGGTAAGTAATCCAGATGCCCTGG | |
| 2707-L9c | GGTAAGTATATCCAGATGCCCTGG | |
| 2707-L10a | CAGGTAAGTAATCCAGATGCCCTGG | |
| 2707-L10b | AGGTAAGTATATCCAGATGCCCTGG | |
| 2707-L11 | CAGGTAAGTATATCCAGATGCCCTGG | *ERBB2* |
| 2707-R7a | ATCCAGATGCCCTGGCAGGTAA | |
| 2707-R7b | ATCCAGATGCCCTGGAGGTAAG | |
| 2707-R7c | ATCCAGATGCCCTGGGGTAAGT | |
| 2707-R7d | ATCCAGATGCCCTGGGTAAGTA | |
| 2707-R7e | ATCCAGATGCCCTGGTAAGTAT | |
| 2707-R8a | ATCCAGATGCCCTGGCAGGTAAG | |
| 2707-R8b | ATCCAGATGCCCTGGAGGTAAGT | |
| 2707-R8c | ATCCAGATGCCCTGGGGTAAGTA | |
| 2707-R8d | ATCCAGATGCCCTGGGTAAGTAT | |
| 2707-R9a | ATCCAGATGCCCTGGCAGGTAAGT | |
| 2707-R9b | ATCCAGATGCCCTGGAGGTAAGTA | |
| 2707-R9c | ATCCAGATGCCCTGGGGTAAGTAT | |
| 2707-R10a | ATCCAGATGCCCTGGCAGGTAAGTA | |
| 2707-R10b | ATCCAGATGCCCTGGAGGTAAGTAT | |
| 2707-R11 | ATCCAGATGCCCTGGCAGGTAAGTAT | |

**Supplementary Table 9.** List of primer sequences used in this study.

| Target | Primer name | Sequence (5′-3′) |
| --- | --- | --- |
| *SMN1/2* | SMN-T7F2 | TACTTAATACGACTCACTATAGGCTAGCCTCG |
| | Ex8-29to52-R | Cy5-TCTGATCGTTTCTTTAGTGGTGTC |
| | Ex6-F | Cy5-ATAATTCCCCCACCACCTCCC |
| | Ex8-467-R | TTGCCACATACGCCTCACATAC |
| *DMD* exon 51 (RD cells) | Ex48-Ex49-F | GGACCATTTGACGTTAAGGAAACTG |
| | Ex53-22to43-R | Cy5-AGGTGTTCTTGTACTTCATCCC |
| *DMD* exon 51 (Δ52 mice) | Ex49-Ex50-F | Cy5-ACTCAGCCAGTGAAGAGGAAG |
| | Ex53-12to33-R | GAACCTCATCCCACTGAATCTG |
| *DMD* exon 51 (huΔ52 mice) | Ex50-81to102-F | AGCTCCTGGACTGACCACTATT |
| | Ex54-73to95-R | Cy5-GTATCATCAGCAGAATAGTCCCG |
| *DMD* exon 45 (RD cells) | Ex44-62to85-F | CAGTGGCTAACAGAAGCTGAACAG |
| | Ex46-63to87-R | Cy5-GCAATGTTATCTGCTTCCTCCAACC |
| *MET* | Forward | Cy5-GAAAATTGACTTAGCCAACCG |
| | Short-transcript reverse | TGGCTTCCTTATTTACATCATGAG |
| | Long-transcript reverse | TTCTCTTTTTCAGCCACAGG |
| *NR3C1* | Forward | Cy5-GCCAAGGATCTGGAGATGAC |
| | Short-transcript reverse | CCGTTGGCCAATGGGATAC |
| | Long-transcript reverse | GACATTTTCGATAGCGGCATG |
| *STK17A* | Forward | Cy5-GGCCAAGATTGTCGGATGGA |
| | Short-transcript reverse | TGAGATTTCCAGAGAACCAGGC |
| | Long-transcript reverse | AACAATCTTAATGTCACCCAATGGAG |
| *PPKAA2* | Forward | Cy5-CTGGTTGATAACAGGAGCTATCTT |
| | Reverse | CAGGCGAGGTGAAACTGAA |
| *MRPL18* | Forward | Cy5-GGCTTTTCCGAGATCGTCTCA |
| | Reverse | GGGTTCCGGTTGGTGAATTCT |
| *CDC34* | Forward | Cy5-GACGTGTGTATCTCCATCCTCC |
| | Reverse | CGTCCTCGTAGTAGTCGTCGT |
| *APP* | Forward | Cy5-CCAGCCAACACAGAAAACGAAG |
| | Reverse | CTAGTTCTGCATCTGCTCAAAGAAC |
| *ERBB2* | Forward primer | GATCCGGAAGTACACGATGC |
| | Reverse | Cy5-TCCCGGACATGGTCTAAGAG |
| *ATXN3* | Forward | Cy5-CCATAAAACAGACCTGGAACG |
| | Reverse | ACAGCTGCCTGAAGCATGTC |
| *CEP290* | Forward | TTTTAGAACTCCGGGCAGAA |
| | Reverse | Cy5-TTGGCTTGCCACTTTTTACC |

## Supplementary Table 9 (continued).

| Target | Primer name | Sequence (5′-3′) |
|---|---|---|
| *FOS* (qPCR) | FOS-F | TCTCCAGTGCCAACTTCATT |
| | FOS-R | GCTCTGGTCTGCGATGG |
| *MYC* (qPCR) | MYC-F | CATACATCCTGTCCGTCCAAG |
| | MYC-R | GAGTTCCGTAGCTGTTCAAGT |
| *RB1* (qPCR) | RB1-F | CAATCAAAGGACCGAGAAGGA |
| | RB1-R | GTGTGATTATTCTGGAGAGGAAGA |
| *ATR* (qPCR) | ATR-F | GCTGGTAGAAGAAGCAACATTTAG |
| | ATR-R | GGTACCCAGAATTGATGGAAGT |
| *GAPDH* (qPCR or sqRTPCR) | GAPDH-F (Cy5-5′ for sqRT-PCR) | AAGGTGAAGGTCGGAGTCAACGG |
| | GAPDH-R | CCACTTGATTTTGGAGGGATCTC |
| *DMD* exon 51 (huΔ52 mice/qPCR) | Ex50-Ex53-F | CCACTATTGGAGCCTTTGAAAG |
| | Ex53-107to126-R | AGACCTGCTCAGCTTCTTCC |

## SI References


1. S. Pankratova, B. N. Nielsen, T. Shiraishi, P. E. Nielsen, PNA-mediated modulation and redirection of Her-2 pre-mRNA splicing: specific skipping of erbB-2 exon 19 coding for the ATP catalytic domain. *Int J Oncol* **36**, 29-38 (2010).
2. M. Dewaele *et al.*, Antisense oligonucleotide-mediated MDM4 exon 6 skipping impairs tumor growth. *J Clin Invest* **126**, 68-84 (2016).
3. J. L. Chang *et al.*, Targeting amyloid-beta precursor protein, APP, splicing with antisense oligonucleotides reduces toxic amyloid-beta production. *Mol Ther* **26**, 1539-1551 (2018).
4. E. Kourkouta *et al.*, Suppression of mutant protein expression in SCA3 and SCA1 mice using a CAG repeat-targeting antisense oligonucleotide. *Mol Ther Nucleic Acids* **17**, 601-614 (2019).
5. W. K. Ma *et al.*, ASO-based PKM splice-switching therapy inhibits hepatocellular carcinoma growth. *Cancer Res* **82**, 900-915 (2022).
